# Electrocatalytic Hydrogenation of Pyridines and Other Nitrogen-Containing Aromatic Compounds

**DOI:** 10.1021/jacs.4c09107

**Published:** 2024-10-07

**Authors:** Naoki Shida, Yugo Shimizu, Akizumi Yonezawa, Juri Harada, Yuka Furutani, Yusuke Muto, Ryo Kurihara, Junko N. Kondo, Eisuke Sato, Koichi Mitsudo, Seiji Suga, Shoji Iguchi, Kazuhide Kamiya, Mahito Atobe

**Affiliations:** † Department of Chemistry and Life Science, Yokohama National University, 79-5 Tokiwadai, Hodogaya-ku, Yokohama 240-8501, Japan; ‡ Institute of Advanced Sciences, Yokohama National University, 79-5 Tokiwadai, Hodogaya-ku, Yokohama 240-8501, Japan; § PRESTO, Japan Science and Technology Agency (JST), 4-1-8 Honcho, Kawaguchi, Saitama 332-0012, Japan; ∥ Research Center for Solar Energy Chemistry, Graduate School of Engineering Science, Osaka University, 1−3 Machikaneyama, Toyonaka, Osaka 560-8531, Japan; ⊥ Institute of Innovative Research, Tokyo Institute of Technology, 4259 Nagatsuta, Midori-ku, Yokohama, Kanagawa 225-8503, Japan; # Division of Applied Chemistry, Graduate School of Environmental, Life, Natural Science and Technology, Okayama University, 3-1-1 Tsushima-naka, Kita-ku, Okayama 700-8530, Japan; ∇ Graduate School of Engineering, Kyoto University, Kyoto daigaku-katsura, Nishikyo-ku, Kyoto 615-8530, Japan; ○ Innovative Catalysis Science Division, Institute for Open and Transdisciplinary Research Initiatives (ICS-OTRI), Osaka University, Suita, Osaka 565-0871, Japan

## Abstract

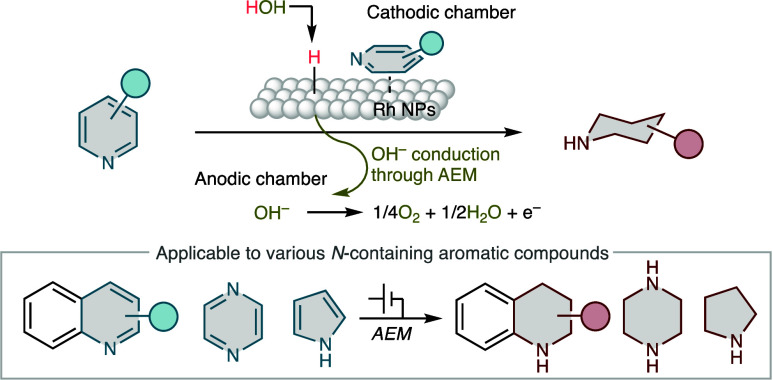

The production of cyclic amines, which are vital to the pharmaceutical industry, relies on energy-intensive thermochemical hydrogenation. Herein, we demonstrate the electrocatalytic hydrogenation of nitrogen-containing aromatic compounds, specifically pyridine, at ambient temperature and pressure via a membrane electrode assembly with an anion-exchange membrane. We synthesized piperidine using a carbon-supported rhodium catalyst, achieving a current density of 25 mA cm^–2^ and a current efficiency of 99% under a circular flow until 5 F mol^–1^. Quantitative conversion of pyridine into piperidine with 98% yield was observed after passing 9 F mol^–1^, corresponding to 65% of current efficiency. The reduction of Rh oxides on the catalyst surface was crucial for catalysis. The Rh(0) surface interacts moderately with piperidine, decreasing the energy required for the rate-determining desorption step. The proposed process is applicable to other nitrogen-containing aromatic compounds and could be efficiently scaled up. This method presents clear advantages over traditional high-temperature and high-pressure thermochemical catalytic processes.

## Introduction

According to a report by the International Energy Agency, the chemical industry is the largest consumer of energy, ranking third in terms of carbon emissions.^1^ As energy conservation in chemical processes is critical for achieving a sustainable society, the shift from traditional thermal processes to electrochemical processes is urgently needed. The utilization of renewable energy sources in electrochemical processes can lead to sustainable chemical processes with reduced energy consumption and carbon emissions. Consequently, research has been conducted to electrify various organic chemical reactions.^2−5^

**Figure 1:**
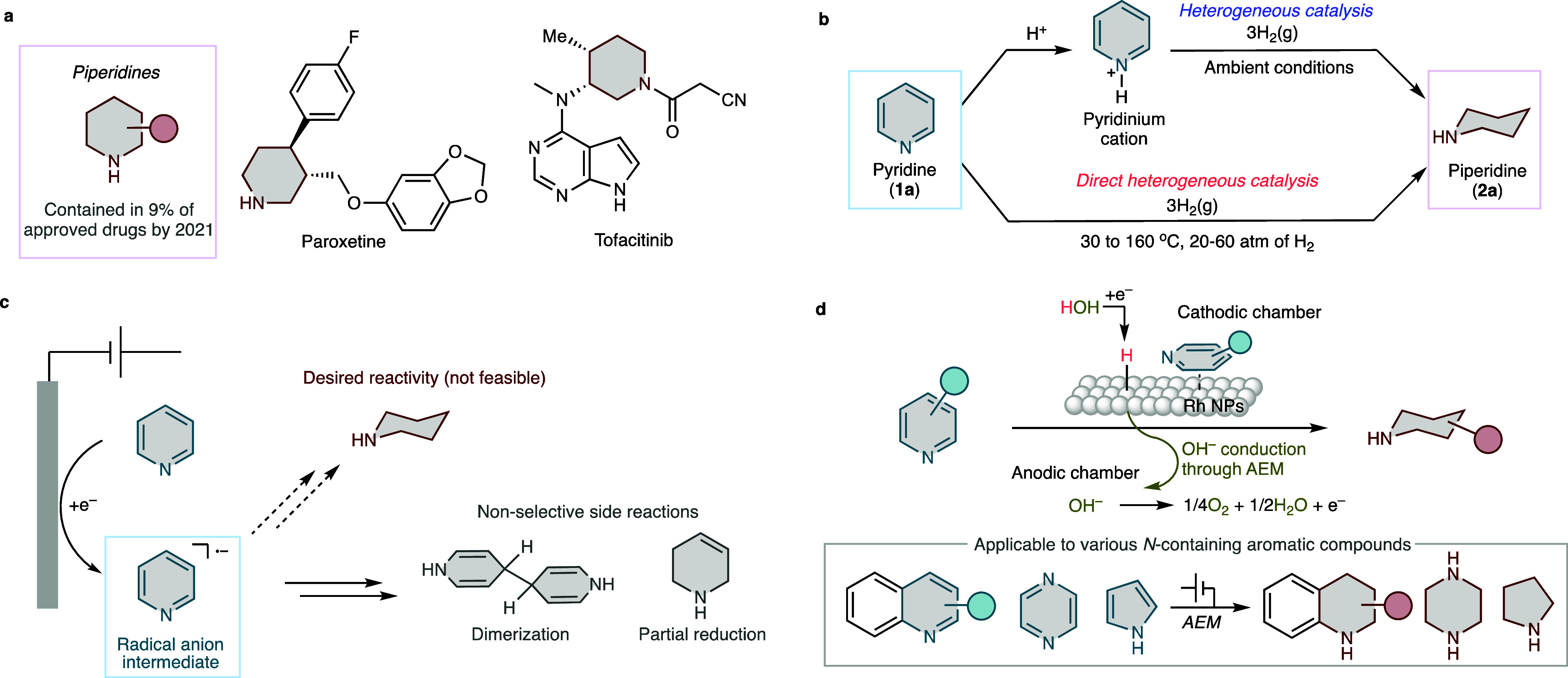
Synthesis of piperidines via the hydrogenation of pyridines. (a) Examples of FDA-approved drugs containing the piperidine skeleton. (b) Thermal hydrogenation of pyridine to piperidine using a heterogeneous catalyst. (c) Electrochemical reduction of pyridines in a conventional electrolytic system via a radical anion intermediate. (d) Concept of this work.

Cyclic amines are essential building blocks for fine chemicals.^6^ For example, piperidine is one of the most common skeletons in drugs approved by the Food and Drug Administration (FDA; Figure 1a).^7,8^ These compounds are also used in pesticides, functional polymeric material monomers, and other compounds that are integral to daily life. A typical synthetic method for piperidine involves the catalytic hydrogenation of the corresponding aromatic compound, pyridine, using H_2_ gas as a source of protons and electrons.^6^ Although various small molecular catalysts have been developed,^9−12^ heterogeneous catalysts have been actively developed owing to their applicability in industrial processes.^9,13−19^ Pyridine can be easily reduced under mild conditions by activating the pyridinium cation using acid.^17,20^ However, acid activation is not ideal because it increases waste production, production costs, and reactor corrosion. The hydrogenation of pyridine without acid activation generally requires elevated temperatures, pressurized H_2_ gas, or both of them, even with state-of-the-art heterogeneous catalysts (Figure 1b).^18,21−23^ Thus, the hydrogenation of pyridine and its derivatives under mild conditions without the use of acidic additives remains challenging. In addition, these processes rely on the use of H_2_ gas as the reductant. Currently, most of the H_2_ gas in the market is gray hydrogen, i.e., hydrogen obtained through the steam reforming of methane, which is responsible for ∼3% of the global emission of CO_2_.^24,25^

In this context, the electrochemical hydrogenation of pyridines has attracted significant attention (Figure 1c).^26−28^ In 1896, Ahrens gave an early example of the electrochemical reduction of pyridine using a lead cathode in 10% sulfuric acid.^29^ Some groups have recently reported the formation of piperidine through the electrochemical reduction of pyridinium cations.^30,31^ However, no reliable methodology for the electrochemical hydrogenation of pyridine to piperidine with high energy efficiency, scalability, and sustainability has yet been reported. A plausible reason for this deficiency is that the electrochemical reduction of pyridine via outer-sphere single-electron transfer produces a highly reactive radical anion intermediate, leading to undesired side reactions such as dimerization and partial hydrogenation rather than the desired 6e^–^/6H^+^ hydrogenation (Figure 1c).^9^ This fact suggests that the electrochemical hydrogenation of pyridine should rather rely on electrocatalytic hydrogenation, in which electrochemically generated adsorbed hydrogen species (*H*_ads_) react with organic molecules without generating ionic intermediates.

Herein, we report the electrocatalytic hydrogenation of nitrogen-containing aromatic compounds, particularly pyridines (Figure 1d). Using an anion-exchange membrane (AEM) electrolyzer equipped with carbon-supported Rh as the cathode catalyst, the hydrogenation of various pyridines was achieved at ambient temperature and pressure without any additives in the catholyte. This system is also applicable to quinoline, pyrazine, and pyrrole, demonstrating its versatility for various nitrogen-containing aromatic compounds. This paper proposes an innovative method for synthesizing valuable cyclic amines with high energy efficiency under mild conditions, thus contributing to the sustainable production of fine chemicals.

## Results and Discussion

### Reaction in the AEM Electrolyzer

**Figure 2:**
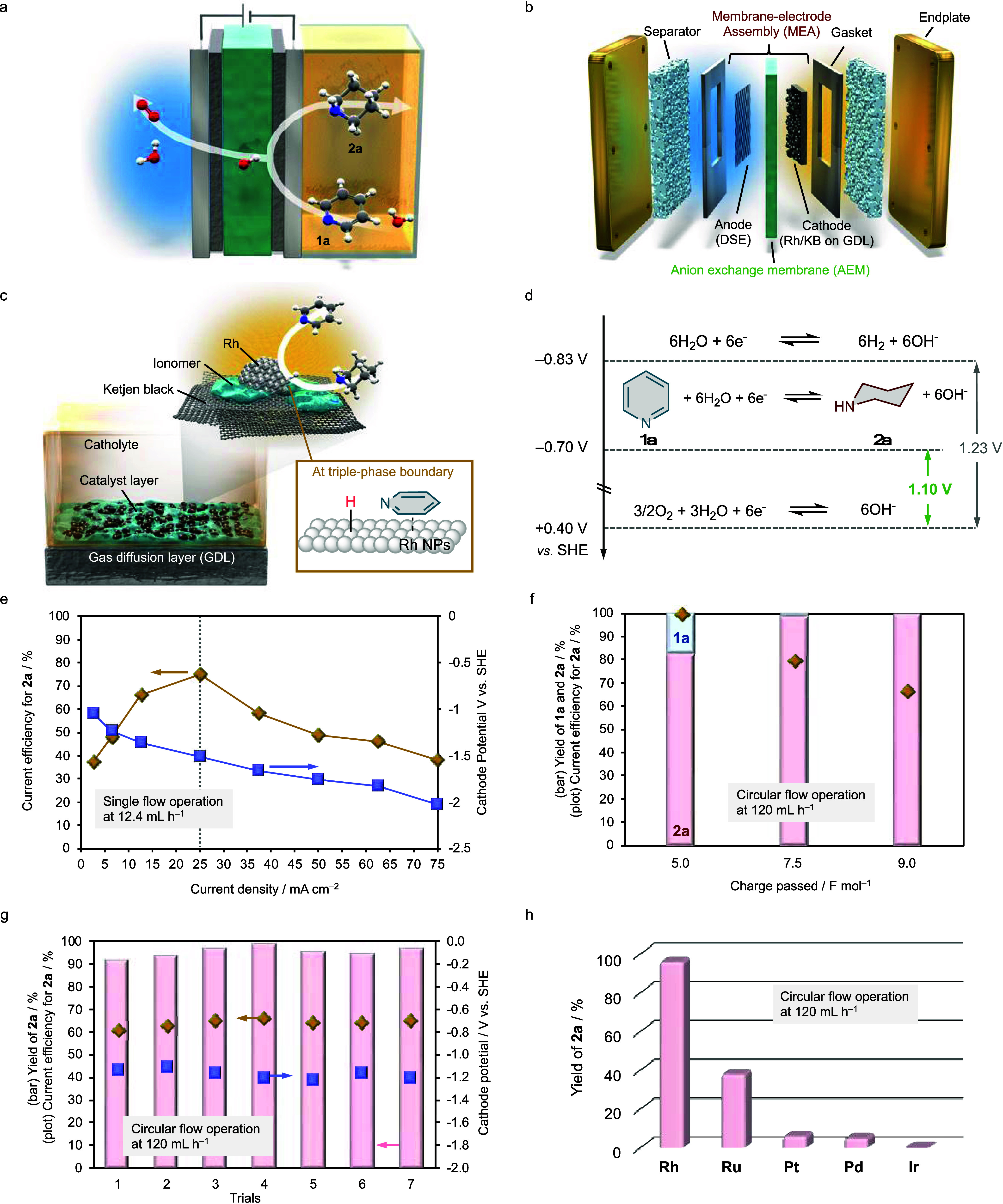
Overview of the electrochemical hydrogenation of pyridine (**1a**) to piperidine (**2a**) in an AEM electrolyzer. (a) Schematic illustration and (b) detailed components of the AEM electrolyzer. (c) Enlarged image of the catalyst layer and triple-phase boundary. (d) Energy diagram of the cathodic and anodic reactions. (e) Plot of the current efficiency and cathodic potential for the electrochemical hydrogenation of **1a** under various current densities. Electrolysis was conducted under single-flow operation at a flow rate of 12.4 mL h^–1^ in an AEM electrolyzer equipped with a Rh/KB. (f) Preparative electrocatalytic hydrogenation of **1a** under circular-flow operation at a flow rate of 120 mL h^–1^. (g) Repeated preparative electrocatalytic hydrogenation of **1a** under circular-flow operation at a flow rate of 120 mL h^–1^. (h) Comparison of various metal catalysts under circular-flow operation using MTBE as a cathodic solvent.

In this study, an AEM electrolyzer was used for the electrochemical hydrogenation of pyridine (**1a**) to piperidine (**2a**). AEM electrolyzers are widely used in H_2_O electrolysis^32^ and CO_2_ reduction,^33^ but are rarely applied to the electrosynthesis of fine chemicals.^34−36^Figure 2a–c show the setup of the AEM electrolyzer, a detailed description of the system components, and an enlarged image of the reaction site, i.e., the triple-phase boundary, respectively. Rh nanoparticles supported on Ketjen black (Rh/KB, loading amount: 0.5 mg cm^–2^) were used as the cathode catalyst and immobilized on the gas diffusion electrodes using an anion-exchange ionomer. A membrane electrode assembly (MEA) was constructed by sandwiching an AEM between the cathode and dimensionally stable electrode (DSE) anode to form a zero-gap configuration (Figures 2b and S1). A catholyte containing **1a** and H_2_O was then introduced into the cathodic chamber. *H*_ads_ is formed via the electrochemical reduction of H_2_O at the triple-phase boundary, concomitant with the generation of OH^–^. *H*_ads_ react with the adsorbed **1a** to produce **2a** (Figure 2c). OH^–^ migrates to the anodic chamber through the AEM and is oxidized to release O_2_ (Figures S2 and S3). This setup does not require an anolyte solution. In addition, nonaqueous organic solvents can also be used as catholytes, which are beneficial when the substrate is insoluble in aqueous media. In this case, the anolyte must contain H_2_O as a source of protons and electrons for *H*_ads_ formation at the cathode (Figures S4 and S5).

### Thermodynamics of Pyridine Hydrogenation

A thermodynamic comparison was made between the thermal and electrocatalytic hydrogenation of pyridine using H_2_O as the source of electrons and protons (Figure 2d). In a waste-free and sustainable thermal hydrogenation system, we postulate that H_2_ gas is obtained from electrolysis with renewable energy (green hydrogen). The voltage required to produce green hydrogen, which is independent of pH, is 1.23 V. The hydrogenation of pyridine with H_2_ is theoretically exothermic, although most instances of the heterogeneous hydrogenation of pyridine are performed under elevated temperatures and pressurized conditions. Thus, the energy input required for the thermal process using green hydrogen is 1.23 V.

On the other hand, the overall reaction formula for the hydrogenation of pyridine under basic conditions is shown in eqs 1–3.


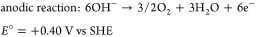
1
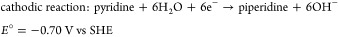
2
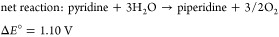
3Therefore, the electrocatalytic hydrogenation of pyridine proceeds with less energy than thermal hydrogenation using green hydrogen. Moreover, electrocatalytic hydrogenation at ambient temperature and pressure offers additional advantages over thermal processes in terms of energy consumption.

### Electrocatalytic Hydrogenation of Pyridine

We performed the electrolysis using H_2_O as the catholyte prior to electrolysis. This pre-electrolysis treatment effectively improved the current efficiency and reproducibility of the system. Subsequently, a 100 mM aqueous solution of **1a** was injected into the cathodic chamber using a syringe pump, and constant-current electrolysis was performed. When electrolysis was carried out at various currents with a fixed flow rate of 12.4 mL h^–1^, **2a** was successfully obtained in a wide range of current densities (Figure 2e). The current efficiency profile peaked at 25 mA cm^–2^, reaching 75%. The decrease in current efficiency at higher currents was attributed to the concurrent hydrogen evolution reaction (HER), which is associated with a more negative cathode potential. Intriguingly, the low current efficiency was also observed at a lower current density, which may be due to the cathode potential being too positive, resulting in a smaller Gibbs free energy change for the electrochemical step (see First-principles Calculations section for related discussion).

Although many electrocatalytic studies have focused on the current efficiency with low conversion of the starting material, the product yield with complete conversion of the starting material should also be investigated, especially if the production of high-value chemicals such as pharmaceuticals is to be implemented. Therefore, preparative electrolytic synthesis was performed with a circular catholyte flow. Electrolysis was performed at 25 mA cm^–2^ under a circular catholyte flow with a rate of 120 mL h^–1^, and an electric charge of up to 9 F mol^–1^ was passed (Figure 2f). **1a** was mostly consumed after passing 7.5 F mol^–1^, at which point only 1.2% of **1a** was observed, and completely disappeared at 9 F mol^–1^. After passing 9 F mol^–1^, the desired product, **2a**, was obtained in quantitative yield with a current efficiency of 66% (Figure 2f). Interestingly, the current efficiency was 99% at 5 F mol^–1^ and retained to 79% at 7.5 F mol^–1^. These values are even higher than those obtained in the single-flow experiment shown in Figure 2e, plausibly because of the difference in flow rate. These data suggest that this system enables the electrocatalytic hydrogenation of pyridine to piperidine with high energy efficiency even in a preparative-scale reaction. The data obtained herein was applied to the comparison with previously reported acid-free heterogeneous thermal catalytic systems (Table S1). The electrochemical system presented herein enabled the hydrogenation of pyridine into piperidine with a comparable production rate under much milder conditions. During the reaction, the formation of side products such as 1,2,3,6-tetrahydropyridine was not detected by gas chromatography analysis at any time point of electrolysis, and only **2a** was observed as a product. Thus, it is suggested that the reaction proceeded directly from **1a** to **2b** without generating any side product.

To gain mechanistic insights, the electrolysis was performed using pyridine-*d*_6_ and D_2_O (Scheme S1, and Figures S24–S29). Gas chromatography–mass spectroscopy analysis of the reaction mixture indicated the formation of piperidine-*d*_11_, where the mass spectrum of synthesized piperidine-*d*_11_ corresponded to that of authentic piperidine-*d*_11_. This experiment demonstrates that water is a source of hydrogen atoms in the present electrocatalytic hydrogenation system.

Catalyst recyclability was examined under similar conditions (Figure 2g). After conducting electrolysis seven times under a circular flow, the starting material was completely consumed in all cases, and piperidine was obtained, with **2a** yields of over 90% in all trials. Therefore, the high durability of the proposed system was successfully demonstrated.

Subsequently, catalyst screening was performed (Figure 2h). In addition to Rh/KB, platinum (Pt)-group metal (PGM) catalysts such as Ru/KB, Pt/KB, Pd/KB, and Ir/KB were used, and electrolysis was conducted for 36 min under a circular catholyte flow using methyl *tert*-butyl ether (MTBE) as the catholyte solvent. The results revealed that the use of PGM catalysts other than Rh resulted in a significant decrease in piperidine yield. This finding indicates that Rh/KB is a catalyst with uniquely high activity for the electrochemical hydrogenation of pyridine.

### Electrochemical Measurements

**Figure 3:**
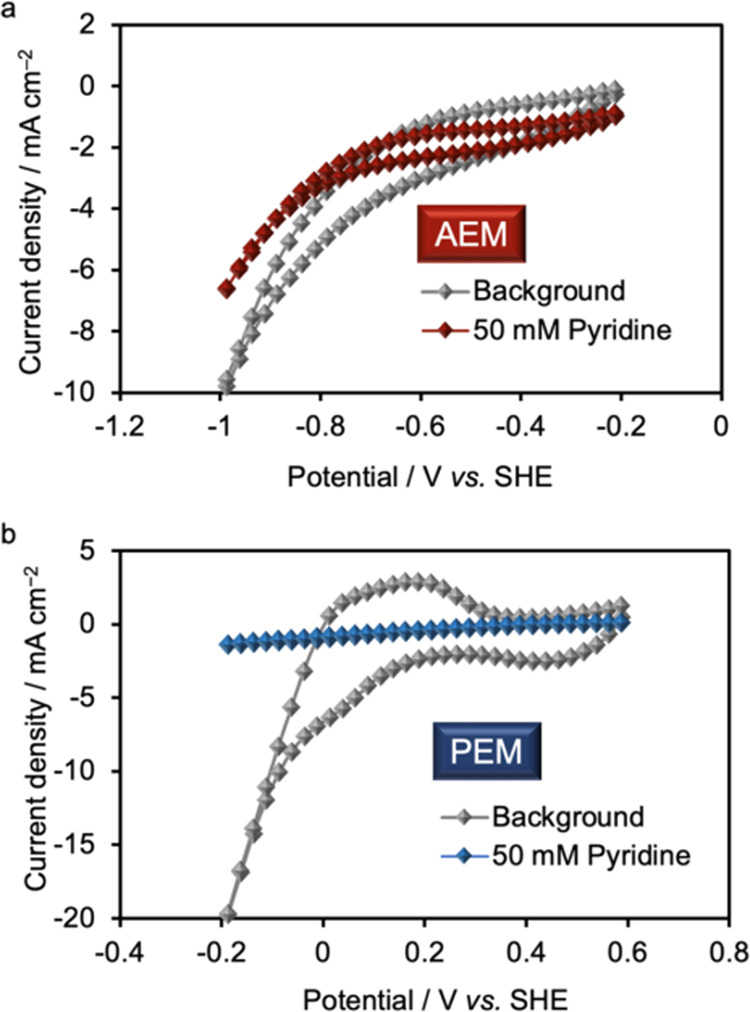
Electrochemical measurements. Comparison of the CVs recorded in the (a) AEM and (b) PEM electrolyzer at a scan rate of 10 mV s^–1^. The measurements were performed in the absence (black) and presence (red or blue) of **1a**.

Electrochemical measurements were performed using the AEM electrolyzer (Figure 3a). For comparison, the results obtained using a proton exchange membrane (PEM) electrolyzer are also presented (Figure 3b).

Rh/KB was used as the cathode catalyst in both systems. When the AEM electrolyzer was used, the onset of a reduction current associated with hydrogen evolution was observed at approximately −0.8 V vs a standard hydrogen electrode (SHE) in the absence of **1a**. Upon the addition of **1a**, the current decreased slightly, and the passage of electricity was confirmed. By contrast, in the PEM electrolyzer, the cathodic current decreased significantly in the presence of pyridine, likely because pyridine is protonated to form pyridinium salts in the acidic reaction environment of the PEM electrolyzer, inhibiting ion conduction through the PEM. Because nitrogen-containing aromatic compounds are basic, their reduction in the PEM electrolyzer may be impractical. Therefore, the use of an AEM electrolyzer, which provides a basic reaction environment, is essential for the energy-efficient reduction of nitrogen-containing aromatic compounds without the use of additives.

### In Situ X-ray Absorption Fine Structure Measurements

**Figure 4:**
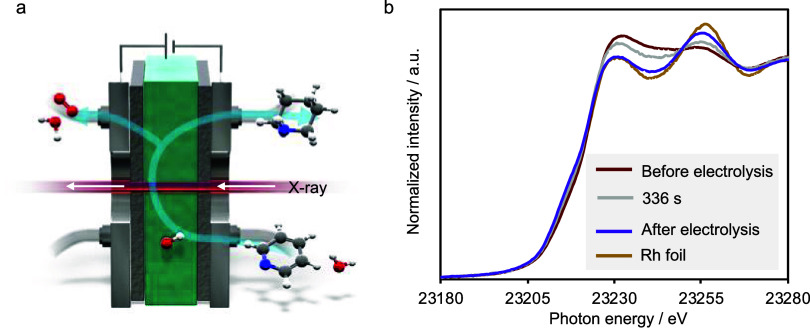
In situ XAFS measurements. (a) Schematic illustration of the AEM electrolyzer with a transmitting window for X-ray. (b) XANES spectra obtained during 640 s of electrolysis using H_2_O as the catholyte. The XANES spectrum of Rh foil is also shown as a reference.

The electrolysis of **1a** revealed that the current efficiency of **2a** improved after pre-electrolysis with H_2_O. To gain insights into the reduction behavior of Rh/KB during electrolysis, we performed Rh K-edge in situ X-ray absorption fine structure (XAFS) measurements using a tailor-made electrolytic cell equipped with polyimide windows for the X-rays (Figure 4a). In situ XAFS measurement was performed during the 640 s of electrolysis using water as a catholyte (Figures S9 and S10). Linear combination fitting analysis of the Rh K-edge X-ray absorption near edge structure (XANES) spectrum of the Rh/KB catalyst before electrolysis clearly suggested that the Rh species were partially oxidized; the ratio of Rh(0) to Rh(III) was 62:38 (Figure 4b, red). When electrolysis was performed using H_2_O as the catholyte, the XANES spectrum of the Rh species changed, showing an isosbestic point, and Rh was nearly completely reduced to metallic Rh (Figure 3b). These data correspond to the results of X-ray photoelectron spectroscopy (XPS) measurements. Before electrolysis, the XPS spectrum of Rh/KB showed the presence of Rh(0) and Rh(III) (Figure S7a). By contrast, the postelectrolysis sample of Rh/KB showed the predominant presence of Rh(0) species (Figure S7b). This finding suggests that the surface of the oxide layer of Rh/KB is reduced to expose the catalytically active Rh(0) surface during pre-electrolysis treatment.

### First-principles Calculations

After confirming that Rh(0) is the catalytically active species in the system, we conducted density functional theory calculations of pyridine hydrogenation on the Rh(0) surface to elucidate the relevant mechanism. For comparison, calculations were also performed for Pt, which showed lower catalytic activity than Rh.

First, calculations were performed for the adsorption process. We calculated the adsorption energy of flat and vertical configurations. The adsorption via the pyridine π-plane (flat configuration) was most stable, which corresponded with the previous report (Figures S11 and S12).^37^ Although the adsorption energies of pyridine on Rh (2.20 eV) and Pt (2.27 eV) were similar, Pt was slightly more stable. Therefore, the energy calculations for the adsorption stage do not explain the difference in reactivity between Rh and Pt.

**Figure 5:**
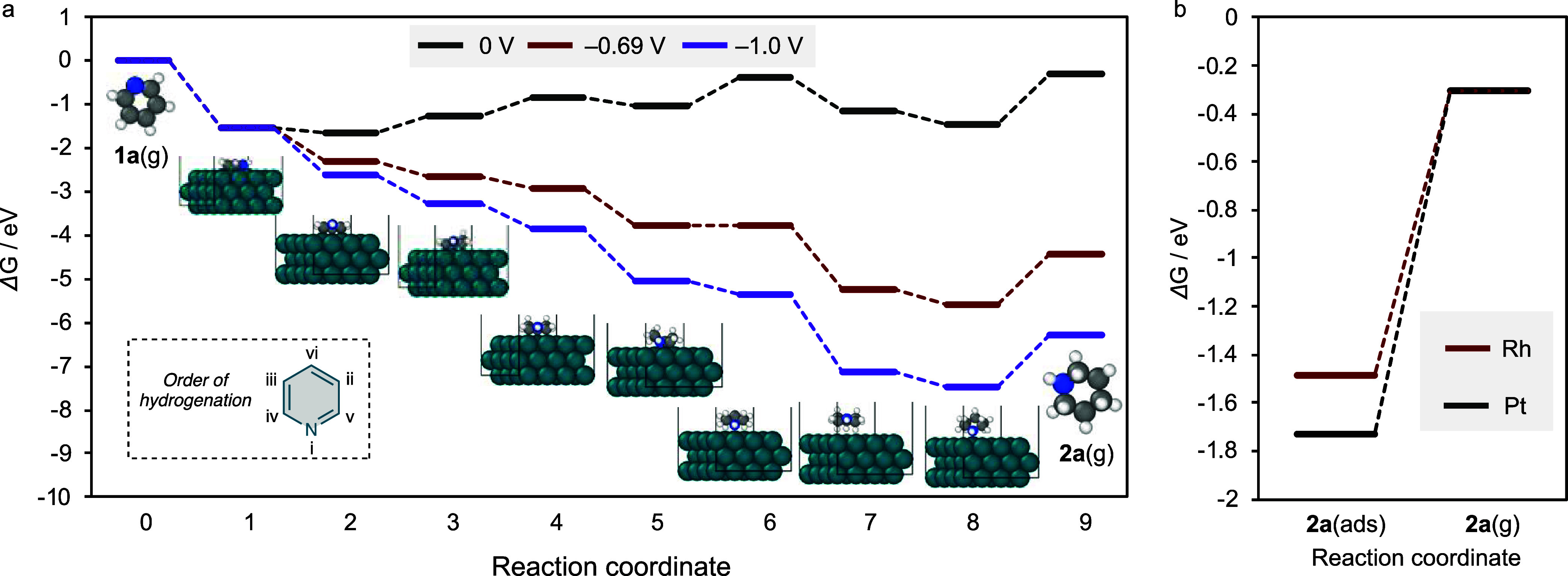
Summary of the computational simulations for the electrochemical hydrogenation of pyridine. (a) Energy diagram for the adsorption, 6e^–^/6H^+^ reduction of **1a**, and desorption process of **2a** over the Rh catalyst. The order of hydrogenation position in **1a** was defined according to the reported system.^37^ (b) Energy diagram for the desorption of **2a** from the Rh (red) and Pt (black) surface.

Next, we obtained a free-energy diagram for the pyridine hydrogenation process to elucidate the key steps. Li et al. reported comprehensive calculations on the hydrogenation of pyridine adsorbed by its π-plane on Pt surfaces, and proposed a reaction sequence starting from *N*-hydrogenation.^37^ Based on this report, we calculated the energy of each intermediate in the pyridine hydrogenation process on the Rh(0) surface. As shown in Figure 5a, steps 8 and 9 (desorption of piperidine, nonelectron transfer steps) and steps 5 and 6 (electron transfer steps) exhibit high energy barriers of 1.18 and 0.69 eV, respectively, at 0 V.

Notably, under applied potential conditions, the product or intermediate states are stabilized based on the concept of a computational hydrogen electrode (CHE). The chemical potential of a proton–electron pair G(H^+^ + e^–^) is equivalent to one-half of the chemical potential of gaseous hydrogen [0.5G(H_2_)] at 0 V vs a reversible hydrogen electrode and the energy–e*U*, where *U* is the external potential (eq 4).
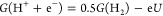
4When we computationally applied potentials of −0.69 and −1.0 V vs CHE (Figure 5a), most of the elementary steps became exergonic. The theoretical onset potential, which is defined here as the potential at which all electron-transfer steps become exergonic, was −0.69 V for pyridine hydrogenation on the Rh(0) surface.

In contrast to the electron-transfer steps, the desorption of piperidine, a nonelectron-transfer step, remained endergonic (1.18 eV). The calculated desorption barrier decreased from 1.18 eV (25 °C) to 0.98 eV (50 °C) (Figures S14 and S15), indicating that the reaction could be facilitated at elevated temperatures. When the desorption steps of piperidine on Rh and Pt were compared, Rh showed a lower desorption barrier (1.18 eV) than Pt (1.43 eV) (Figure 5b). These results suggest that Rh exhibits extremely high catalytic activity compared with Pt because of the smooth progression of the product desorption process.

### Expansion of the Synthetic Utility of the Proposed System

Subsequently, the substrate scope was examined. Electrosynthesis is generally evaluated on the basis of two criteria: current efficiency and yield. Current efficiency is important when emphasizing the energy efficiency of production and economic costs associated with electricity. However, yield becomes more significant when synthesizing high-value-added compounds, such as active pharmaceutical ingredients. This study focused on yields assuming the use of the proposed process in pharmaceutical production. Data on the current efficiency for small charge amounts are compiled in Figures S16–S23.

Table 1 summarizes the scope and limitations of the proposed system. A slight excess charge was required for pyridines that were simply substituted with a methyl or ethyl group, but the target compounds were obtained in very high yields (**1b**–**f**). The reaction proceeded smoothly with lutidine (**1f**), and the cis isomer was obtained diastereoselectively. Both nicotinamide (**1g**) and isonicotinamide (**1h**) successfully afforded the corresponding piperidine derivatives. Although nicotinic acid methyl ester (**1i**) gave the hydrogenated product in 7% yield, nicotinic acid (**1j**) afforded the corresponding piperidine derivative (**2j**) in 44% yield. Hydrogenation of pyridines with a 4-CF_3_ (**1k**) or 2-Ph (**1l**) group were performed using methyl *tert*-butyl ether (MTBE) as cathodic solvent due to the solubility issue in water. Reaction using **1k** and **1l** afforded desired product in relatively lower yield, although an excess charge afforded the desired compounds. The lower yields for **2j,k** could be attributed both to the nature of the substrate or the effect of the solvent. The reaction progress using **1l** was sluggish but proceeded sufficiently at an elevated temperature (50 °C). These findings correspond to the implications of the computational results.

**Table 1:**
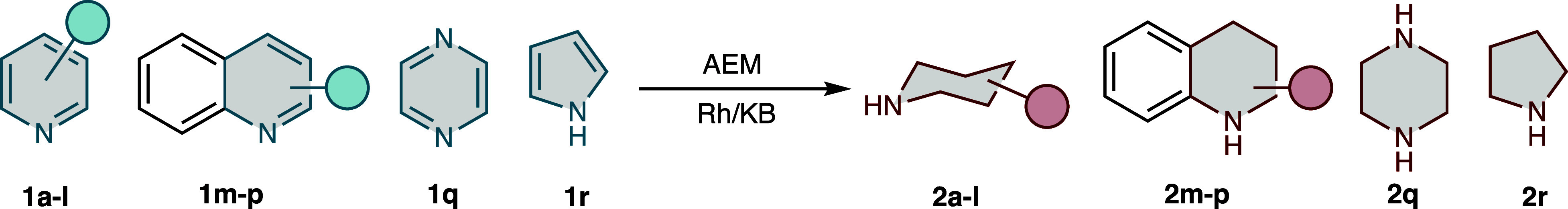

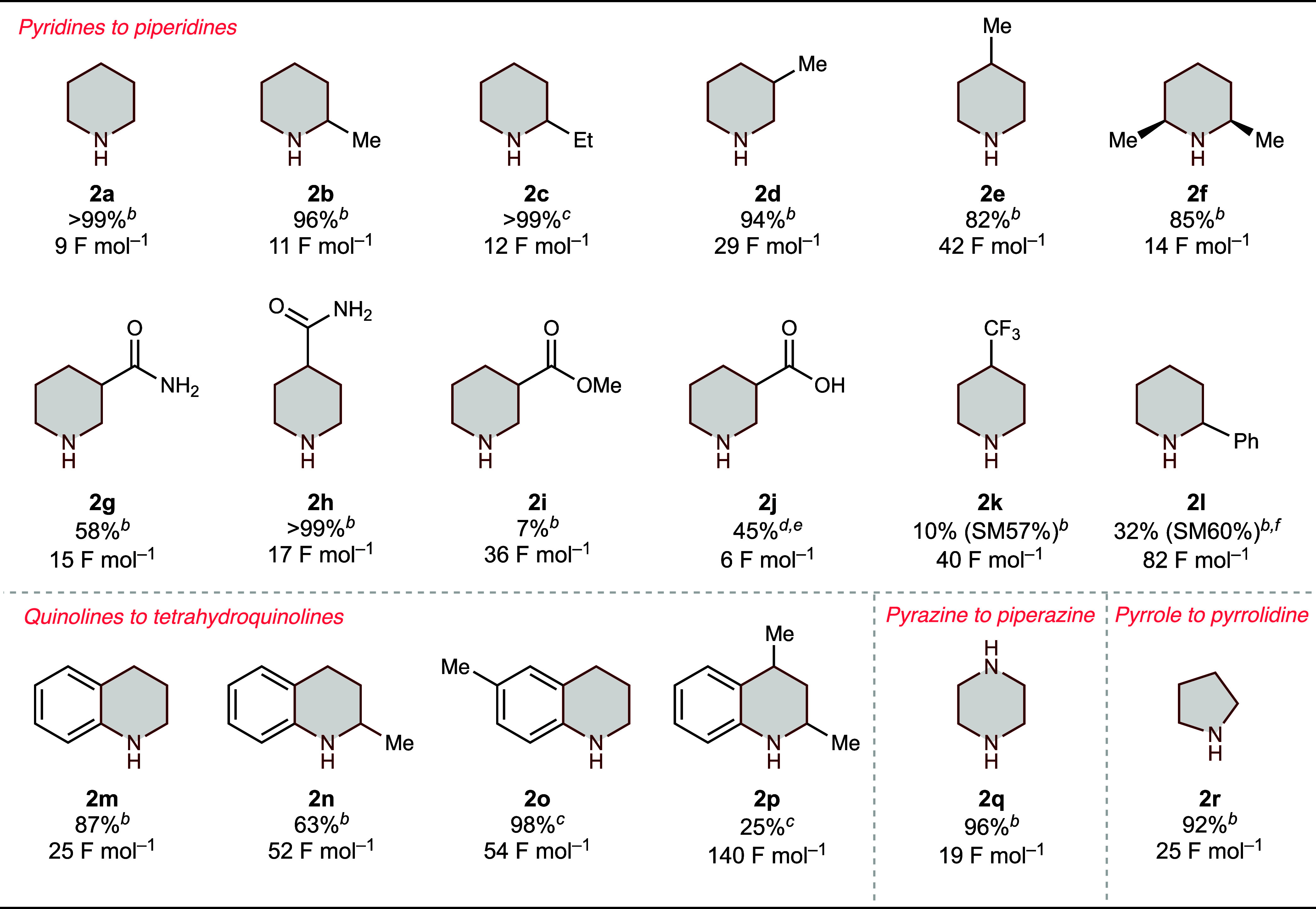
Substrate scope for the electrocatalytic hydrogenation of various nitrogen-containing aromatic compounds in an AEM electrolyzer equipped with a Rh/KB catalyst^a^

a Experimental conditions: catholyte, 5 mL solution of 100 mM (**1a–l**, **1q–r)** or 50 mM (**1m–p**) substrate in water (**1a–j**, **1q**), water/THF = 1/1 in vol. (**1r**), or MTBE (**1k–p**); anolyte, air (for aqueous systems) or 10 mM KOH (for nonaqueous systems); current density, 25 mA cm^–2^; anode, DSE anode; temperature, 25 °C.

b Determined by GC.

c Determined by ^1^H NMR.

d Determined by HPLC.

e 50 mA cm^–2^.

f 50 °C. SM = starting material.

The reaction also proceeded smoothly with quinoline derivatives, and the desired tetrahydroisoquinolines were obtained in high yields (**1m**–**p**). The electrocatalytic hydrogenation of quinoline via a fluorine-modified cobalt catalyst in an alkaline medium was recently reported,^38^ but no reports on its application in electrolysis without a supporting electrolyte in the catholyte, as presented in this study, have been published. Our system was also applicable to pyrazines (**1q**), providing piperazines (**2q**) in high yield. Pyrazine is a redox-active molecule that produces 1,4-dihydropyrazine through a 2e^–^/2H^+^ reduction process,^39,40^ but no other examples of the electrochemical synthesis of piperazine, a 6e^–^/6H^+^ reduction product, have been reported. In addition, we performed the hydrogenation of pyrrole (**1r**). Compared with pyridine, pyrrole features an electron-rich aromatic ring; thus, its electrochemical reduction is generally more challenging. To our delight, **1r** was quantitatively reduced to pyrrolidine (**2r**) in our system. Tetrahydroquinoline, piperazine, and pyrrolidine are the skeletons found in many approved drugs.^7,8^ These examples demonstrate that an AEM electrolyzer equipped with Rh/KB could not only enable the hydrogenation of a wide range of nitrogen-containing aromatic compounds but also synthesize cyclic amines in high yields under ambient conditions. To the best of our knowledge, this study is the first to report the electrochemical hydrogenation of pyrazine to piperazine and pyrrole to pyrrolidine.

Electrocatalytic hydrogenations were also performed using 2-vinylpyridine, 2-ethynylpyridine, and indole, respectively, under the following reaction conditions (Figure S30). For 2-vinylpyridine and 2-ethynylpyridine, hydrogenation of both aromatic ring and double or triple bond occurred simultaneously, and 2-ethylpyridine was obtained as the sole product in both cases. It is noteworthy that the current efficiency of 2-ethynylpyridine was much lower compared to 2-vinylpyridine or 2-ethylpyridine, presumably due to the weaker adsorption of the starting material. Electrochemical hydrogenation of indole scarcely proceeded under the conditions shown below. These experiments give deeper insights into the mechanism and limitations of the system.

**Figure 6:**
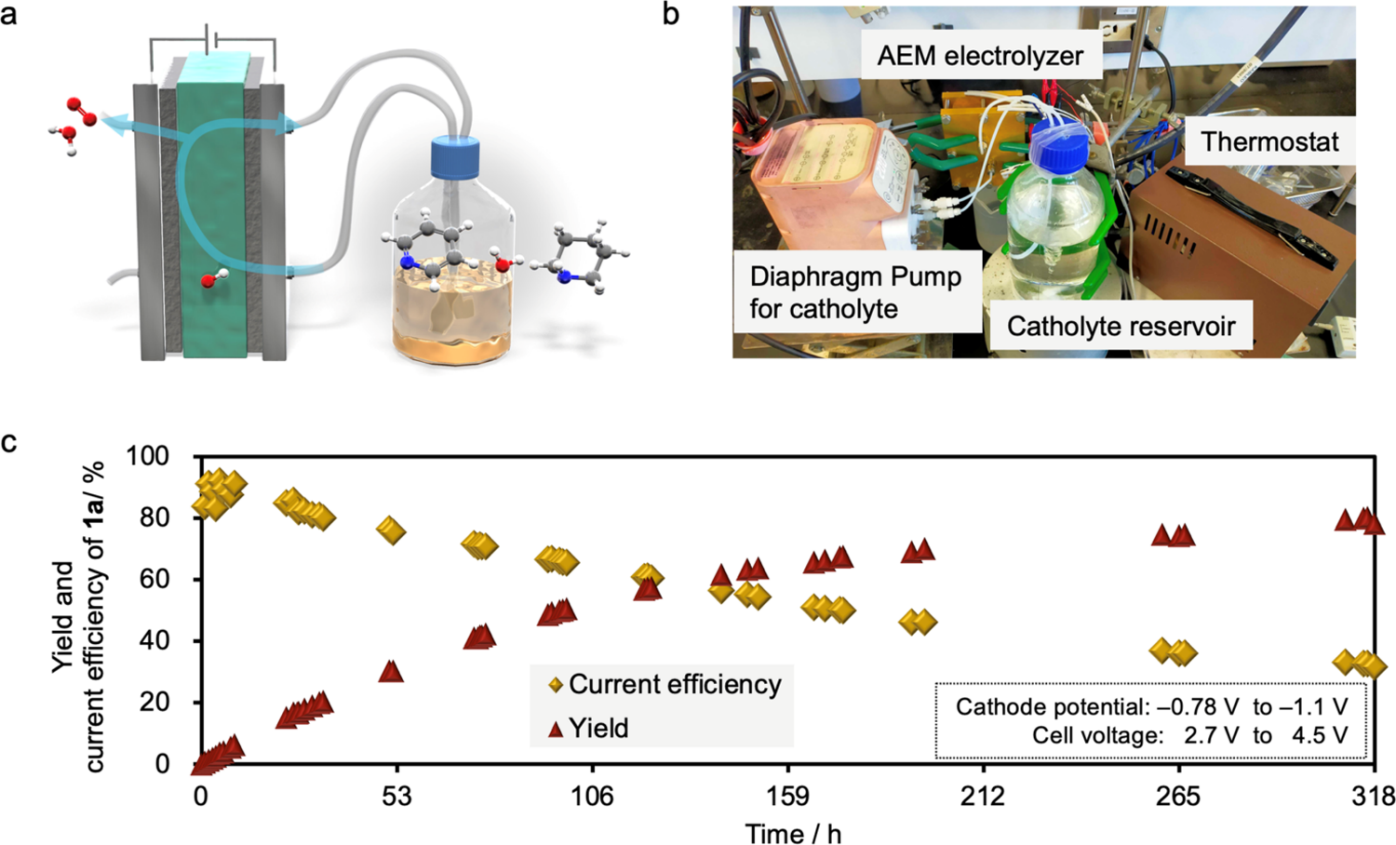
Large-scale electrolysis of **1a** using an AEM electrolyzer. (a) Schematic illustration and (b) photograph of the experimental setup. (c) Plot of the yield and current efficiency of **2a** as a function of the reaction time.

Then, large-scale electrolysis was performed to demonstrate the scalability of the proposed system (Figure 6a,b). Large-scale electrolysis was performed using 800 mL of an aqueous solution containing 6.3 g (80 mmol) of **1a**. Electrolysis was carried out at 25 mA cm^–2^ under circular-flow operation at a flow rate of 120 mL h^–1^. After 318 h of electrolysis, 5.3 g (63 mmol, 78% yield) **2a** was obtained. Importantly, the remaining 20% of **1a** was also confirmed, suggesting the absence of significant side reactions, except the HER. During electrolysis, the cathode potential remained in the range of −0.78 to −1.1 V vs SHE, and the cell voltage increased from 2.7 to 4.5 V. These results demonstrate that our system is extremely robust and suitable for the large-scale synthesis of nitrogen-containing aromatic compounds (Figure 6c).

## Conclusions

In this paper, we reported the electrocatalytic hydrogenation of pyridines and other nitrogen-containing aromatic compounds via electrolytic reduction using an AEM electrolyzer. The Rh/KB catalyst exhibited extremely high activity, allowing for the electrolytic hydrogenation of pyridines in quantitative yields with up to 99% current efficiency in the preparative reaction. In situ XAFS measurements confirmed that the oxide layer on the surface of the Rh nanoparticle catalyst was deoxygenated under electrolytic conditions to expose the Rh(0) surface, which acted as an active electrocatalyst. Furthermore, first-principles calculations revealed that the desorption of the product, piperidine, was the rate-determining step, and that Rh showed lower energy than Pt. Substrate investigations demonstrated that this system can be applied to a variety of pyridine and quinoline analogs. This study also presents the first successful complete electrochemical hydrogenation of pyrazine and pyrrole. In large-scale trials, we successfully obtained 5.3 g of the hydrogenated product from 6.3 g of the starting material, with a yield of 78%. This result demonstrates the scalability of the proposed system. The large-scale electrolysis showed a slight increase in the cell voltage during the 318 h of electrolysis, plausibly due to the increased resistance at AEM. The development of AEM is advancing rapidly with their use in water electrolysis and CO_2_ electrolysis.^32,41,42^ Implementing advanced AEM, or more ideally, developing AEM specifically for organic electrosynthesis, could overcome the problem observed in this work.

Cyclic amines are an important group of compounds found in many pharmaceuticals, and their direct synthesis involves the reduction of nitrogen-containing aromatic compounds. Several methods for the thermocatalytic hydrogenation of nitrogen-containing compounds has been reported; however, such processes require high temperatures, high pressures, and additives such as acids. Very few reports on the electrochemical reduction of nitrogen-containing aromatic compounds have been published; specifically, no methodology for the electrochemical reduction of pyridine to piperidine has yet been introduced. This paper proposes the first practical electrochemical system that appropriately utilizes the basic reaction field of an AEM electrolyzer to reduce nitrogen-containing aromatic compounds, thus contributing to energy savings and waste reduction in the synthesis of pharmaceutical precursors. Further development of the current system, including an AEM electrolytic system using earth-abundant metals and application to real pharmaceutical precursors, is undergoing in our laboratory.

## Supporting Information Available

General methods, description of the electrolyzer and experimental procedure, details of computation, chlormatgraphy data, and ^1^H NMR spectra (PDF)
