# Supplementary material for: Electrocatalytic Hydrogenation of Pyridines and Other Nitrogen-Containing Aromatic Compounds
Source: J Am Chem Soc. 2024 Oct 7;146(44):30212–21. doi: 10.1021/jacs.4c09107 (PMC11544711; doi:10.1021/jacs.4c09107)
Supplement: Supplementary file 1 — ja4c09107_si_001.pdf [file ja4c09107_si_001.pdf]

Supporting Information

Electrocatalytic Hydrogenation of Pyridines and Other Nitrogen-containing Aromatic Compounds

Naoki Shida,[1,2,3] Yugo Shimizu,[1] Akizumi Yonezawa,[1] Juri Harada,[1] Yuka Furutani,[1] Yusuke Muto,[1] Ryo Kurihara,[4] Junko N. Kondo,[5] Eisuke Sato,[6] Koichi Mitsudo,[6] Seiji Suga,[6] Shoji Iguchi,[7] Kazuhide Kamiya,[4,8] Mahito Atobe[1,2*]*

[1] Department of Chemistry and Life Science, Yokohama National University, 79-5 Tokiwadai, Hodogaya-ku, Yokohama 240-8501, Japan

[2] Institute of Advanced Sciences, Yokohama National University, 79-5 Tokiwadai, Hodogaya-ku, Yokohama 240-8501, Japan

[3] PRESTO, Japan Science and Technology Agency (JST), 4-1-8 Honcho, Kawaguchi, Saitama 332-0012, Japan

[4] Research Center for Solar Energy Chemistry, Graduate School of Engineering Science, Osaka University, 1–3 Machikaneyama, Toyonaka, Osaka 560-8531, Japan

[5] Institute of Innovative Research, Tokyo Institute of Technology, 4259 Nagatsuta, Midori-ku, Yokohama, Kanagawa 225-8503, Japan

[6] Division of Applied Chemistry, Graduate School of Environmental, Life, Natural Science and Technology, Okayama University, 3-1-1 Tsushima-naka, Kita-ku, Okayama, 700-8530, Japan

[7] Graduate School of Engineering, Kyoto University, Kyoto daigaku-katsura, Nishikyo-ku, Kyoto 615-8530, Japan

[8] Innovative Catalysis Science Division, Institute for Open and Transdisciplinary Research Initiatives (ICS-OTRI), Osaka University, Suita, Osaka 565-0871, Japan

E-mail: shida-naoki-gz@ynu.ac.jp, atobe@ynu.ac.jp

Table of contents

1. ***General methods***
2. ***Preparation of MEAs***
3. ***AEM reactor assembly***
4. ***Constant-current electrolysis in an AEM reactor with aqueous solution for catholyte***
5. ***Constant-current electrolysis in an AEM reactor with a non-aqueous solution for catholyte and aqueous solution for anolyte***
6. ***Comparison of acid-free hydrogenation of pyridine using heterogeneous catalysts***
7. ***Voltammetry measurements***
8. ***Analysis of Rh/KB catalyst***
9. ***In situ XAFS measurement***
10. ***Thermodynamic calculation of direct electrochemical hydrogenation***
11. ***Computational details***
12. ***Current efficiency and yield over time***
13. ***Additional data for substrate scope study***
14. ***Hydrogenation of pyridine-d_5 using D_2O***
15. ***Gas chromatograms***
16. ***High-performance liquid chromatograms***
17. ***NMR spectral data***
18. ***Supplementary References***

1. General methods

Reagents and solvents were purchased from commercial sources and used without further purifications unless otherwise noted. Anion-exchange membranes (AEMs, A201) and anion ionomer (AS-4) were kindly supplied from Tokuyama Corporation. Fuel cell catalysts TEC10E50E; Pt/Ketjenblack (Pt/KB; 46.1 wt.%), TECRh(ONLY)E30; Rh/Ketjenblack (Rh/KB; 28.9 wt.%), TECRu(ONLY)E30; Ru/Ketjenblack (Ru/KB; 27.0 wt.%), TECPd(ONLY)E30; and Pd/Ketjenblack (Pd/KB; 29.3 wt.%) were purchased from Tanaka Kikinzoku Kogyo (TKK). Carbon paper used as a gas-diffusion layer (GDL, Sigracet® GDL39AA, GDL39BB) was purchased from SGL CARBON. The DSE® anode for oxygen evolution was kindly supplied by De Nora Permelec.

Electrolysis under single-flow operation was performed with syringe pumps (KDS100, KD Scientific). Electrolysis under continuous-flow operation was carried out with diaphragm pumps (Q-100-TT-P-S, Tacmina). Constant-current electrolyses were conducted using a multichannel potentiogarvanostat (VSP-3e, Biologic). Voltammetric analyses were performed on an electrochemical analyzer (ALS/CH Instruments 660E, BAS). An Ag/AgCl electrode (RE-1BP, EC Frontier; RE-1B, BAS) was used as a reference electrode.

Gas chromatography (GC-FID) analyses for **1a-1i**, **1k-1n** and **1q-1r** were performed using a Shimadzu gas chromatograph (GC2014) equipped with a CP-Sil 8 CB for amines (Agilent Technologies) capillary column. Helium was used as a carrier gas for the GC analyses. High-performance liquid chromatography (HPLC) analyses for **1j** were performed using a LC pump (LC-20AD, Shimadzu Co.), a UV detector (SPD-20A, Shimadzu), and a reverse-phase column (Mightysil RP18GPII250-4.6, Kanto Chemical) under isothermal conditions at 40 °C, with H_2O/CH_3CN = 95/5 with 20 mM phosphoric acid as the mobile phase. ^{1}H NMR spectra for **1o-1p** were recorded on a JOEL ECA500 (500 MHz) spectrometer using tetramethylsilane (TMS) as an internal standard with the solvent resonance ($CDCl_3$: δ 7.26, D_2O: δ 4.79). X-ray photoelectron spectroscopy (XPS) measurements were performed using a JPS-9010MC instrument (JEOL) equipped with an Al Kα radiation source (12 kV, 25 mA). The analyzer pass energy was 30 eV. Corrective action of the samples was carried out by setting the binding energy of adventitious carbon (C 1s) at 284.8 eV. X-ray diffraction (XRD) patterns were recorded with an Ultima IV X-ray diffractometer (Rigaku) equipped with a Cu Kα radiation source (40 kV, 20 mA). The XRD patterns were recorded in the range $10° \leq 2\theta \leq 100°$ with a step size of 0.05°. Transmission electron

microscopy (TEM) measurements were carried out with a JEM-2100F microscope (JEOL) operated at 200 kV. Current efficiency was calculated as follows.

$$\text{Current efficiency [\%]} = \frac{\text{Number of charge used for the reaction of interest [C]}}{\text{Number of charge passed throughout the electrolysis [C]}} \times 100$$

2. Preparation of MEAs

Carbon-supported metal catalysts, deionized water, ionomer, and 1-propanol were mixed as a catalyst dispersion to make the ratio of ionomer to carbon to be 0.8:1. Carbon separator used as a GDL was sprayed with catalyst dispersion on one side. Then, catalyst-coated carbon paper was dried for 20 min at 40 °C. One sheet of this catalyst layer (1 cm × 4 cm) was used for the cathode. An anion exchange membrane was put between the cathode and anode (DSE) with catalyst-coated sides facing the membrane. Rh, Ru, Pt, Pd, and Ir were used as cathode catalyst materials as well as the anode without metal catalyst for the blank experiment. The loading amount of each metal catalyst was 0.5 mg cm^{-2}.

3. AEM reactor assembly

To assemble an AEM electrolyzer, gold-plated stainless steel end plates, carbon separators, Teflon® gaskets, and an MEA were assembled and the screws (M6) were tightened to 3.0 N·m using a torque wrench (Figure S1).

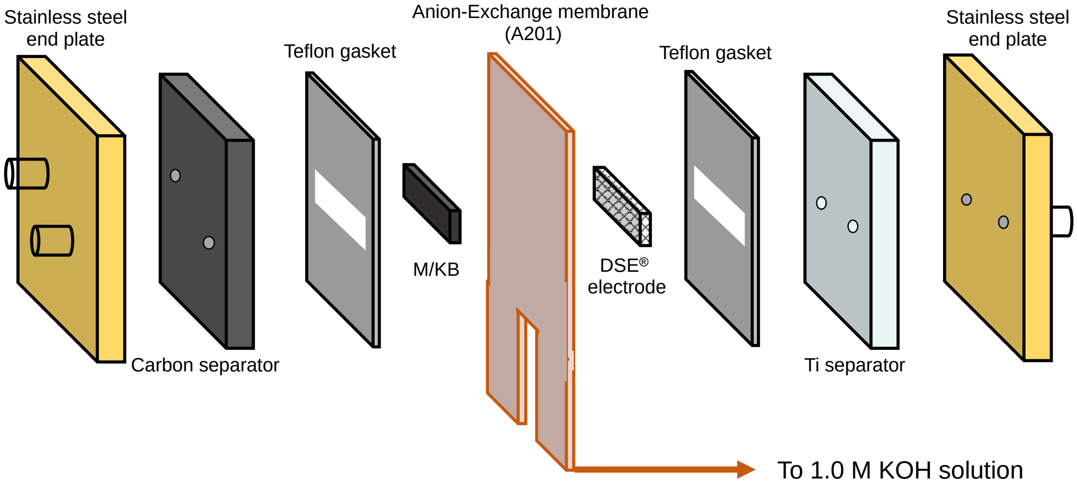

Figure S1 Assembly of an AEM reactor

4. Constant-current electrolysis in an AEM reactor with aqueous solution for catholyte

Prior to the electrolysis using substrate, water electrolysis, *i.e.*, pre-electrolysis, was performed at 50 mA cm^{-2} under the circular flow of deionized water as catholyte. The pre-electrolysis was performed for 2 hours in most experiments, while it could be shortened to 10 min without influencing the reaction outcome, as suggested in in situ XAFS measurement. Then, the reaction solution containing substrate was introduced and circulated at a flow rate of 2.0 mL min^{-1} to replace the catholyte solution left in pre-electrolysis (Figure S2).

The summary of the overall reaction for an aqueous solvent for the cathode is described in Figure S3.

For the single flow electrolysis experiment shown in Figure 2e in the manuscript, 100 mM aqueous solution of pyridine (**1a**) was introduced to a cathodic chamber by syringe pump at a flow rate of 12.4 mL h^{-1}. Constant-current electrolysis at 2.5, 6.25, 12.5, 25, 30, 37.5, and 50 mA cm^{-2} were performed. Diglyme was added to the collected sample after electrolysis as an internal standard. The sample was then diluted with methanol (4-fold dilution) and subjected to GC analysis.

For the circular flow experiment shown in Figs. 2f and 2g in the manuscript, 5 mL of 100 mM pyridine (**1a**) aqueous solution was introduced into a cathodic chamber at a flow rate of 120 mL h^{-1} by diaphragm pump, and constant current electrolysis was performed at 25 mA cm^{-2} for the desired time (Figure S4). Diglyme was added to the collected sample after electrolysis as an internal standard. The sample was then diluted with methanol (4-fold dilution) and subjected to GC analysis.

For the substrate screening experiment shown in Table 1 in the manuscript, 5 mL of 100 mM solution in water (**1a-j**, **1q**) or water/THF = 1/1 in vol. (**1r**) was circulated to the cathode side at a flow rate of 120 mL h^{-1} by diaphragm pump, and constant current electrolysis was performed at 25 mA cm^{-2} for desired time (Figure S4). The reactions were all performed at 25 °C. After electrolysis, the samples were analyzed by GC (diglyme as an internal standard) or HPLC.

Note:

The reason for the use of an internal standard for GC analysis of AEM electrolysis in an aqueous solvent system is described as follows. Water used as a cathodic solvent also functions as a source of hydrogen atoms. Thus, water reduction reaction occurs at the cathode to generate H_{ads}

concomitant with the generation of OH^-, consuming water during the electrolysis. In addition to that, when OH^- is transported from the cathode to the anode, it accompanies water molecules. This phenomenon also reduces the amount of water in the catholyte. For these reasons, the water in catholyte decreases with the progress of electrolysis, *i.e.*, the concentration of the substrate changes. Therefore, the external standard method, which determines the yield according to the concentration of the substrate, cannot derive the correct value, and the internal standard method was adopted.

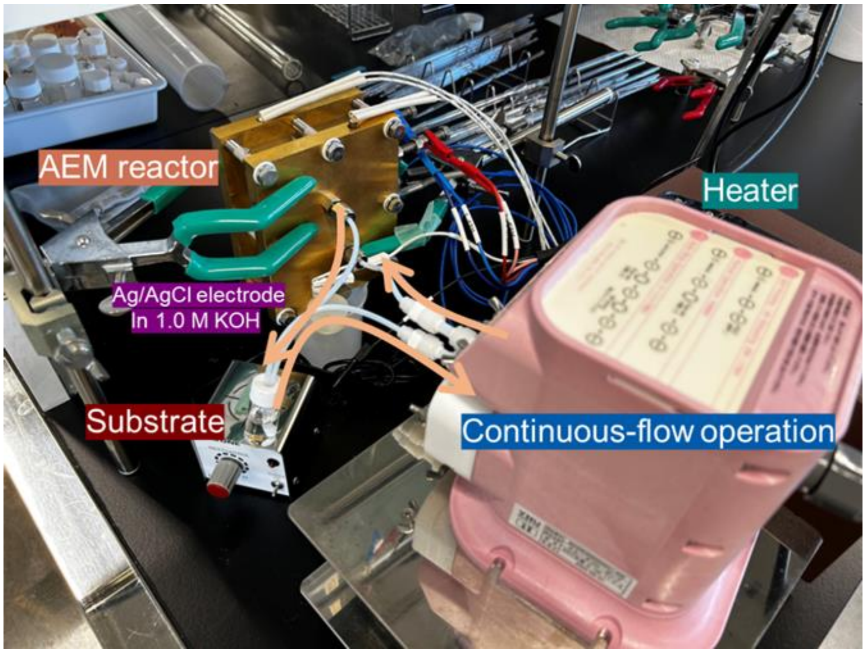

Figure S2 Photograph during the electrosynthesis with the setup using water as solvent.

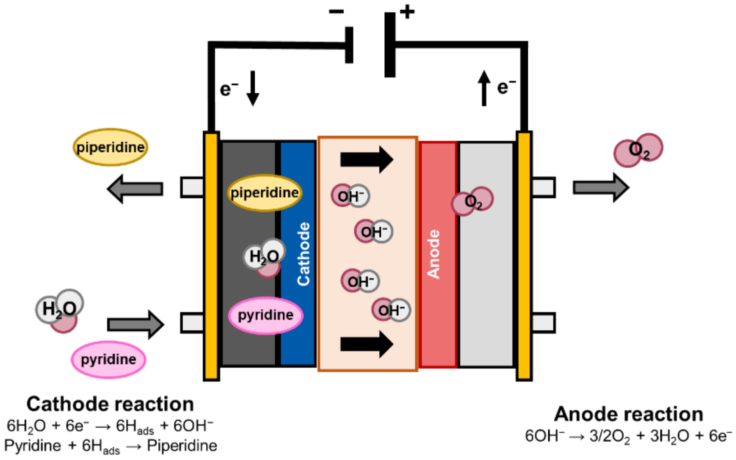

Figure S3 Overall reaction using water as solvent.

5. Constant-current electrolysis in an AEM reactor with a non-aqueous solution for catholyte and aqueous solution for anolyte

Prior to the electrolysis using substrate, pre-electrolysis was performed for 2 hours at 50 mA cm^{-2} with MTBE on the cathode side and 10 mM KOH solution. Then, the reaction solution containing substrate was introduced and circulated to replace the catholyte solution left in pre-electrolysis. The flow rates of the cathode and anode solutions were set to 2.0 mL min^{-1}.

The summary of the overall reaction for a non-aqueous solvent for the cathode is described in Figure S5.

For the catalyst screening experiment shown in Figure 2h in the manuscript, 3 mL of 50 mM pyridine (**1a**) solution dissolved in methyl *tert*-butyl ether (MTBE) was circulated to the cathode side, and 10 mM KOH solution was circulated to the anode side at a flow rate of 2.0 mL min^{-1} at 50 mA cm^{-2} for 36 min (30 F mol^{-1}) (Figure S4). After electrolysis, the samples were analyzed directly by GC. The reaction mechanism for a non-aqueous solvent for the cathode is shown in Figure S5.

For the substrate screening experiment shown in Table 1 in the manuscript, 5 mL of 100 mM (**1k**, **1l**) or 50 mM (**1m-p**) solution in MTBE was circulated to the cathode side, and 10 mM KOH solution was circulated to the anode side at a flow rate of 120 mL h^{-1} at 25 mA cm^{-2} for desired time (Figure S4). The reactions were all performed at 25 ºC except **2l** (50 ºC). After electrolysis, the collected samples were analyzed by GC (diglyme as an internal standard) or HPLC (dimethyl sulfone or benzaldehyde as an internal standard).

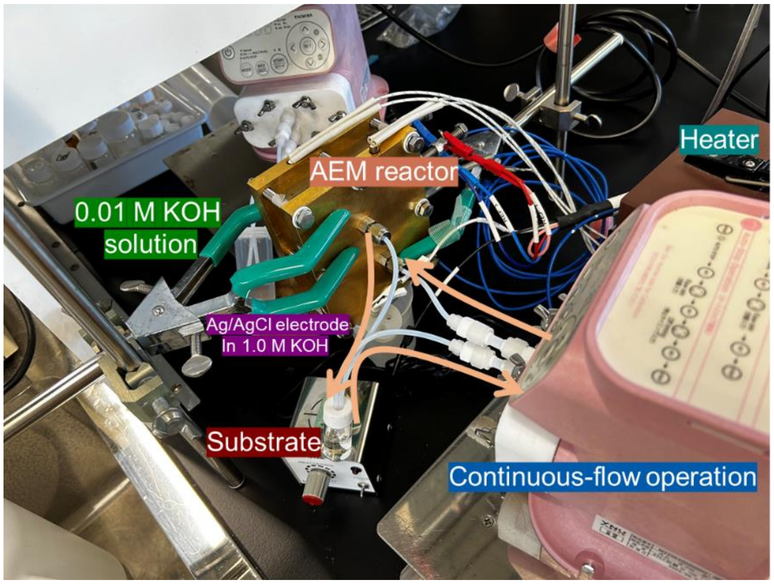

Figure S4 Photograph during the electrosynthesis with the setup using organic solvent.

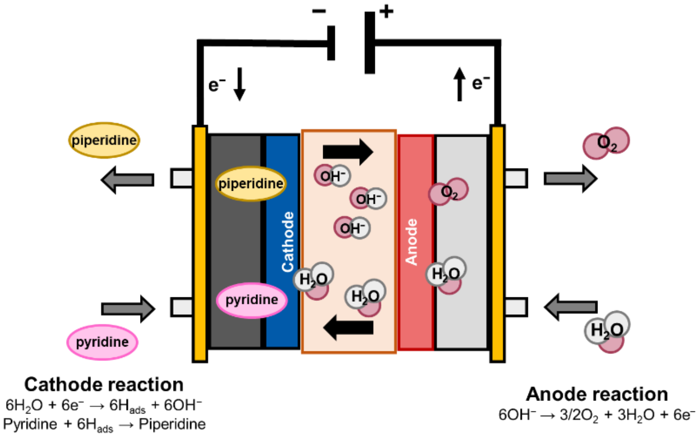

Figure S5 Overall reaction using organic solvent.

6. Comparison of acid-free hydrogenation of pyridine using heterogeneous catalysts

Several reports exist on the acid-free heterogeneous hydrogenation of pyridine to piperidine by thermal catalysis. Table S1 summarizes the nature of the catalyst, experimental conditions, yield, and reaction time for a comparison of the electrochemical system reported herein. We only summarized the data for pyridine-to-piperidine for direct and fair comparison. In other words, reports on functionalized pyridines or reactions using acid or other additives are not included in Table S1.

Table S1. Comparison of current system with other thermal hydrogenation systems of pyridine using heterogeneous catalysts

Entry	Catalyst	Solvent	H_2 Pressure [atm]	Temperature [C˚]	Yield [%]	Reaction time [h/mmol]	Reference
1	Co/Melamine-6@ TiO_2-800-5 (80 mg)	H_2O	60	160	96	96	1
2	Co-pyromellitic acid@SiO_2 (60 mg)	^{i}PrOH:H_2O	50	135	94	96	2
3	Ru/CN-SBA-15 (40 mg)	H_2O	20	90	quant.	4	3
4	NHC-stabilized Rh NPs (1.25 mol%)	THF	20	30	100	0.81	4
**5**	**Rh/C (28.9wt%, 2 mg)**	**H_2O**	**- (H_2O)**	**RT**	**99**	**2.4**	**This work**

7. Voltammetry measurements

Cyclic voltammetry (CV) was performed using AEM reactor and PEM reactor, respectively. The cathode and anode were Rh/KB (loading amount: 0.5 mg cm^{-2}) and DSE® electrode, respectively. A 50 mM solution of pyridine in MTBE was supplied to the cathodic chamber at a flow rate of 2.0 mL min^{-1}. A 10 mM KOH solution was supplied to the anodic chamber at a flow rate of 2.0 mL min^{-1} using an AEM electrolyzer. Humidified H_2 was supplied to the anodic chamber at a flow rate of 50 mL min^{-1} using a PEM electrolyzer. The cell temperature was set to room temperature. CV measurements were conducted at a scan rate of 10 mV s^{-1}.

8. Analysis of Rh/KB catalyst

Figure S6 shows the powder X-ray diffraction (XRD) pattern of Rh/KB before electrolysis; the XRD pattern suggests that the catalyst is mainly composed of rhodium metal. Figure S7 shows the X-ray photoelectron spectroscopy (XPS) data of Rh/KB before and after electrolysis. The XPS spectrum before electrolysis indicated the presence of Rh(III) species as well as Rh(0) (Figure 7a); the XRD and XPS data suggested that Rh/KB were metallic rhodium nanoparticles with an oxide layer on the surface. XPS spectra of the samples after electrolysis showed the disappearance of Rh(III) species, suggesting that the oxidized surface layer is reduced to metallic rhodium under the operating conditions (Figure S7b).

Transmission electron microscopy (TEM) was used to microscopically observe the Rh/KB catalyst (Figure S8). TEM images of the samples before and after electrolysis (for 318 hours) showed no essential difference, with the particle size remaining at about 2 nm.

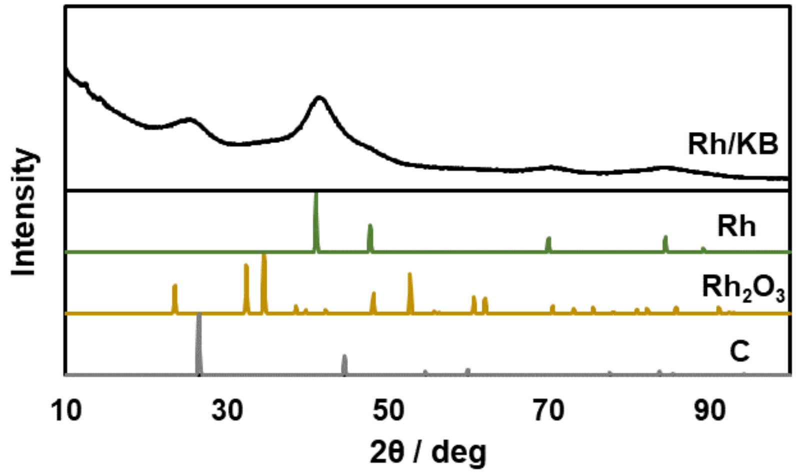

Figure S6 XRD data for Rh/KB catalyst

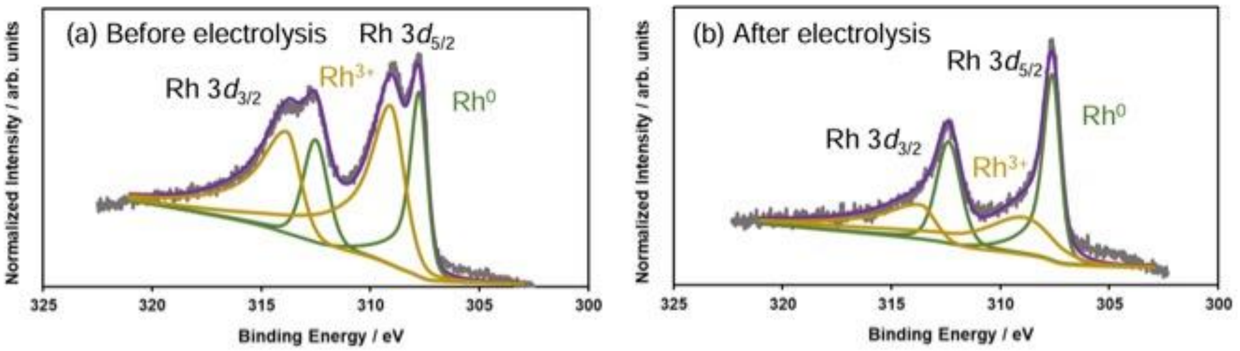

Figure S7 XPS spectra of Rh3*d* region for Rh/KB catalyst before (a) and after (b) the electrolysis.

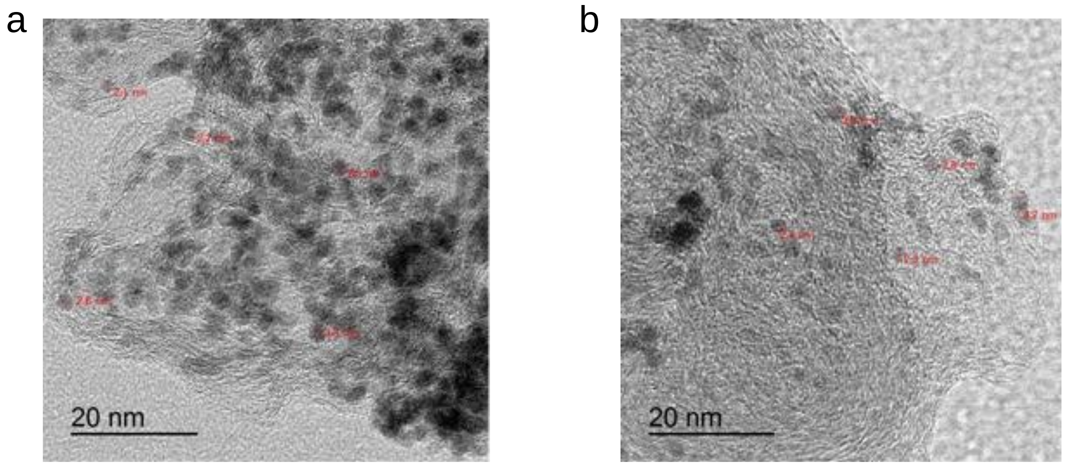

Figure S8 TEM image of Rh/KB catalyst before (a) and after (b) the large-scale electrolysis.

9. In situ XAFS measurement

Rh K-edge X-ray absorption fine structure (XAFS) measurements were conducted using the BL14B2 beamline at the SPring-8 synchrotron radiation facility (Hyogo, Japan). The spectra were recorded in a transmission mode at room temperature using a Si (311) (24.3473 keV, θ = 8.74) double-crystal monochromator. The X-ray energy was calibrated using a peak top in the first derivative spectrum of Rh foil (E_0 = 23224.508 eV). Data reduction was performed using Athena from the Demeter software package.[5] Linear combination fitting (LCF) analysis was performed for the normalized X-ray absorption near edge structure (XANES) spectra by using Rh foil, Rh_2O_3 as references.

The in situ XAFS measurements were performed according to the sequence shown in Figure S9. After setting the AEM electrolyzer equipped with MEA to the laser line (Figure S10), deionized water was introduced to the cathodic chamber by syringe pump. After introducing enough volume of water, electrolysis was performed at 25 mA cm^{-2} for 640 s. During this electrolysis, XANES spectra were collected.

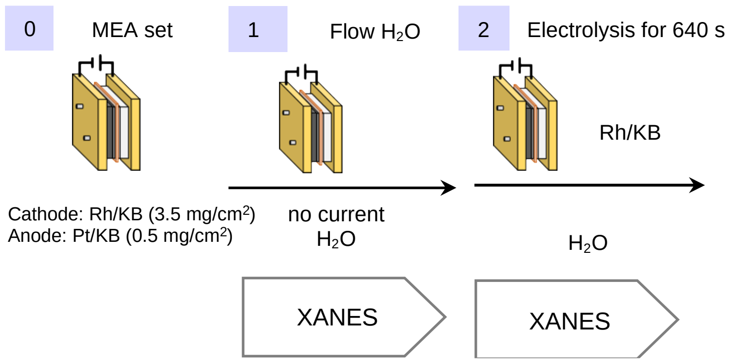

Figure S9 An experimental sequence of the in situ XAFS measurement

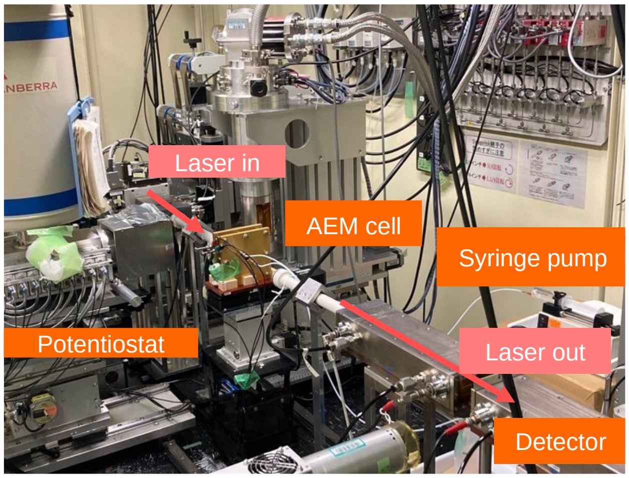

Figure S10 Photograph of experimental setup for in situ XAFS measurement.

10. Thermodynamic calculation of direct electrochemical hydrogenation

The half reaction and net reaction schemes for the direct electrochemical hydrogenation of **1a** follows as Eq. S1–S3:

$$\boldsymbol{Anode}: 6\mathrm{OH}^- \rightarrow \frac{3}{2}\mathrm{O}_2 + 3\mathrm{H}_2\mathrm{O} + 6\mathrm{e}^- \quad (S1)$$

$$\boldsymbol{Cathode}: \mathbf{1a} + 6\mathrm{H}_2\mathrm{O} + 6\mathrm{e}^- \rightarrow \mathbf{2a} + 6\mathrm{OH}^- \quad (S2)$$

$$\boldsymbol{Net\ reaction}: \mathbf{1a} + 3\mathrm{H}_2\mathrm{O} \rightarrow \mathbf{2a} + 3/2\,\mathrm{O}_2 \quad (S3)$$

The theoretical voltage of this reaction (Eq. S3) can be calculated from the Gibbs free energy.[6] The relationship between the standard electrode potential E° and the Gibbs free energy ΔG° is

$$E^\circ = -\frac{\Delta G^\circ}{nF} \quad (S4)$$

where n is the number of moles of electrons and F is the Faraday constant.

To calculate $\Delta_f S^\circ$, the entropies of the elements in their standard states are needed, along with the entropies of the respective compounds:

$$\Delta_f S^\circ = S^\circ{}_{\mathrm{prodcut}} - S^\circ{}_{\mathrm{elements}} \quad (S5)$$

Solving Eq. S5 for $\Delta_f S^\circ$ for **1a** and inserting the values for $S^\circ{}_{\mathbf{1a}}$ and $S^\circ{}_{\mathrm{elements}}$, we obtain

$$\Delta_f S^\circ{}_{\mathbf{1a}} = S^\circ{}_{\mathbf{1a}} - S^\circ{}_{\mathrm{elements}}$$

$$= 180.75(\mathrm{J/mol\cdot K}) - \left(5 \times S^\circ{}_{Carbon} + \frac{5}{2} \times S^\circ{}_{H_2} + \frac{1}{2} \times S^\circ{}_{N_2}\right)$$

$$= -270.1275\ (\mathrm{J/mol\cdot K})$$

We can now calculate $\Delta_f G^\circ{}_{\mathbf{1a}}$ as follows.

$$\Delta_f G^\circ{}_{\mathbf{1a}} = \Delta_f H^\circ{}_{\mathbf{1a}} - T\Delta_f S^\circ{}_{\mathbf{1a}}$$

$$= 95.30\ \mathrm{kJ/mol} - [298.15\ (K) \times (-270.1275\ (\mathrm{J/mol\cdot K}) \times 10^{-3}]$$

$$= 175.84\ (\mathrm{kJ/mol})$$

Also, we can calculate for $\Delta_f S^\circ$ and $\Delta_f G^\circ{}_{\mathbf{2a}}$ for **2a** as well.

$$\Delta_f S^\circ{}_{\mathbf{2a}} = S^\circ{}_{\mathbf{2a}} - S^\circ{}_{\mathrm{elements}}$$

$$= 209.97\ (\mathrm{J/mol\cdot K}) - \left(5 \times S^\circ{}_{Carbon} + \frac{11}{2} \times S^\circ{}_{H_2} + \frac{1}{2} \times S^\circ{}_{N_2}\right)$$

$$= -632.6205\ (\mathrm{J/mol\cdot K})$$

$$\Delta_f G^\circ{}_{\mathbf{2a}} = \Delta_f H^\circ{}_{\mathbf{2a}} - T\Delta_f S^\circ{}_{\mathbf{2a}}$$

$$= -88.38\ (\mathrm{kJ/mol}) - [298.15\ (K) \times (-632.6205\ (\mathrm{J/mol\cdot K}) \times 10^{-3}]$$

$$= 100.2358\ (\mathrm{kJ/mol})$$

We can then calculate E° for the cathode reaction Eq. S2 using Eq. S4.

$$E^\circ_{\mathrm{cathode}} = -\frac{\Delta G^\circ}{6F}$$

$$= -\frac{\Delta_\mathrm{f} G^\circ_{\mathrm{product}} - \Delta_\mathrm{f} G^\circ_{\mathrm{reactant}}}{6F}$$

$$= -\frac{\Delta_\mathrm{f} G^\circ_{\mathbf{2a}} + 6 \times \Delta_\mathrm{f} G^\circ_{\mathrm{OH^-}} - (\Delta_\mathrm{f} G^\circ_{\mathbf{1a}} + 6 \times \Delta_\mathrm{f} G^\circ_{\mathrm{H_2O}})}{nF}$$

$$= -0.70\ \mathrm{V}\ vs.\ \mathrm{SHE}$$

The value of E° for the anode reaction (Eq. S1) is 0.40 V *vs.* SHE under pH 14. Therefore, the theoretical electrolysis voltage ΔE for the net reaction (Eq. S3) is

$$\Delta E^\circ = \left|E^\circ_{\mathrm{cathode}} - E^\circ_{\mathrm{anode}}\right|$$

$$= |-0.70\ (\mathrm{V}) - 0.40\ (\mathrm{V})|$$

$$= 1.10\ \mathrm{V}$$

However, ΔE for water splitting is 1.23 V. Therefore, direct electrochemical hydrogenation theoretically requires less energy than H_2-gas-based hydrogenation, including green H_2 obtained by water splitting.

11. Computational details

First-principles calculations were conducted by spin-polarized DFT methods using the Vienna Ab initio Simulation Program (VASP). We used The projector-augmented-wave pseudopotential (PAW) to describe the core electrons, whereas the generalized gradient approximation of the Perdew–Burke–Ernzerhof model (GGA-PBE), with D3 dispersion correction, was used to treat the exchange-correlation energy. The plane-wave cutoff energy was set to be 400 eV.

A vacuum region was added to the slab with a length greater than 15 Å along the *z*-axis to eliminate interactions between successive slabs. Rh(111) or Pt(111) facet models (4 × 4× 3) were used as the substrate. A 4 × 4 × 1 Monkhorst–Pack k-point mesh was used for the Brillouin zone integration, and the criterion for the maximum force for all moiety units was less than 0.02 eV/Å. The Blocked-Davidson and RMM–DIIS algorithms were employed in the self-consistent field (SCF) method.

The Gibbs free energy of each chemical state can be calculated by

$$G = E_{\mathrm{DFT}} + E_{\mathrm{ZPE}} + \int C_{\mathrm{p}} \mathrm{d}T - TS$$

where E_{DFT} and E_{ZPE} are the total energy obtained by DFT calculations and the zero-point energy, respectively, $\int C_{\mathrm{p}} \mathrm{d}T$ is the integrated form of the vibrational heat capacity, and TS is the entropy correction term (Table S2). The temperature was set to 298.15 K; the respective correction terms are shown in Table S2.

Following the report by Nørskov *et al.*, we applied the concept of computational hydrogen electrode (CHE) to establish a potential-dependent free energy diagram. The chemical potential of a proton and electron pair $G(H^+ + e^-)$ is equivalent to one-half of the chemical potential of gaseous H_2 [$0.5G(H_2)$] (0 V vs. the reversible hydrogen electrode (RHE)) and energy of $-eU$, where U is the external potential.

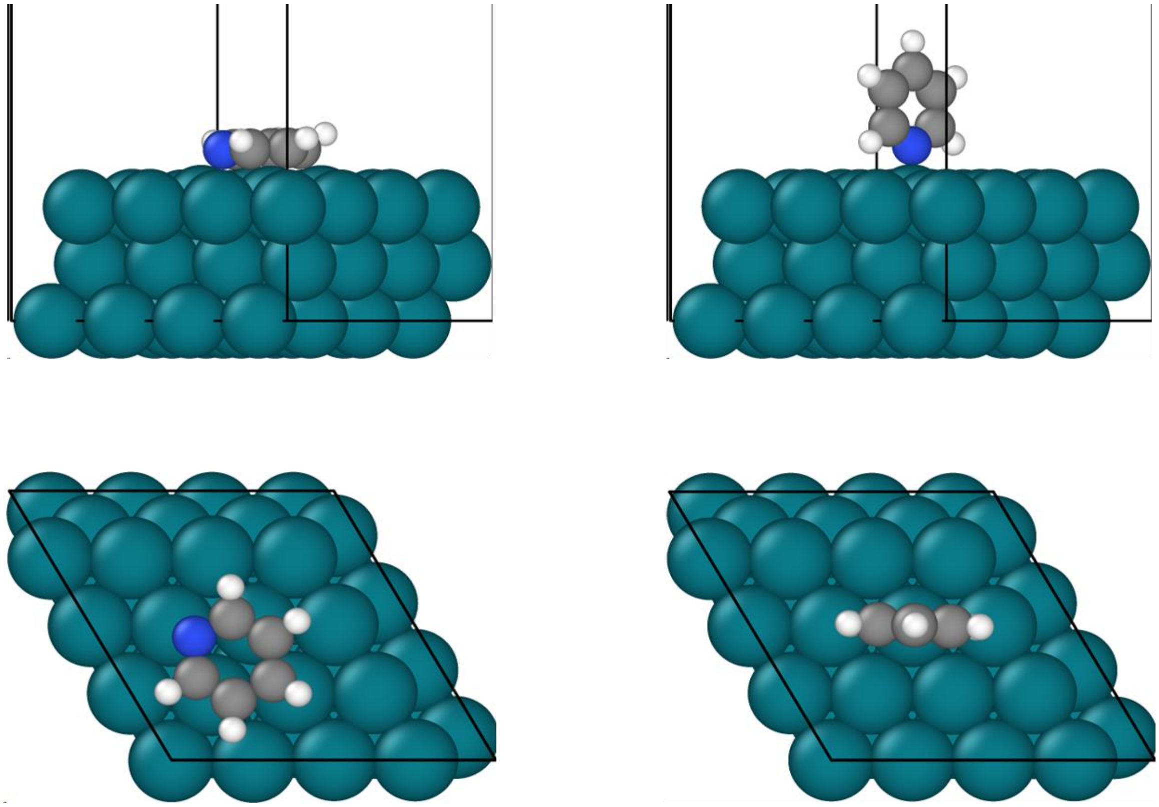

Figure S11 Top and side views of optimized structures of pyridine on Rh(111) with flat (left) and vertical (right) configurations

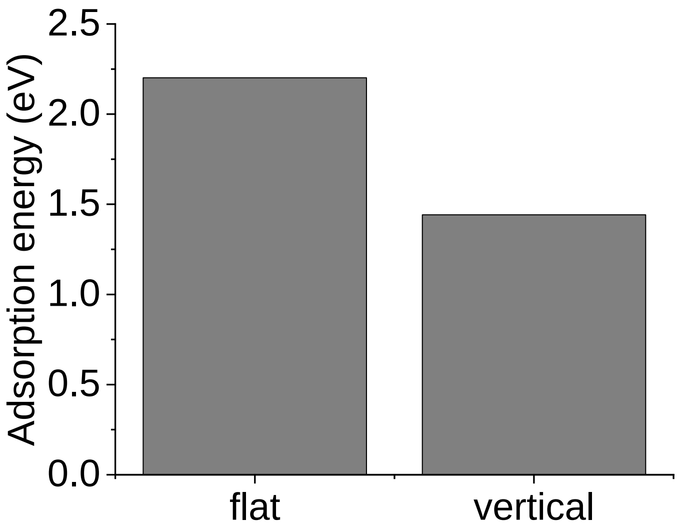

FigureS12 Adsorption energy of pyridine on Rh(111) with flat and vertical configurations

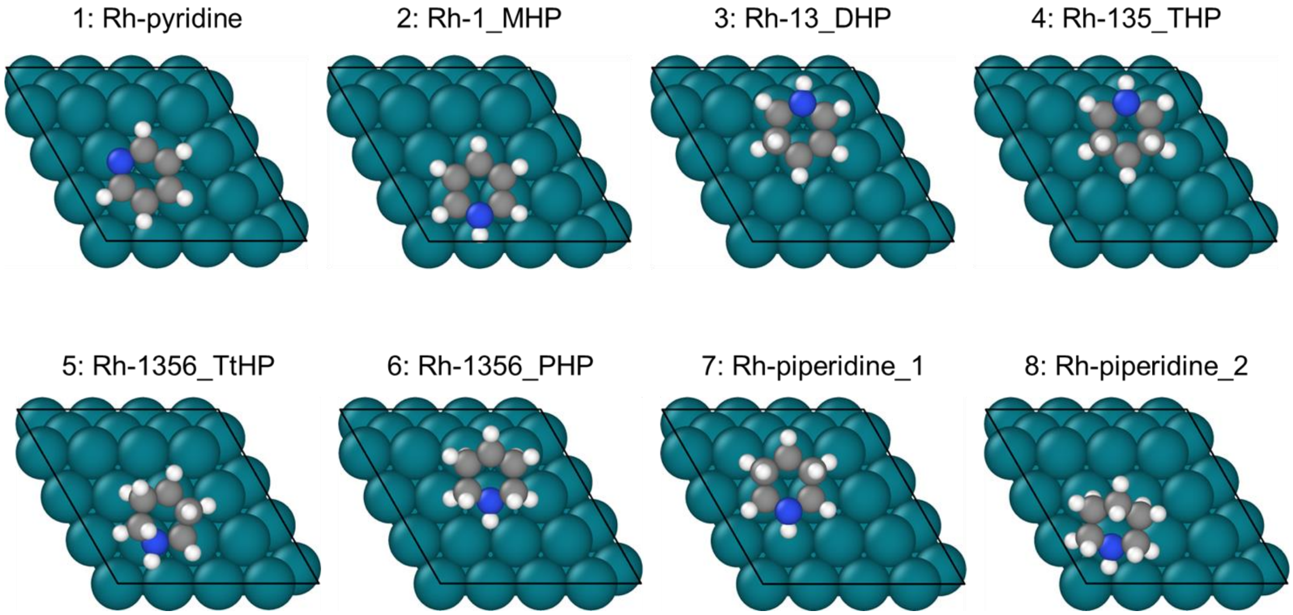

Figure S13 Top views of optimized structures of intermediates on Rh(111)

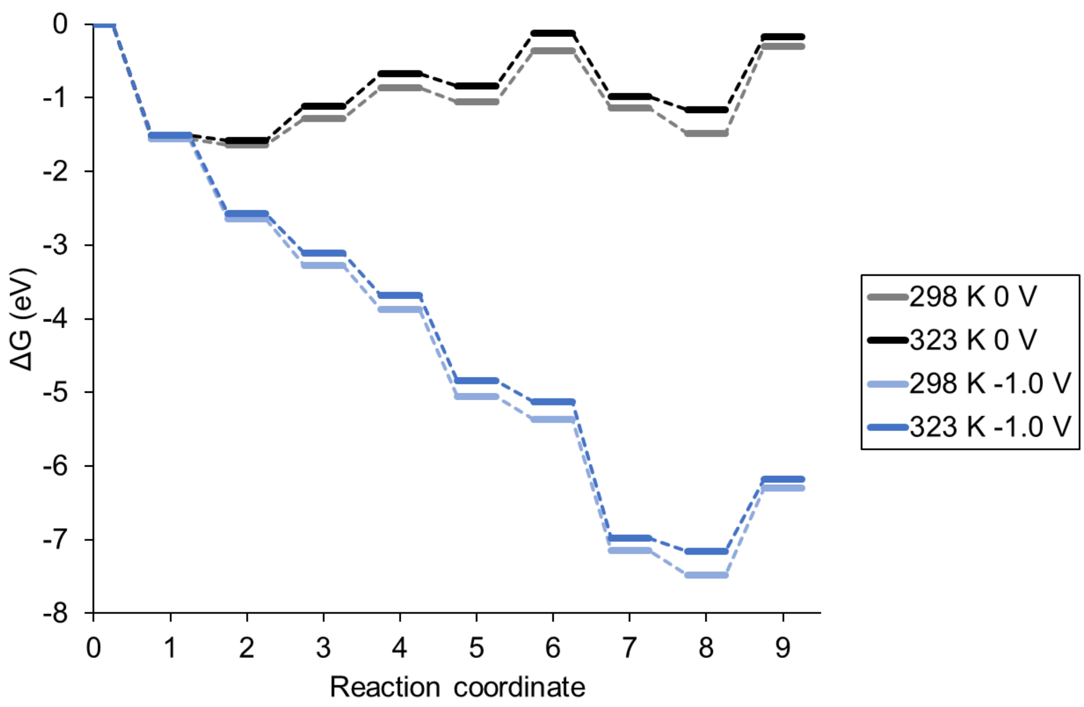

Figure S14 Free-energy diagrams of pyridine reduction reactions at 298 K and 323 K at 0 and -1.0 V vs. CHE

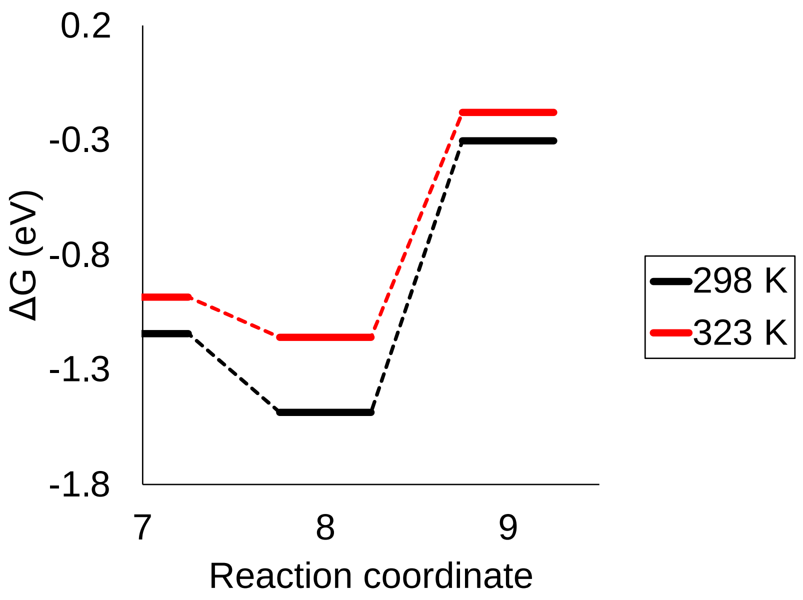

Figure S15 Energy diagrams of piperidine desorption at 298 K and 323 K

Table S2. The values of ZPE, Cp and TS on Rh(111)

Species	E_{ZPE}	$\int C_p dT$	TS	$G - E_{DFT}$
*pyridine	2.323	0.133	0.225	1.965
*1_MHP	2.638	0.15	0.264	2.224
*13_DHP	2.93	0.153	0.273	2.504
*135_THP	3.23	0.156	0.277	2.786
*1356_TtHP	3.572	0.156	0.277	3.139
*13562_PHP	3.708	0.18	0.342	3.186
*piperidine1	4.096	0.207	0.445	3.444
*piperidine2	4.205	0.194	0.405	3.606

12. Current efficiency and yield over time

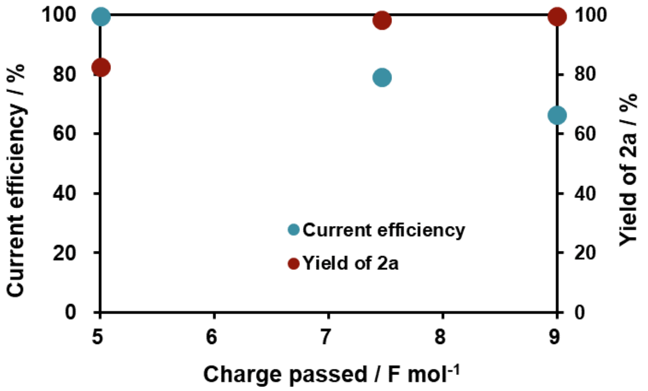

Figure S16 The plot of the current efficiency and yield of **2a** during electrocatalytic hydrogenation of **1a** by sampling gas chromatogram.

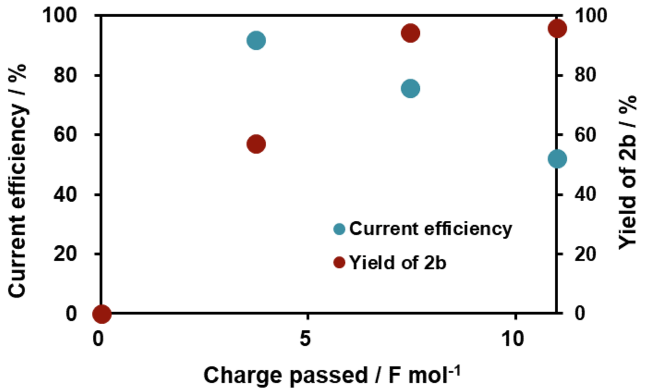

Figure S17 The plot of the current efficiency and yield of **2b** during electrocatalytic hydrogenation of **1b** by sampling gas chromatogram.

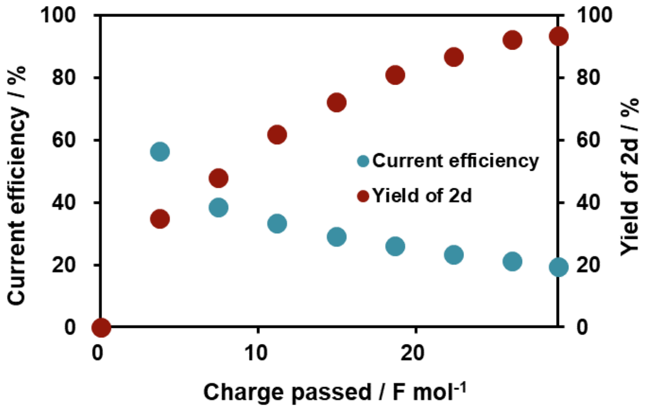

Figure S18 The plot of the current efficiency and yield of **2d** during electrocatalytic hydrogenation of **1d** by sampling gas chromatogram.

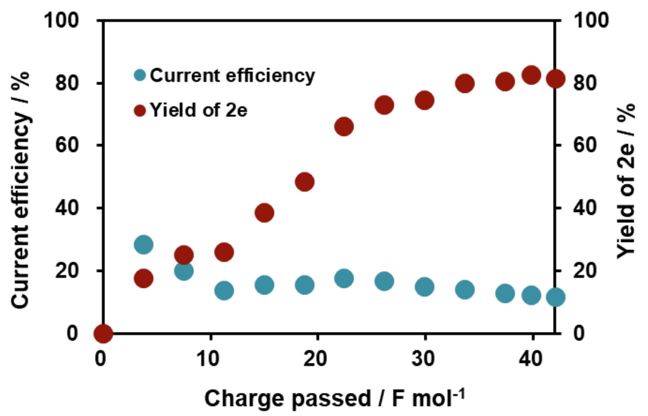

Figure S19 The plot of the current efficiency and yield of **2e** during electrocatalytic hydrogenation of **1e** by sampling gas chromatogram.

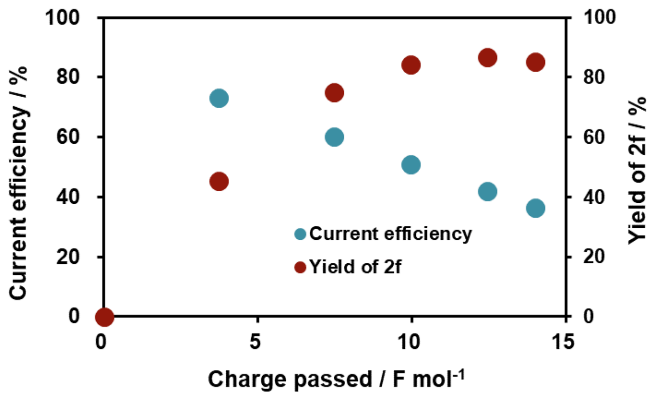

Figure S20 The plot of the current efficiency and yield of **2f** during electrocatalytic hydrogenation of **1f** by sampling gas chromatogram.

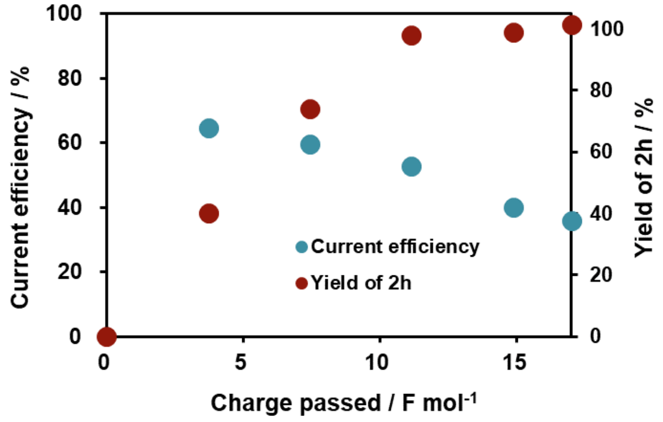

Figure S21 The plot of the current efficiency and yield of **2h** during electrocatalytic hydrogenation of **1h** by sampling gas chromatogram.

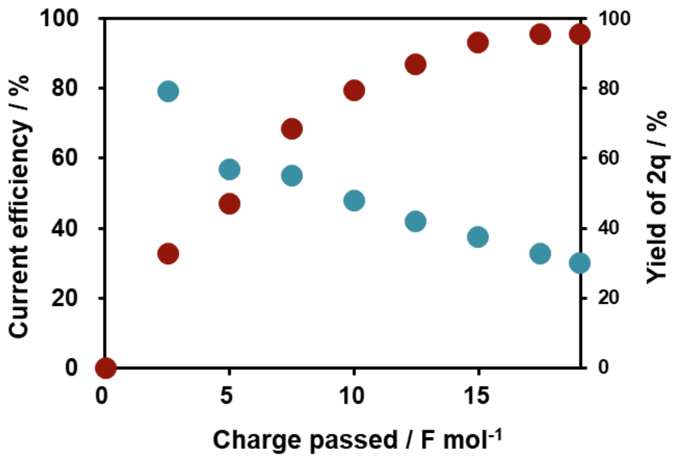

Figure S22 The plot of the current efficiency and yield of **2q** during electrocatalytic hydrogenation of **1q** by sampling gas chromatogram.

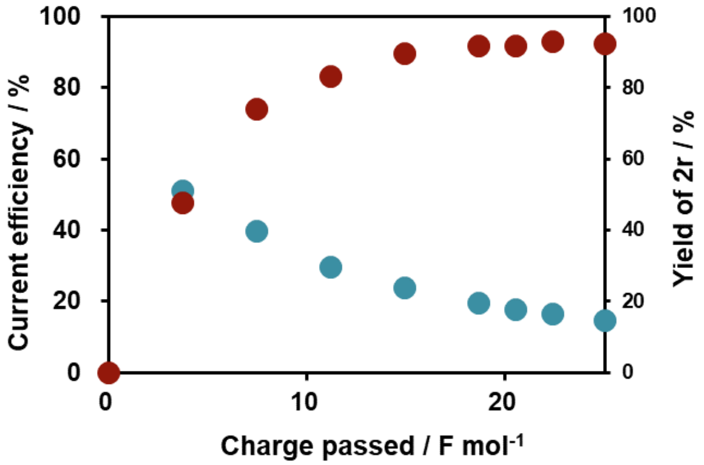

Figure S23 The plot of the current efficiency and yield of **2r** during electrocatalytic hydrogenation of **1r** by sampling gas chromatogram.

13. Hydrogenation of pyridine-d_5 using D_2O

To gain mechanistic insights, hydrogenation of pyridine-d_5 was performed using D_2O as a source of D^+ and electrons (Scheme S1). The electrolysis was performed under the identical conditions of **1a** to **2a** shown in Table 1 in the manuscript, where 100 mM pyridine-d_5 solution in D_2O was used as a catholyte. Constant current electrolysis at 25 mA cm^{-2} was performed until 9 F mol^{-1} of charge was passed. GC-MS analyzed the resulting solution. For comparison, a reaction mixture using **1a** was also prepared and subjected to GC-MS analysis. Authentic samples of **1a**, **2a**, pyridine-d_5, and piperidine-d_{11} were all subjected to GC-MS analysis, and mass spectra were compared (Figure S25-30). GC-MS analysis of the reaction mixture using pyridine-d_5 showed a chromatography signal from 6 min. The mass spectrum of that compound was close to an authentic sample of piperidine-d11. This experiment demonstrates that water is a source of hydrogen atoms in our electrocatalytic hydrogenation system.

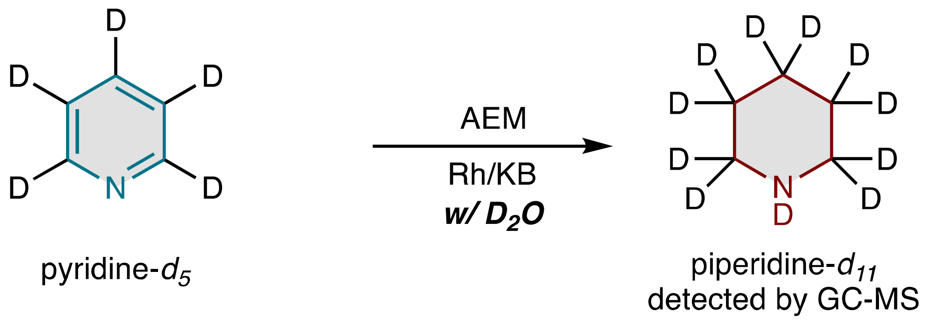

Scheme S1 Electrocatalytic hydrogenation of pyridine-d_5 in D_2O

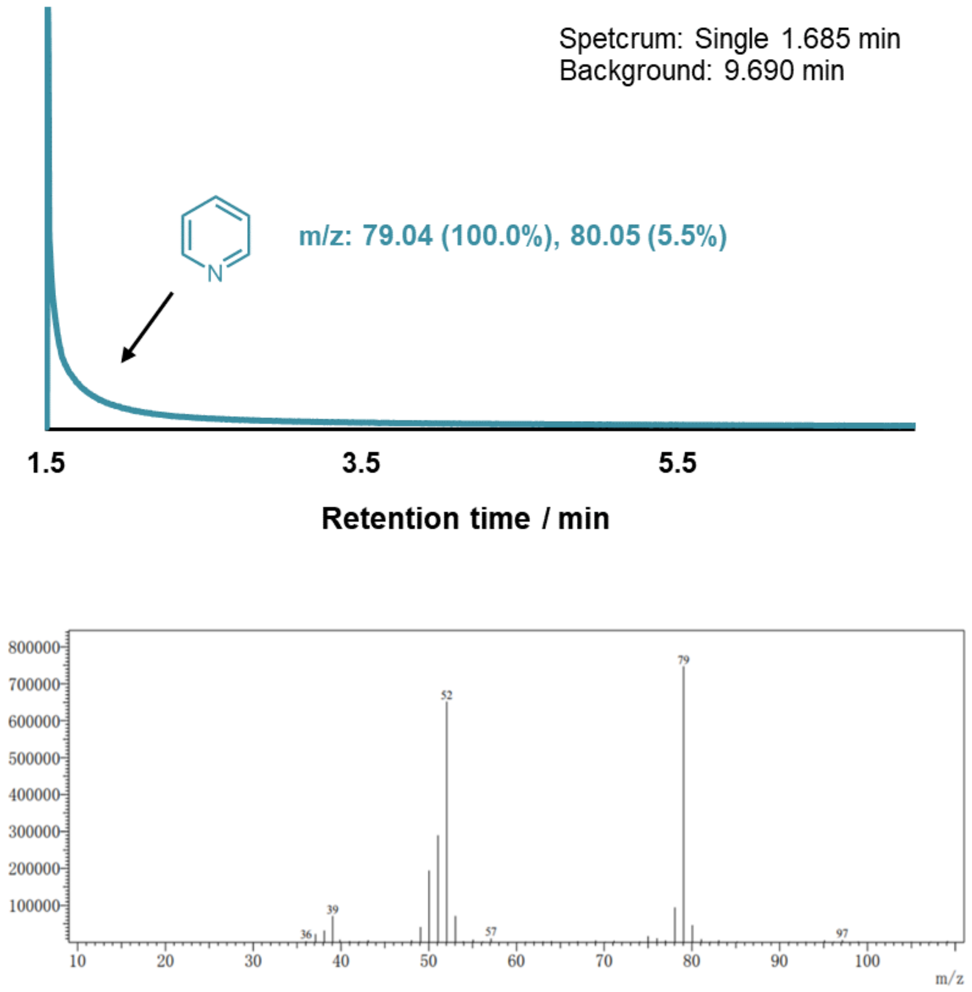

Figure S24 Gas chromatography mass spectrometry of the solution before electrocatalytic hydrogenation of **1a**.

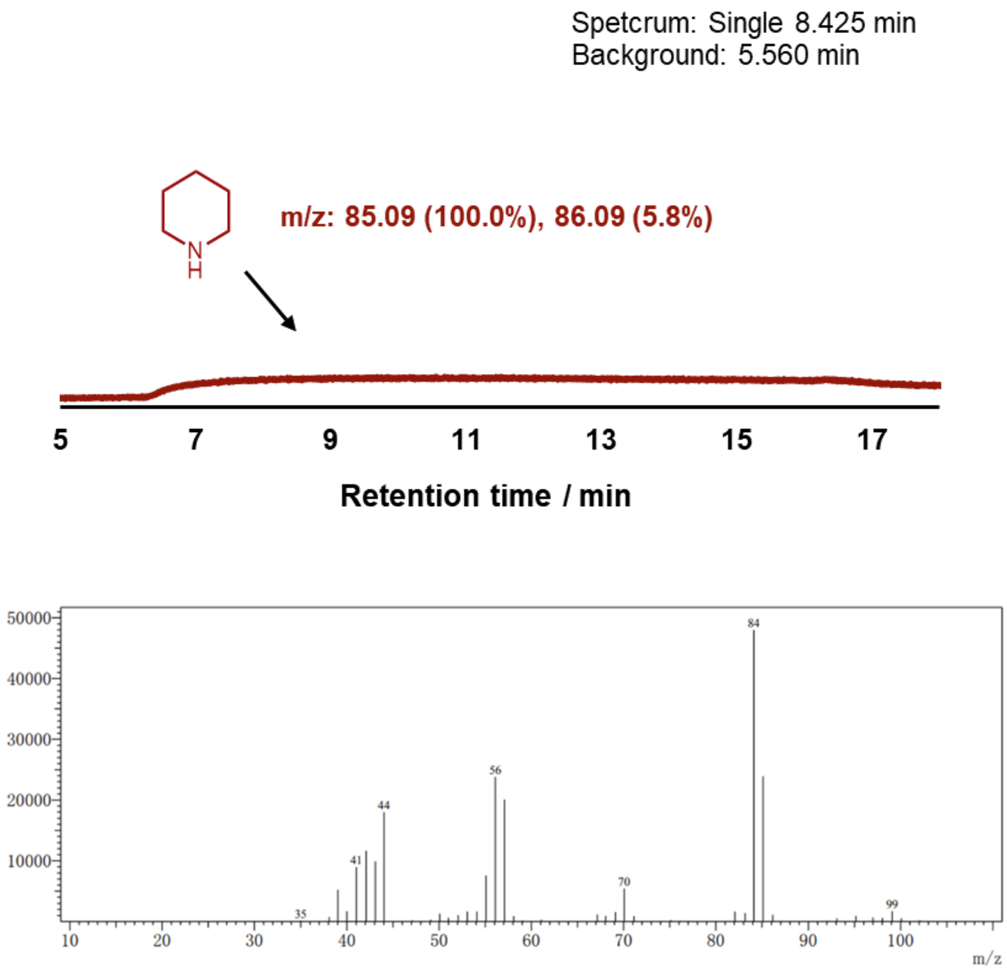

Figure S25 Gas chromatography mass spectrometry of the solution after electrocatalytic hydrogenation of **1a**.

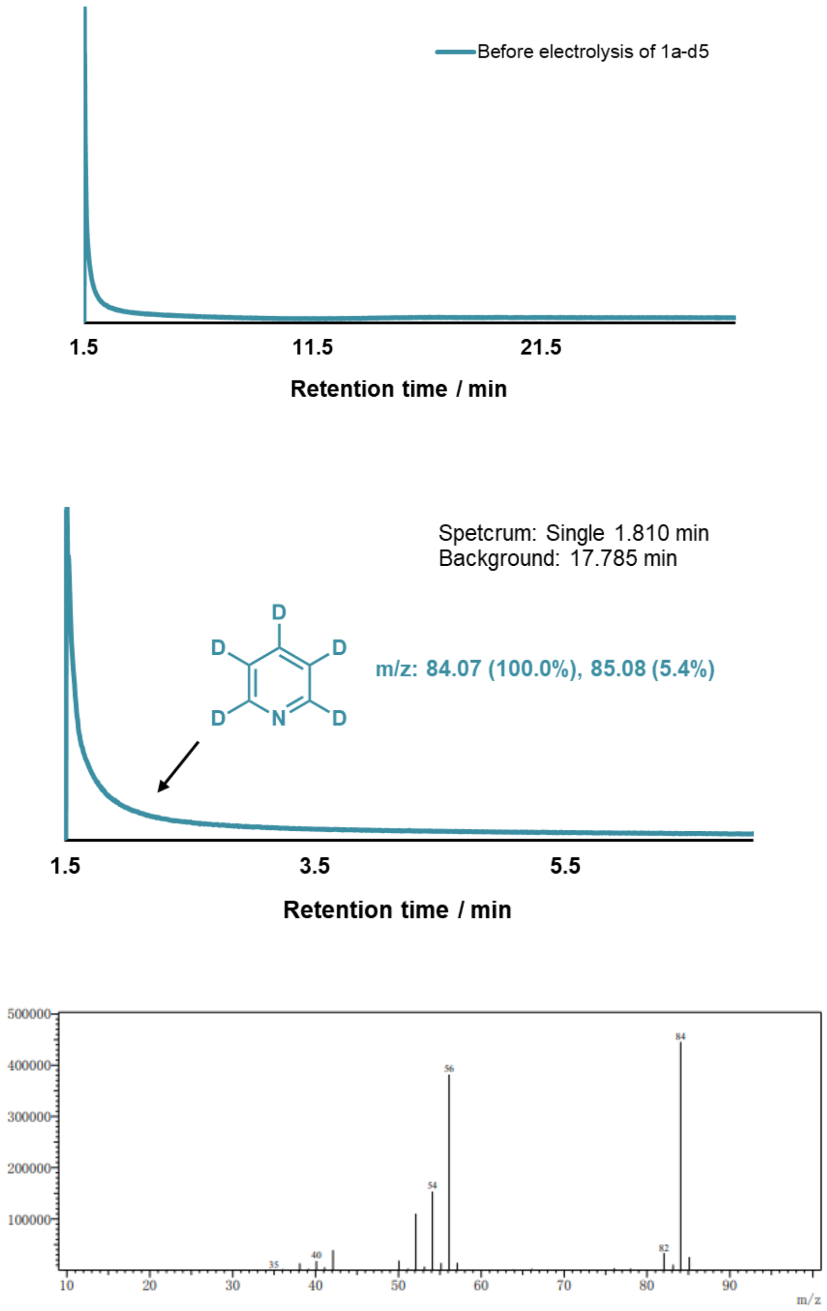

Figure S26 Gas chromatography mass spectrometry of the solution before electrocatalytic hydrogenation.

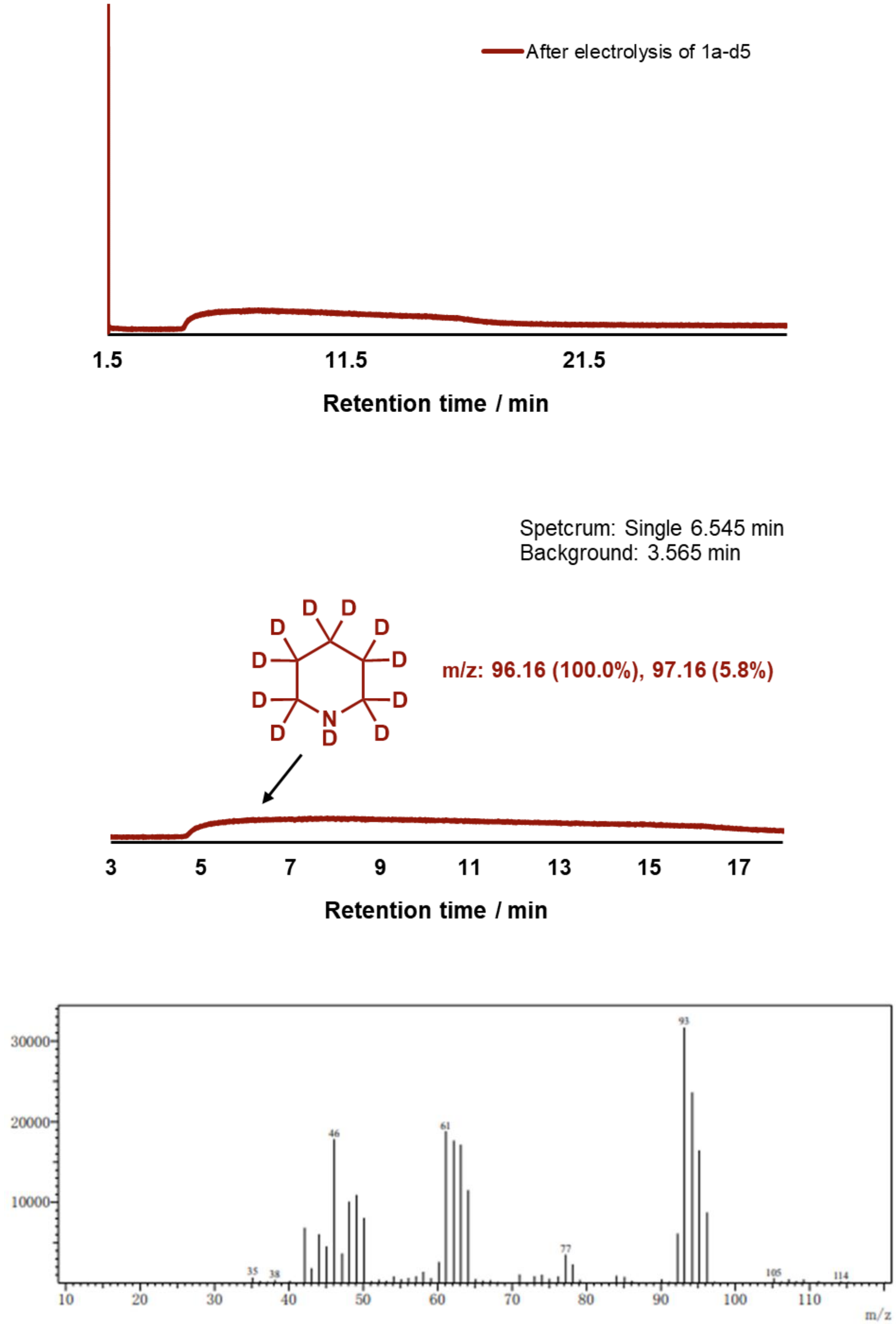

Figure S27 Gas chromatography mass spectrometry of the solution after electrocatalytic hydrogenation.

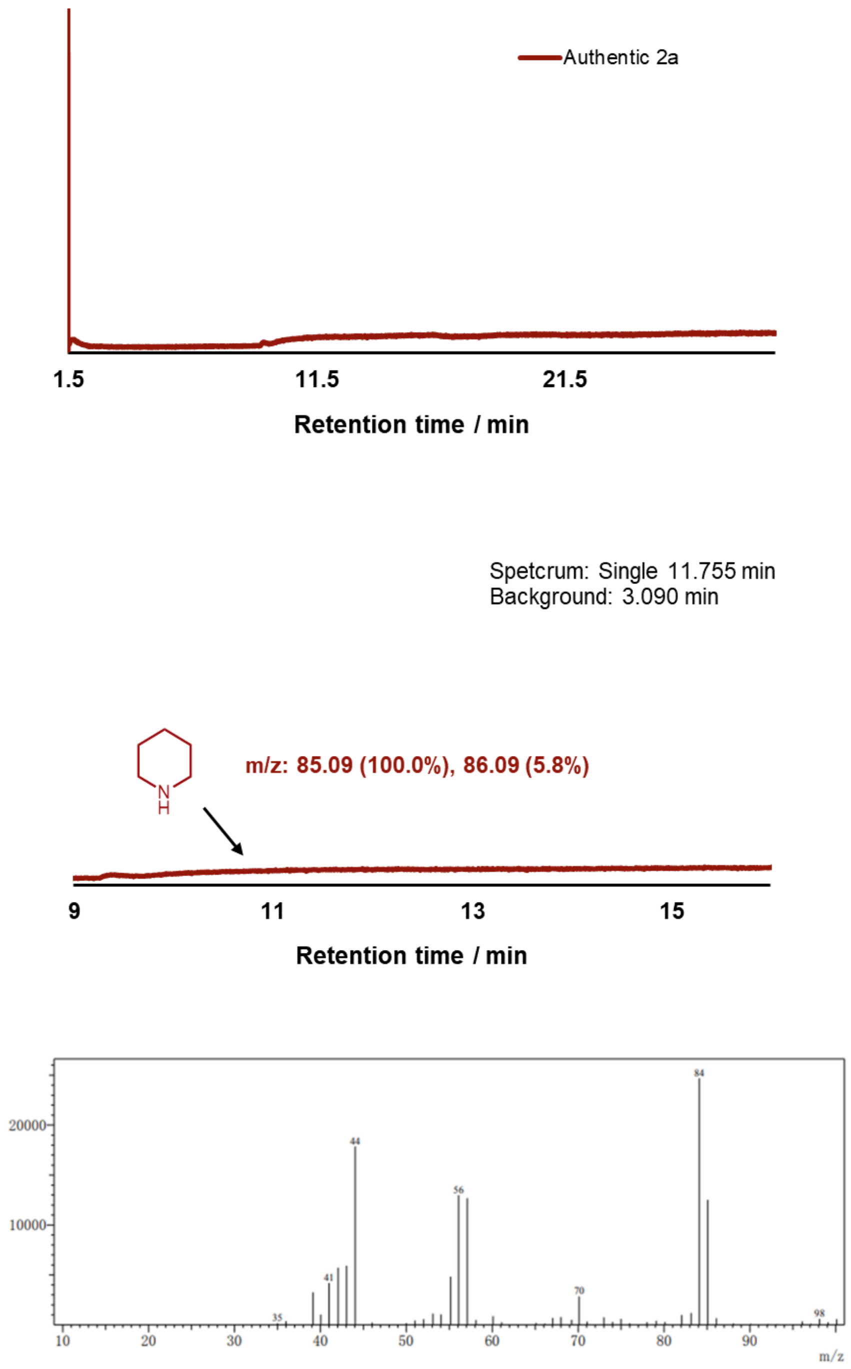

Figure S28 Gas chromatography mass spectrometry of authentic **2a**.

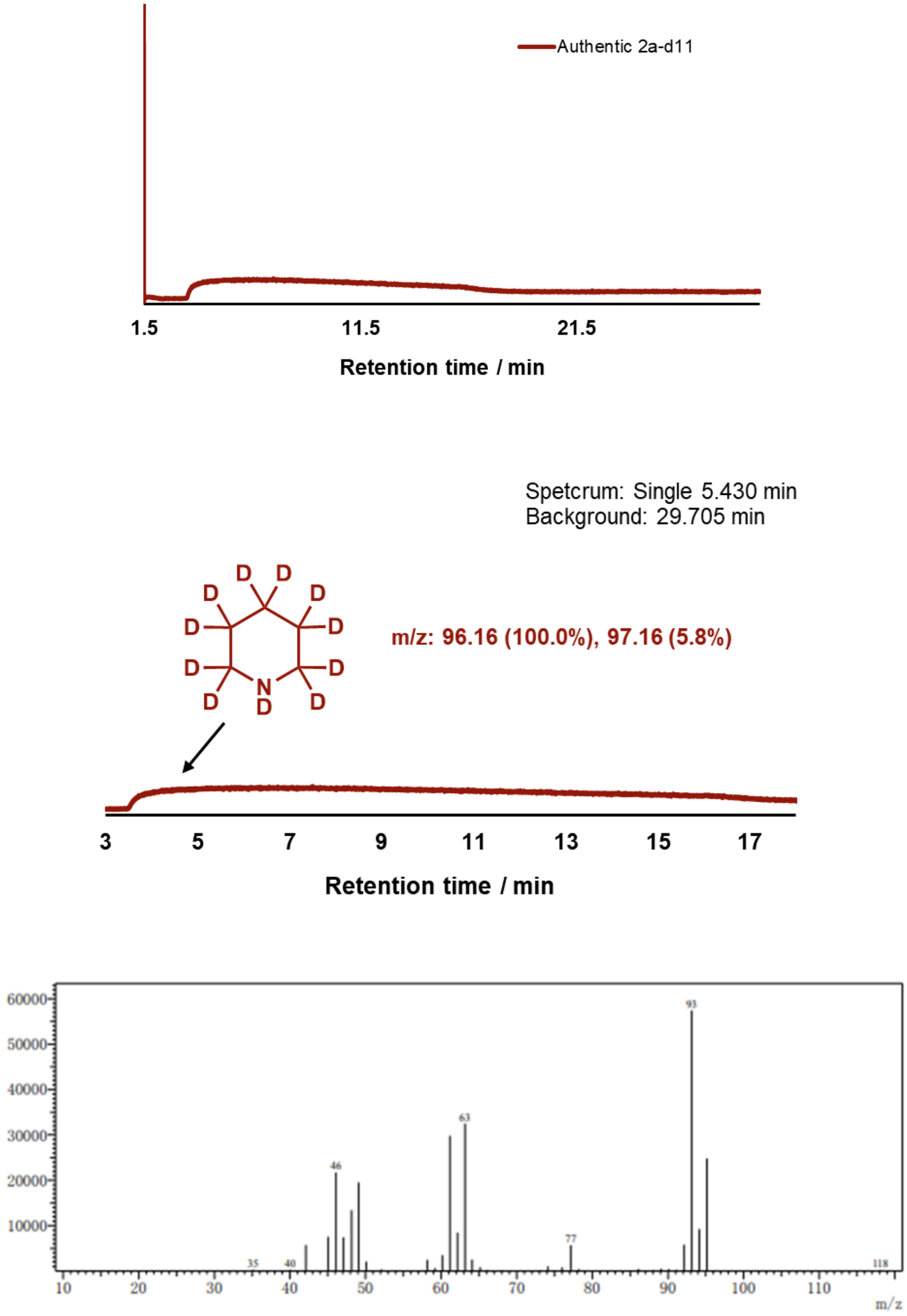

Figure S29 Gas chromatography mass spectrometry of authentic piperidine-d_{11}.

14. Additional data for substrate scope study

Electrocatalytic hydrogenations were also performed using 2-vinylpyridine, 2-ethynylpyridine, and indole, respectively, under the following reaction conditions (Figure S30).

***Condition A*:** catholyte, 5 mL solution of water/THF = 1/1 in vol; anolyte, air; current density, 25 mA cm^{-2}; anode, DSE anode; temperature, 25 °C. Determined by GC.

***Condition B*:** catholyte, 5 mL solution of MTBE; anolyte, 10 mM KOH; current density, 25 mA cm^{-2}; anode, DSE anode; temperature, 25 °C. Determined by GC.

For 2-vinylpyridine and 2-ethynylpyridine, hydrogenation of both aromatic ring and double or triple bond occurred simultaneously, and 2-ethylpyridine was obtained as the sole product in both cases. It is noteworthy that the current efficiency of 2-ethynylpyridine was much lower compared to 2-vinylpyridine or 2-ethylpyridine, presumably due to the weaker adsorption of the starting material. Electrochemical hydrogenation of indole scarcely proceeded under the conditions shown below. These experiments give deeper insights into the mechanism and limitations of the system.

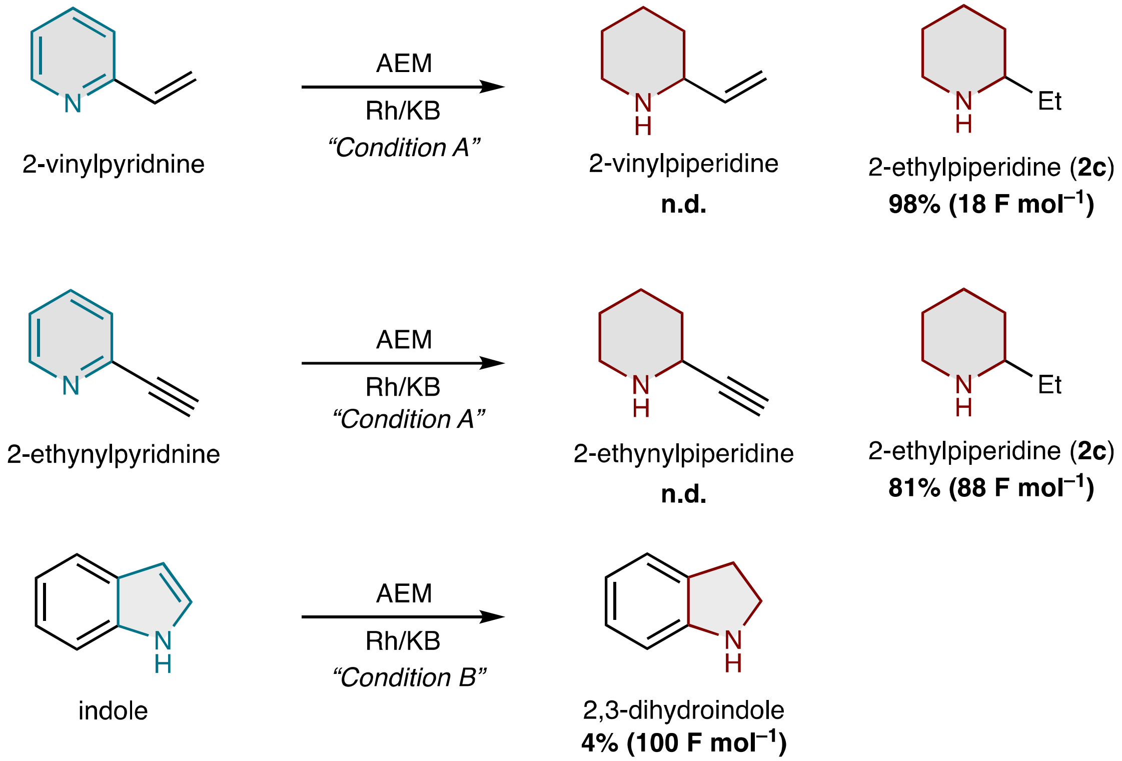

Figure S30 Additional substrate scope

15. Gas chromatograms

For GC analyses, samples were prepared by adding internal standard (diglyme) to the reaction solution, then diluted with solvent (methanol or MTBE, 4-fold dilution).

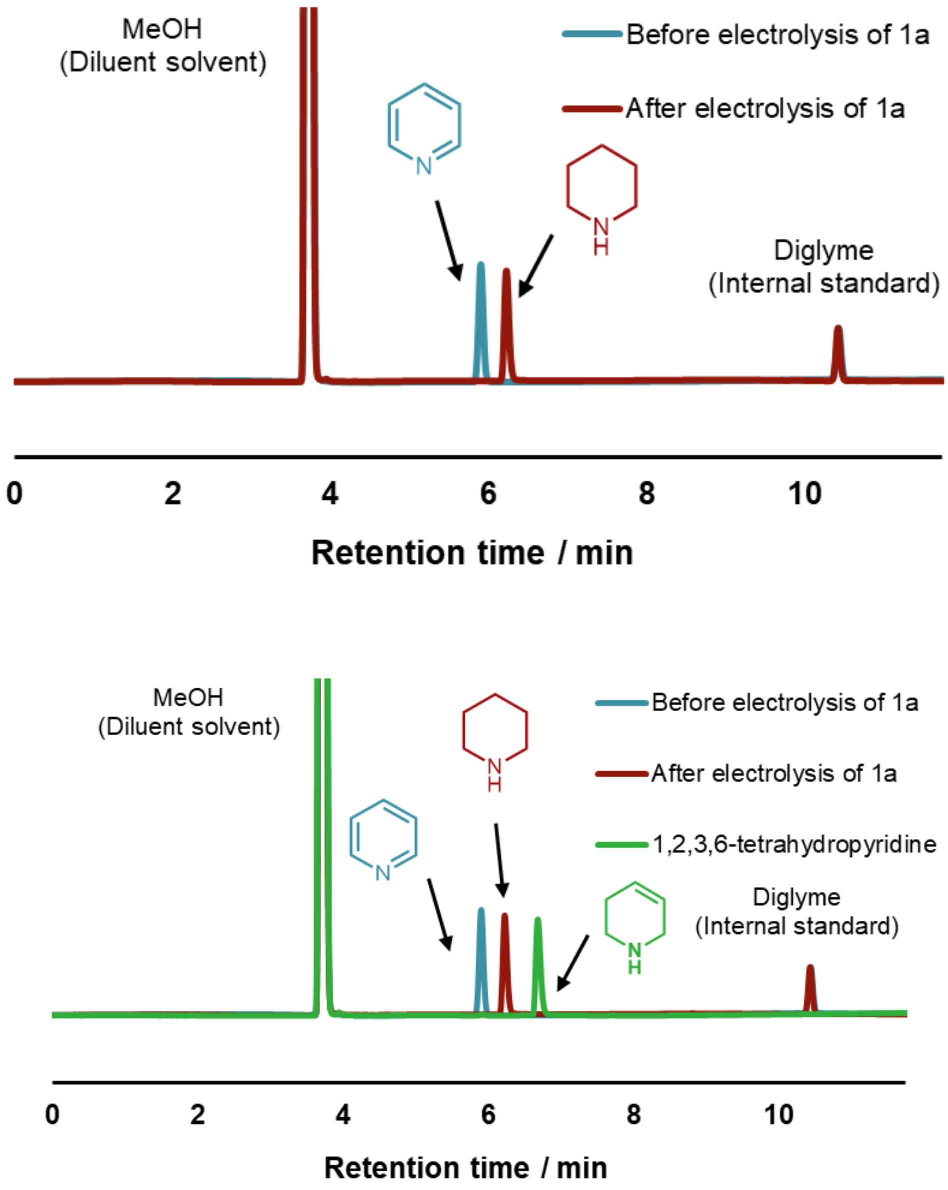

Figure S31 Gas chromatogram of the solution before electrocatalytic hydrogenation and reaction mixture obtained after electrocatalytic hydrogenation of **1a**. An authentic sample of 1,2,3,6-tetrahydoropyridine was also analyzed by GC, which was not detected in electrochemical hydrogenation at any time point of the reaction.

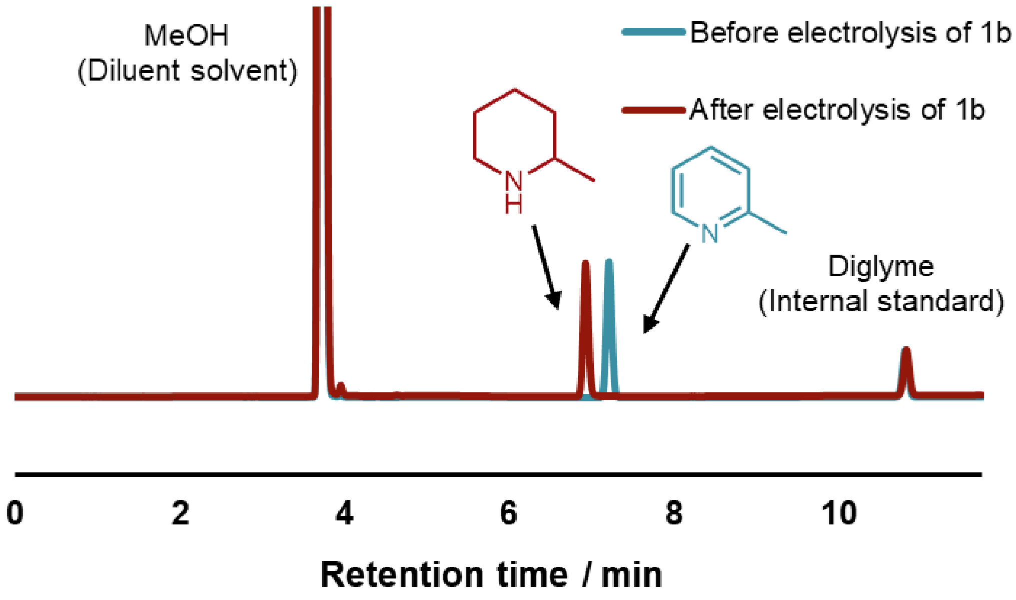

Figure S32 Gas chromatogram of the solution before electrocatalytic hydrogenation and reaction mixture obtained after electrocatalytic hydrogenation of **1b**.

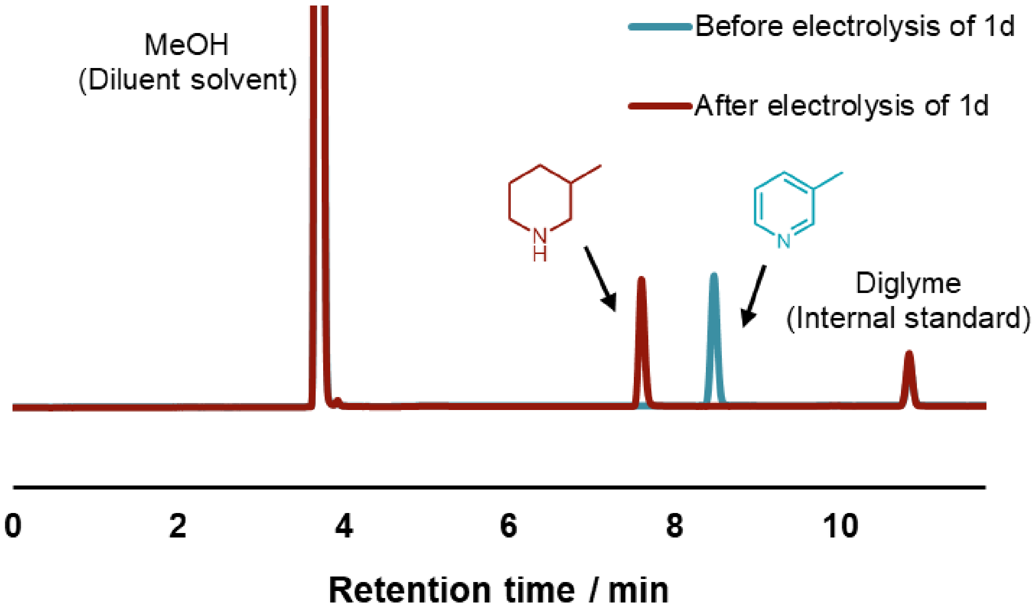

Figure S33 Gas chromatogram of the solution before electrocatalytic hydrogenation and reaction mixture obtained after electrocatalytic hydrogenation of **1d**.

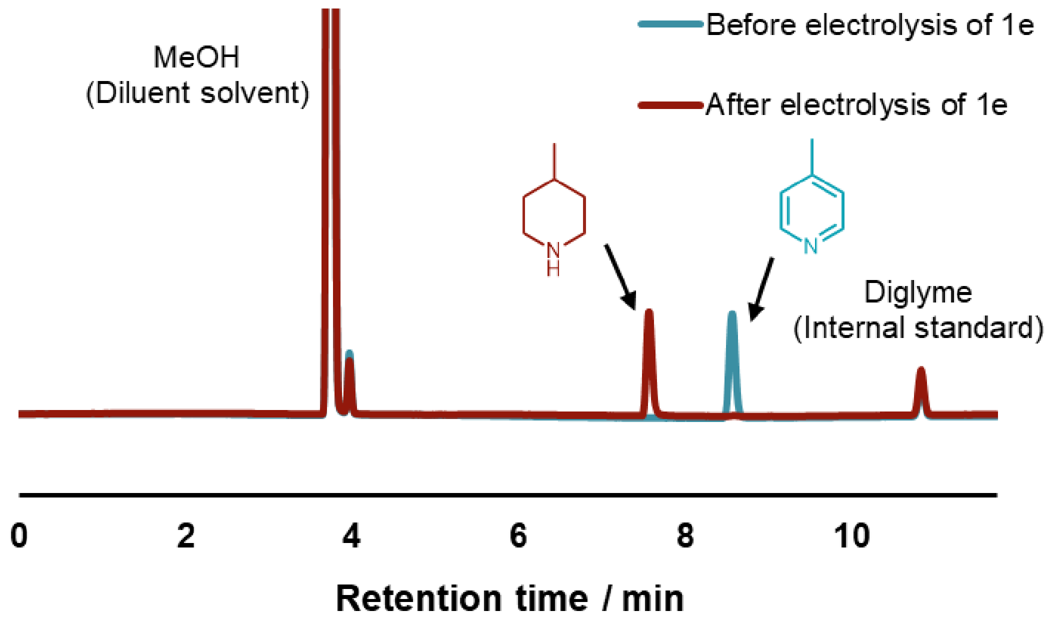

Figure S34 Gas chromatogram of the solution before electrocatalytic hydrogenation and reaction mixture obtained after electrocatalytic hydrogenation of **1e**.

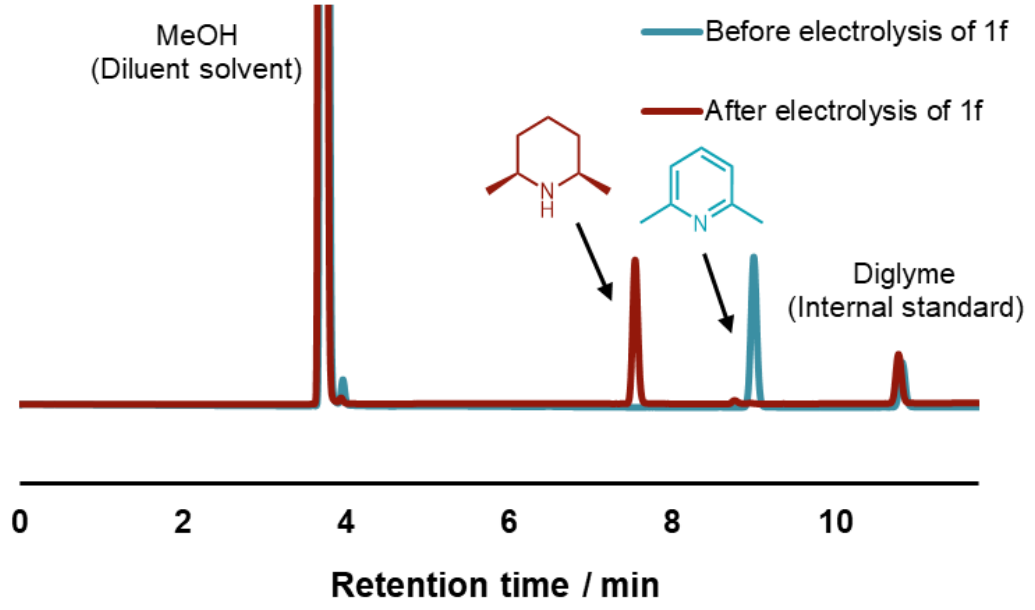

Figure S35 Gas chromatogram of the solution before electrocatalytic hydrogenation and reaction mixture obtained after electrocatalytic hydrogenation of **1f**.

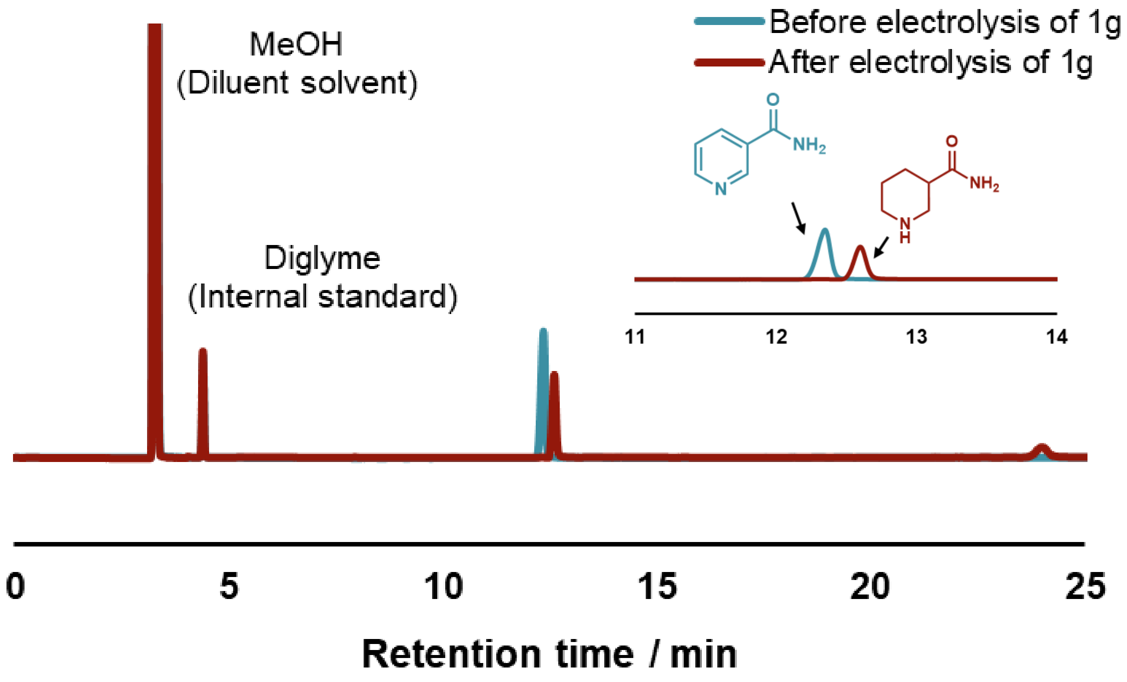

Figure S36 Gas chromatogram of the solution before electrocatalytic hydrogenation and reaction mixture obtained after electrocatalytic hydrogenation of **1g**.

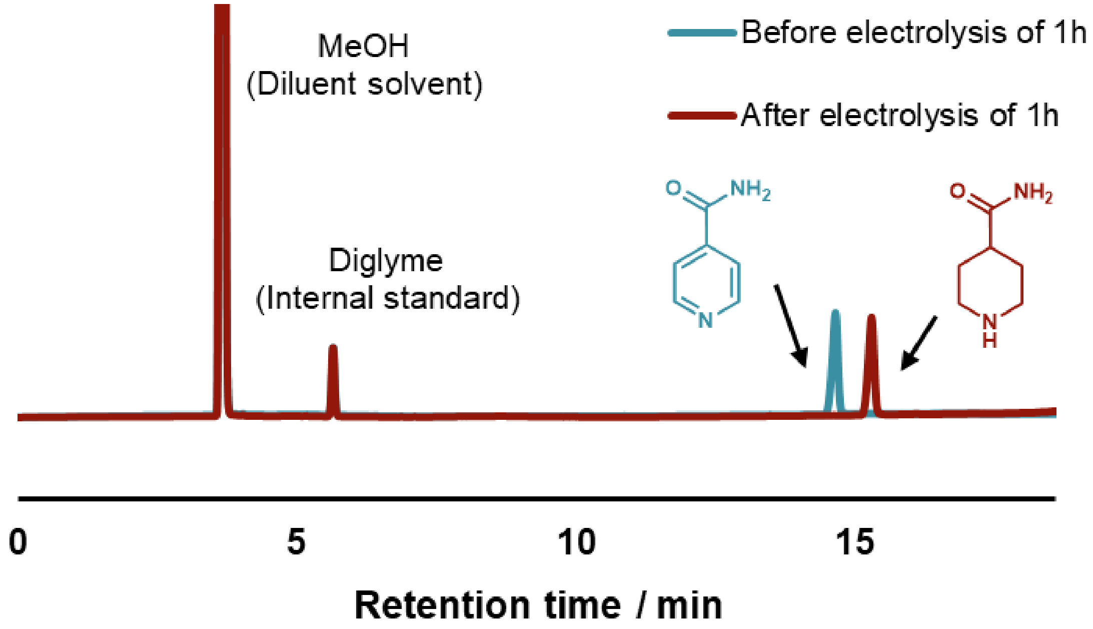

Figure S37 Gas chromatogram of the solution before electrocatalytic hydrogenation and reaction mixture obtained after electrocatalytic hydrogenation of **1h**.

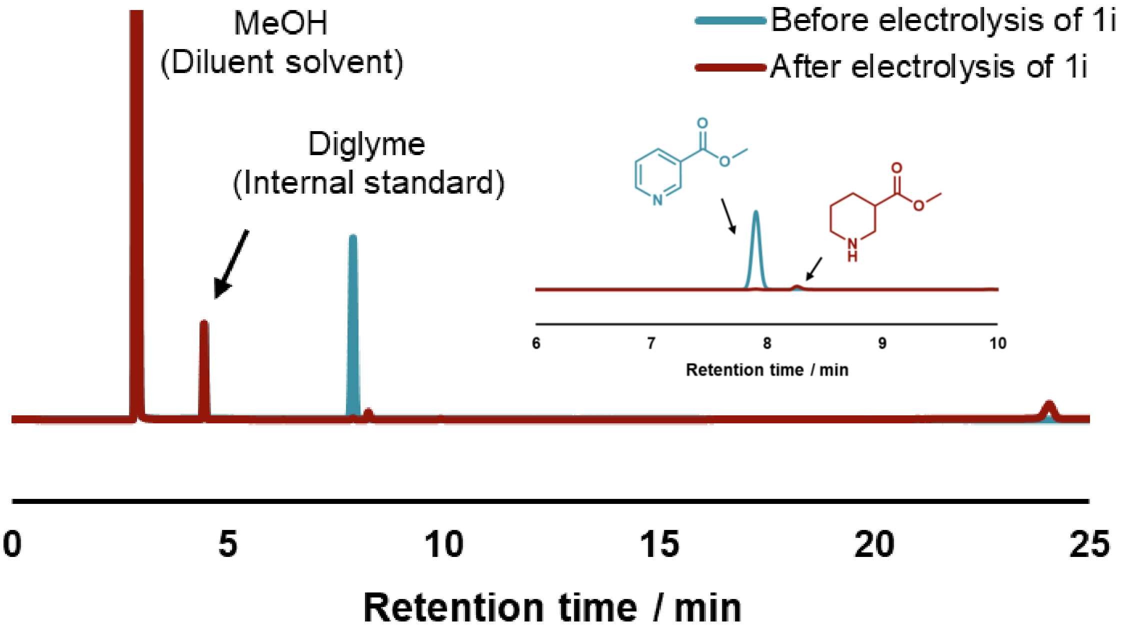

Figure S38 Gas chromatogram of the solution before electrocatalytic hydrogenation and reaction mixture obtained after electrocatalytic hydrogenation of **1i**.

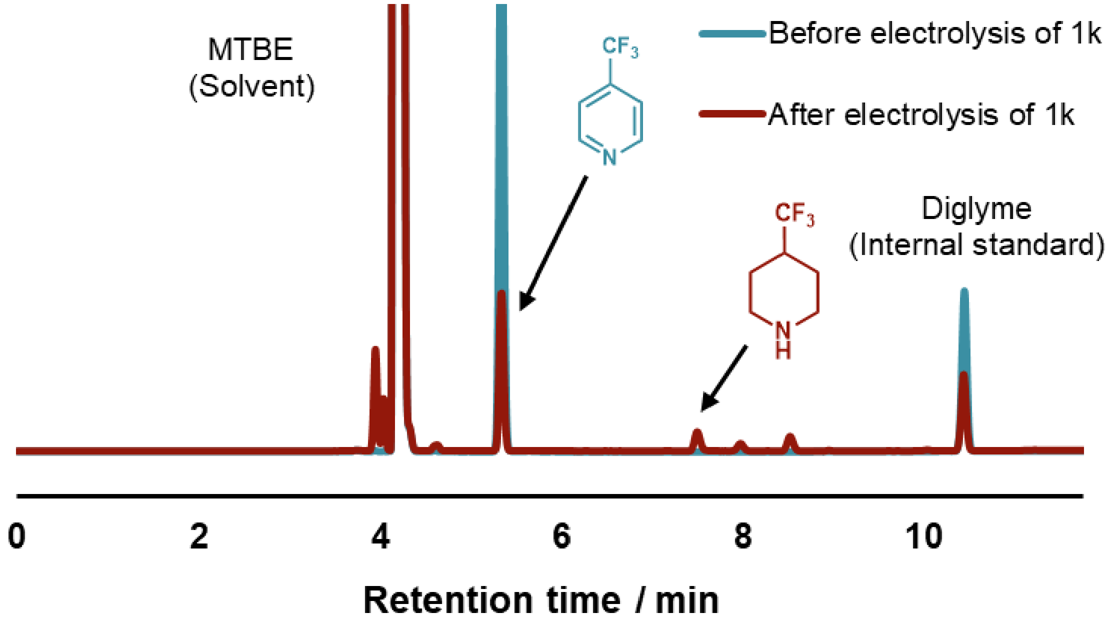

Figure S39 Gas chromatogram of the solution before electrocatalytic hydrogenation and reaction mixture obtained after electrocatalytic hydrogenation of **1k**.

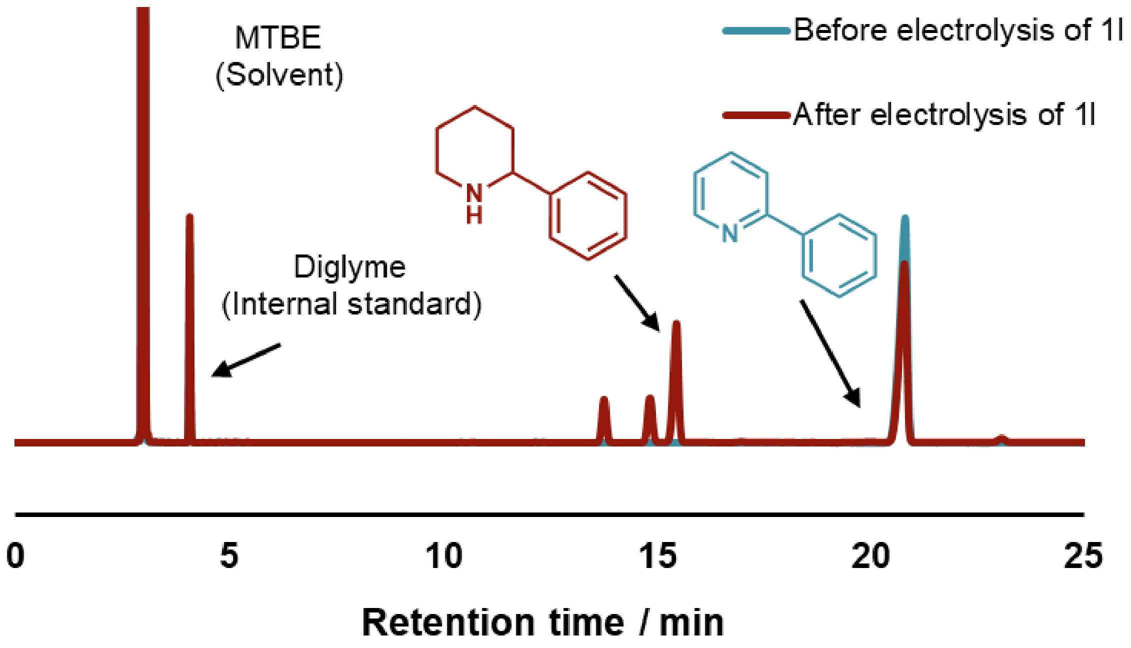

Figure S40 Gas chromatogram of the solution before electrocatalytic hydrogenation and reaction mixture obtained after electrocatalytic hydrogenation of **1l**.

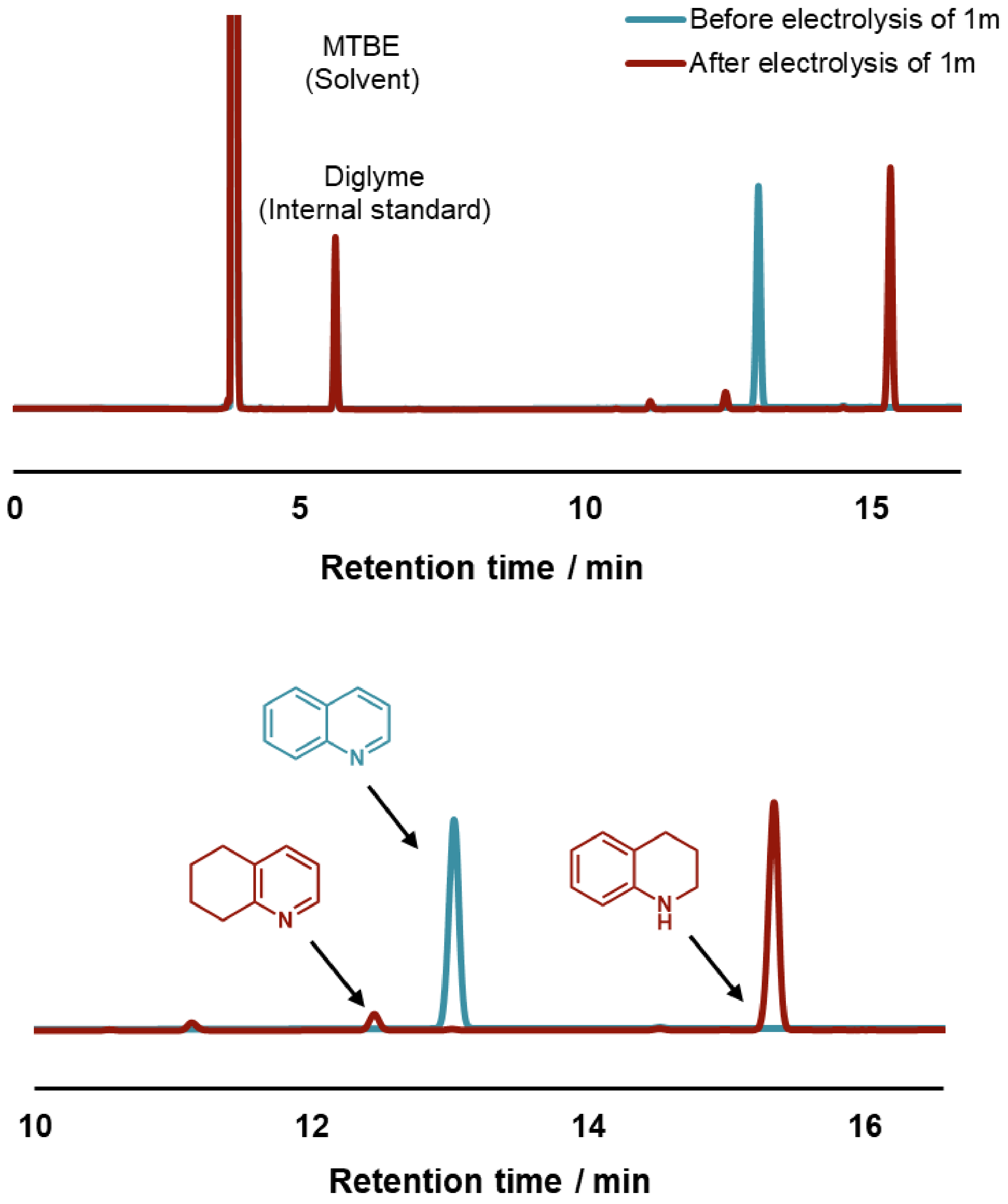

Figure S41 Gas chromatogram of the solution before electrocatalytic hydrogenation and reaction mixture obtained after electrocatalytic hydrogenation of **1m**.

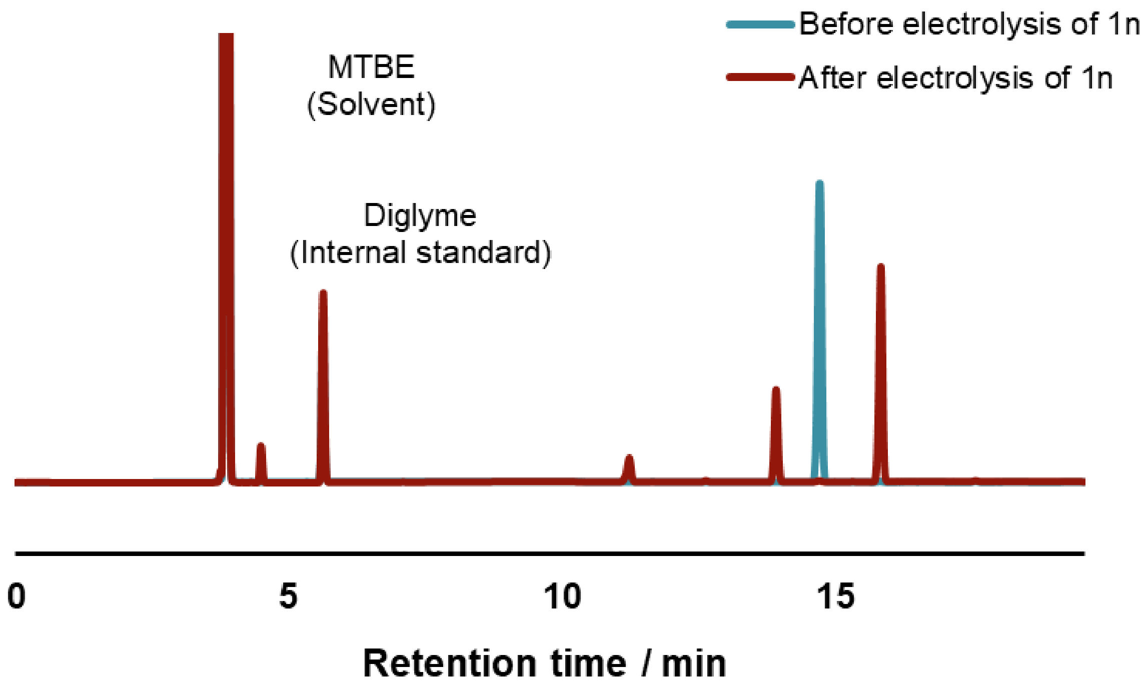

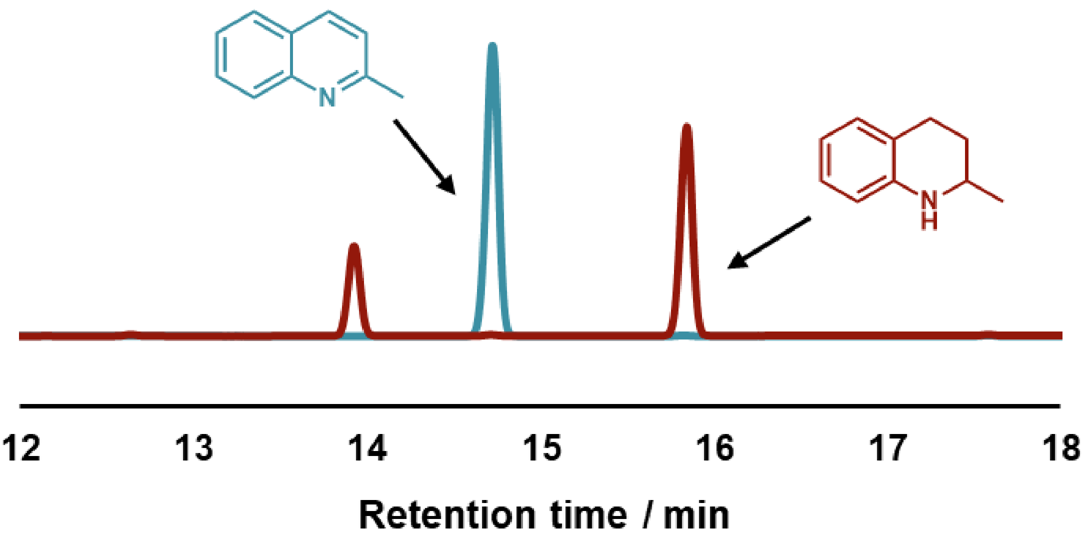

Figure S42 Gas chromatogram of the solution before electrocatalytic hydrogenation and reaction mixture obtained after electrocatalytic hydrogenation of **1n**.

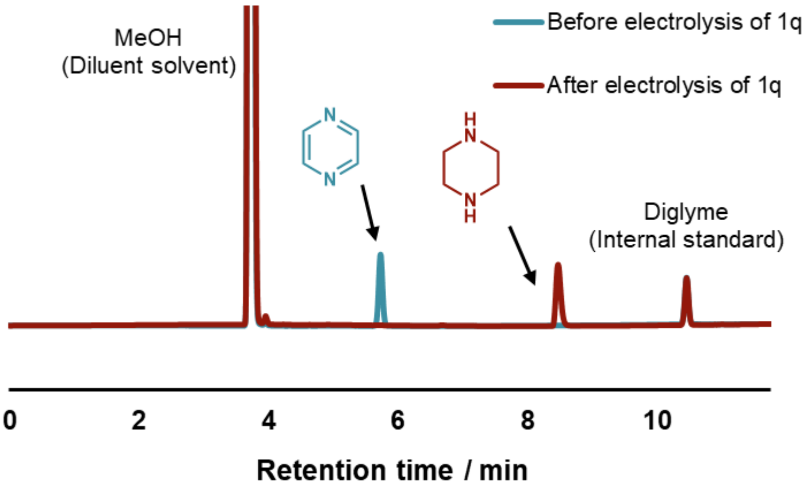

Figure S43 Gas chromatogram of the solution before electrocatalytic hydrogenation and reaction mixture obtained after electrocatalytic hydrogenation of **1q**.

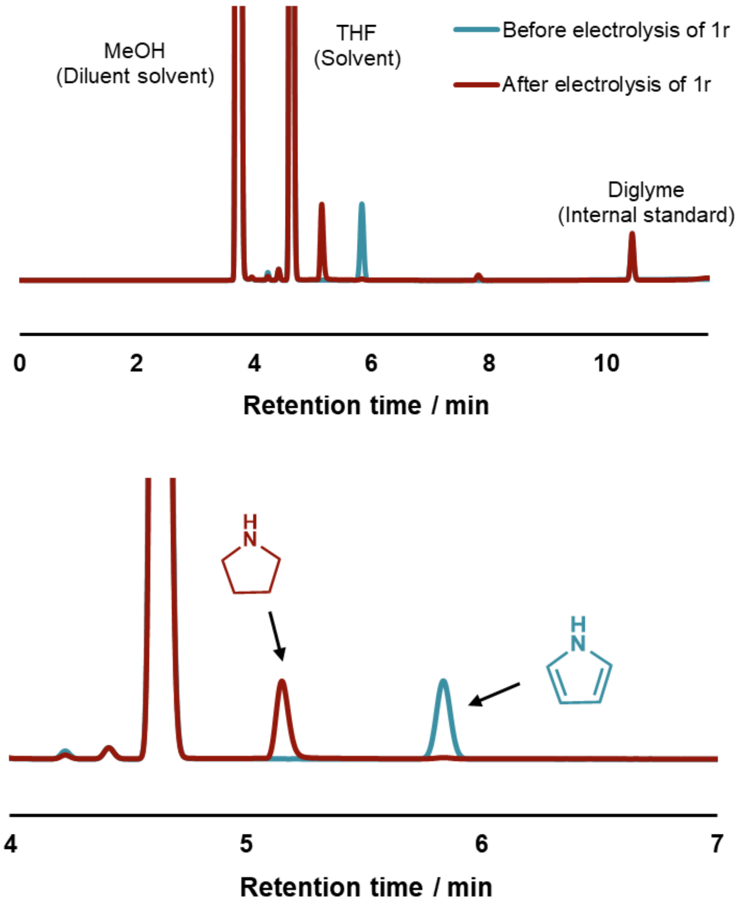

Figure S44 Gas chromatogram of the solution before electrocatalytic hydrogenation and reaction mixture obtained after electrocatalytic hydrogenation of **1r**.

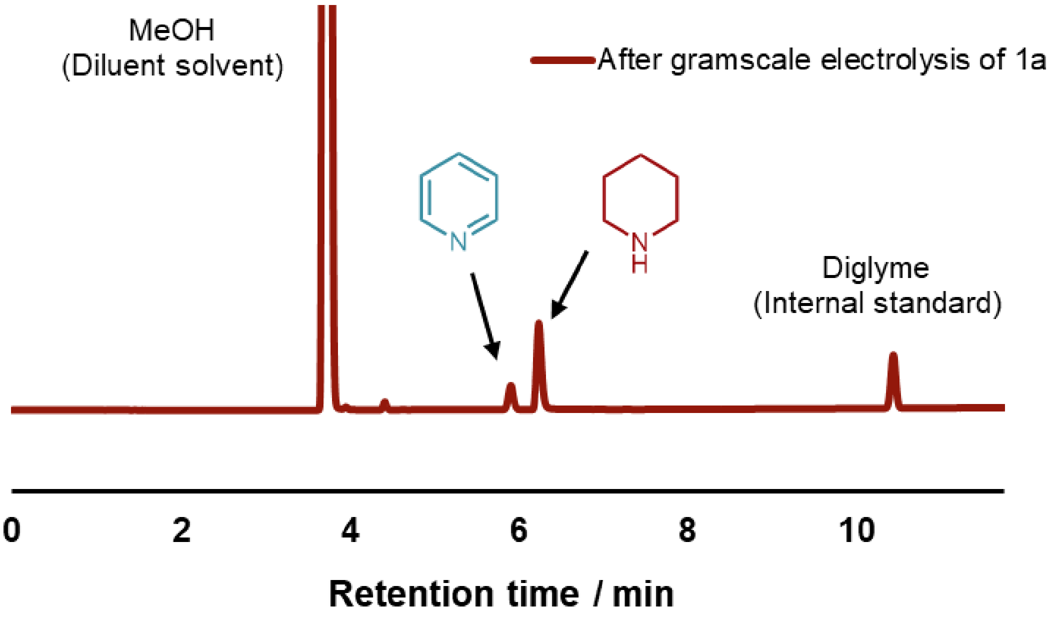

Figure S45 Gas chromatogram of the reaction mixture obtained after gram-scale electrocatalytic hydrogenation of **1a**.

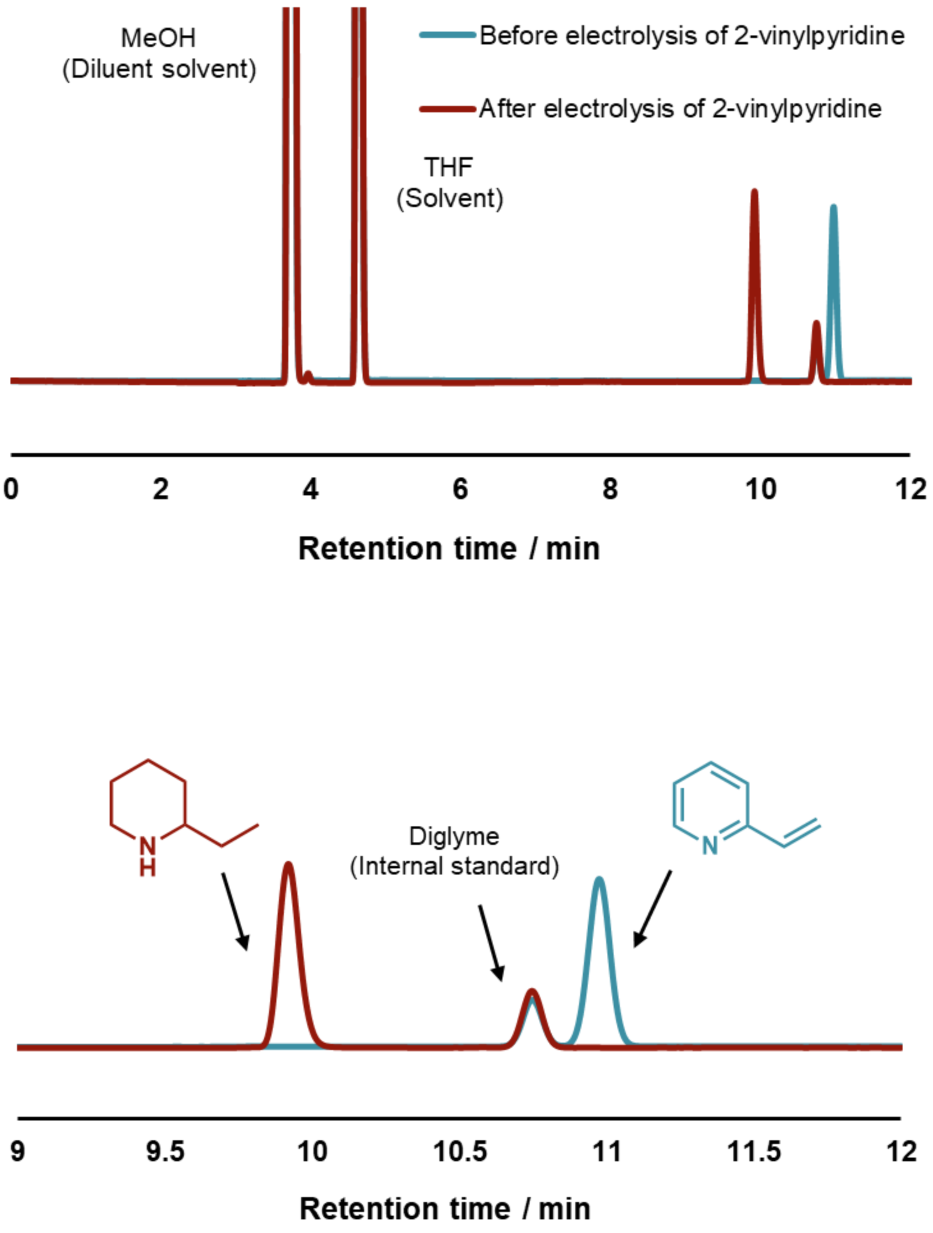

Figure S46 Gas chromatogram of the solution before electrocatalytic hydrogenation and reaction mixture obtained after electrocatalytic hydrogenation of **2-vinylpyridine**.

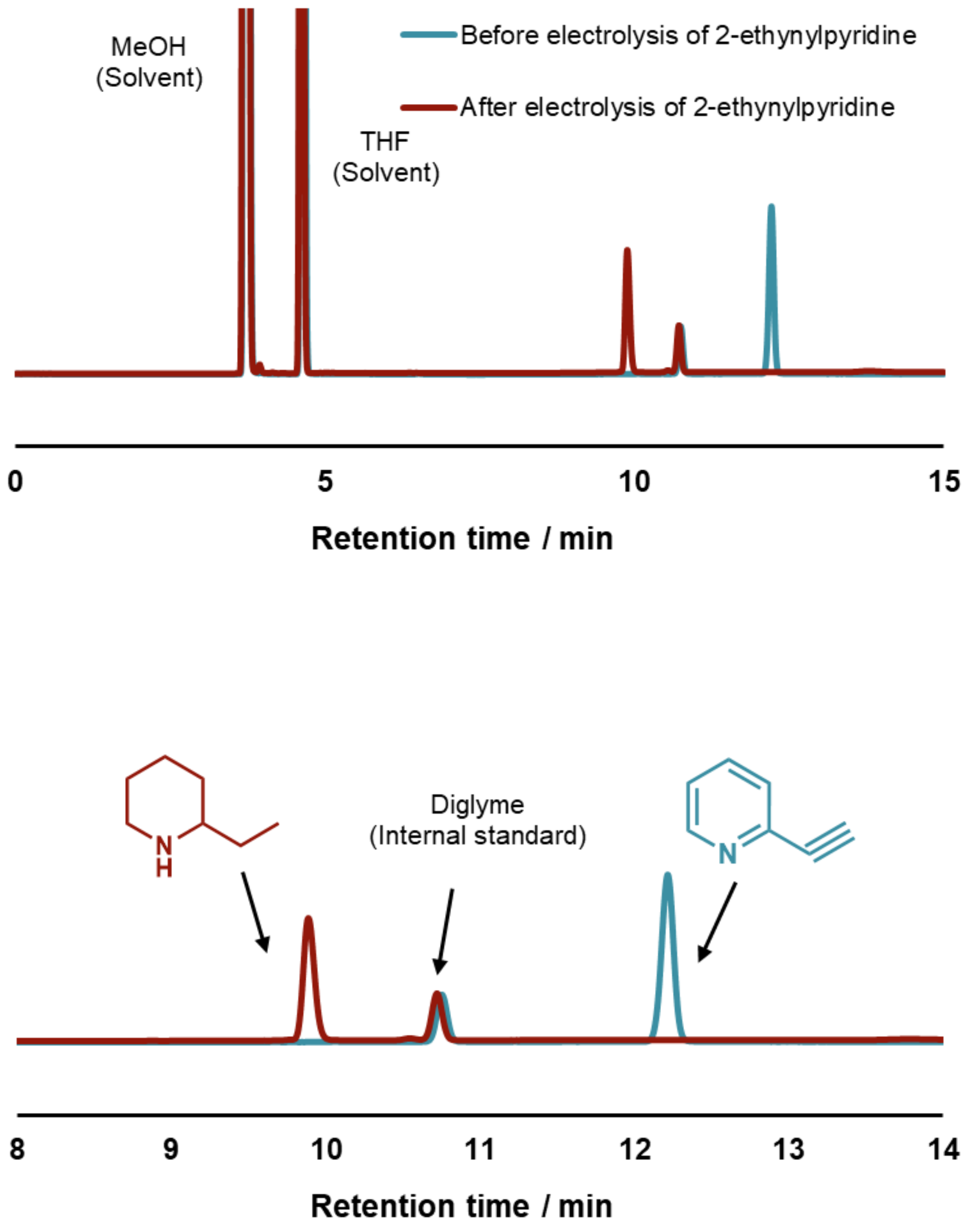

Figure S47 Gas chromatogram of the solution before electrocatalytic hydrogenation and reaction mixture obtained after electrocatalytic hydrogenation of **2-ethynylpyridine**.

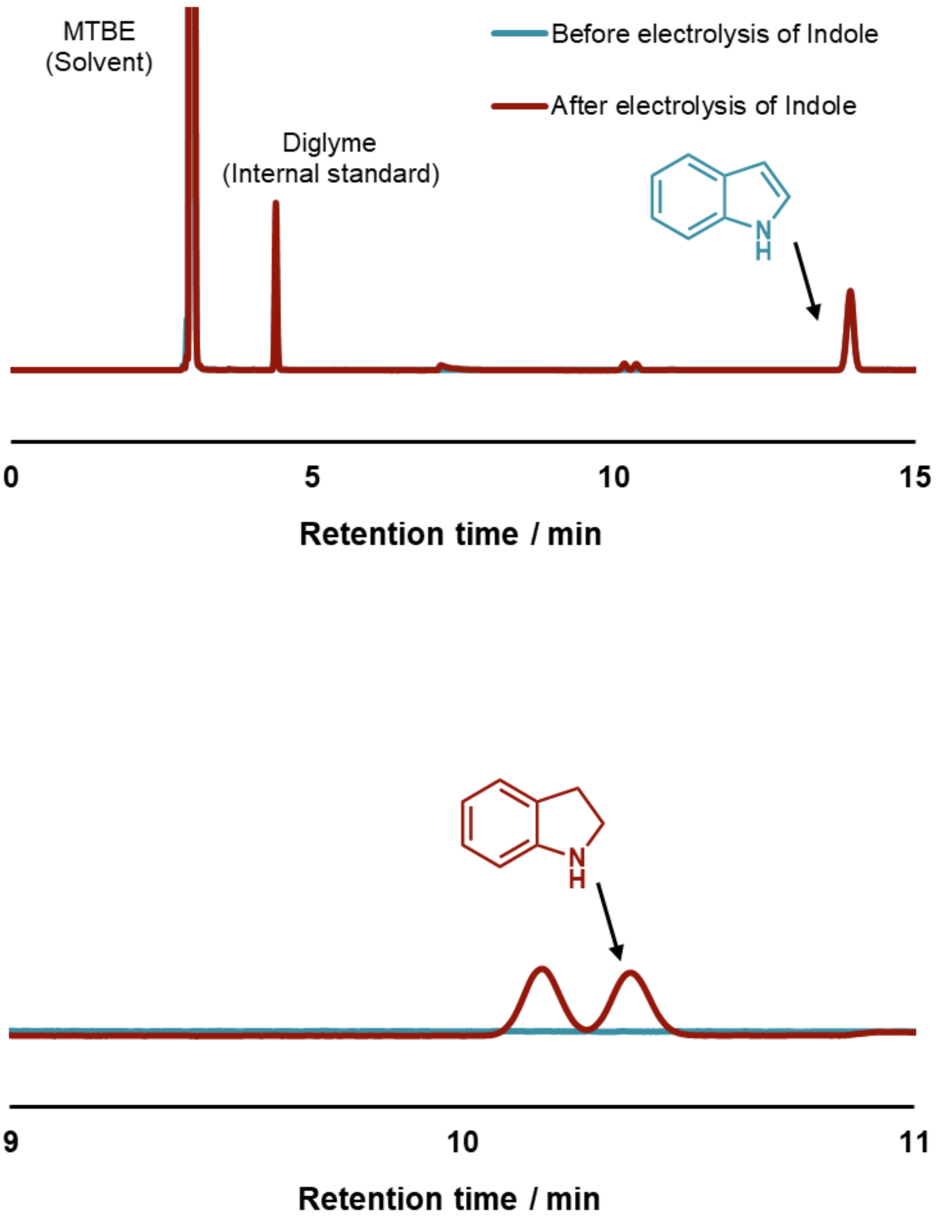

Figure S48 Gas chromatogram of the solution before electrocatalytic hydrogenation and reaction mixture obtained after electrocatalytic hydrogenation of **Indole**.

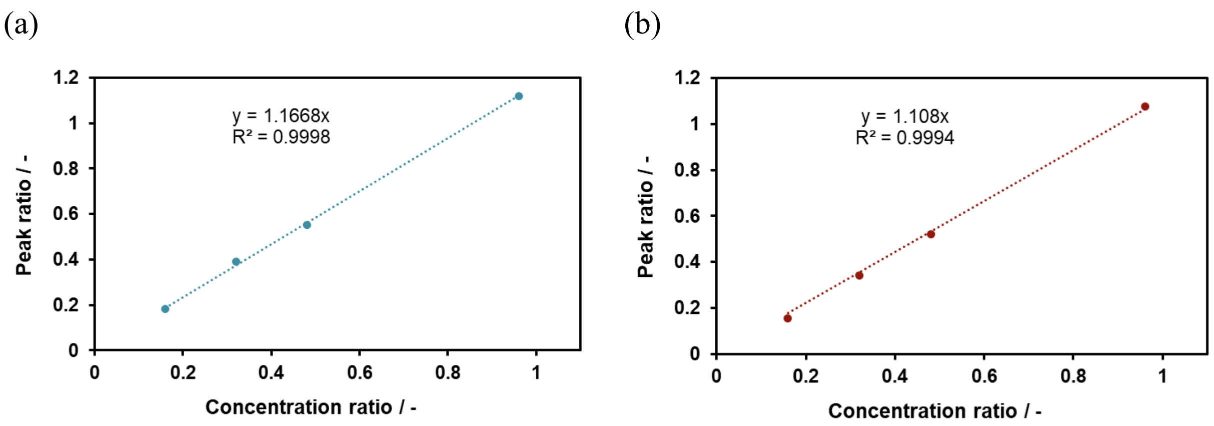

Figure S49 Calibration curve for (a) **1a** and (b) **2a** for GC analysis.

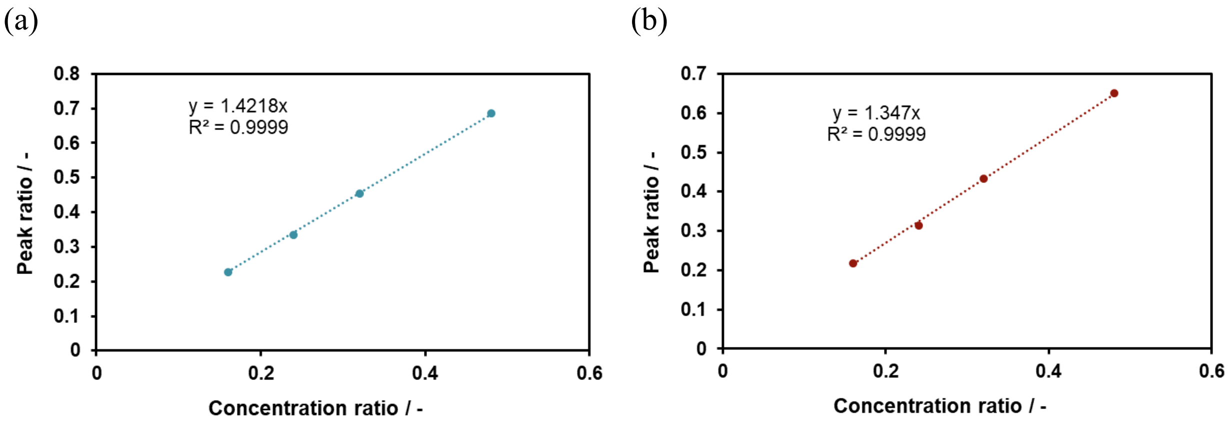

Figure S50 Calibration curve for (a) **1b** and (b) **2b** for GC analysis.

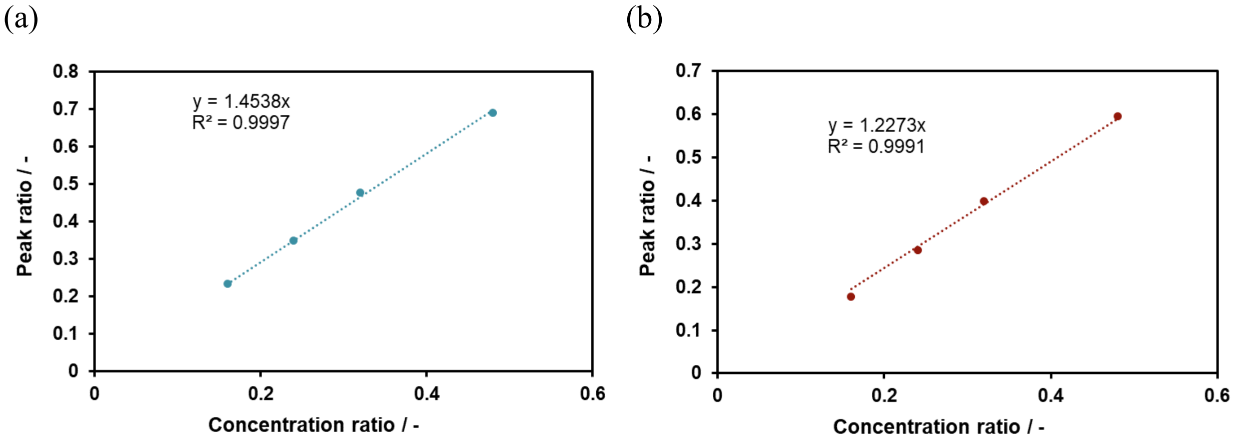

Figure S51 Calibration curve for (a) **1d** and (b) **2d** for GC analysis.

(a) (b)

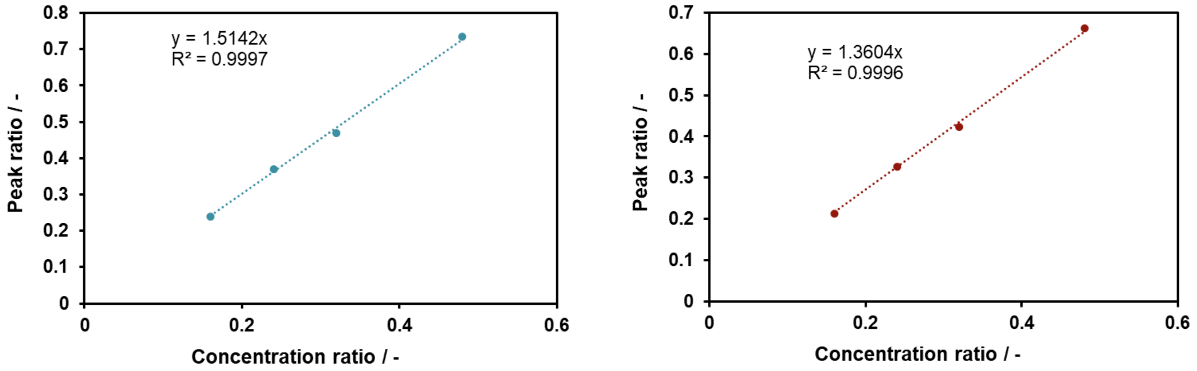

Figure S52 Calibration curve for (a) **1e** and (b) **2e** for GC analysis.

(a) (b)

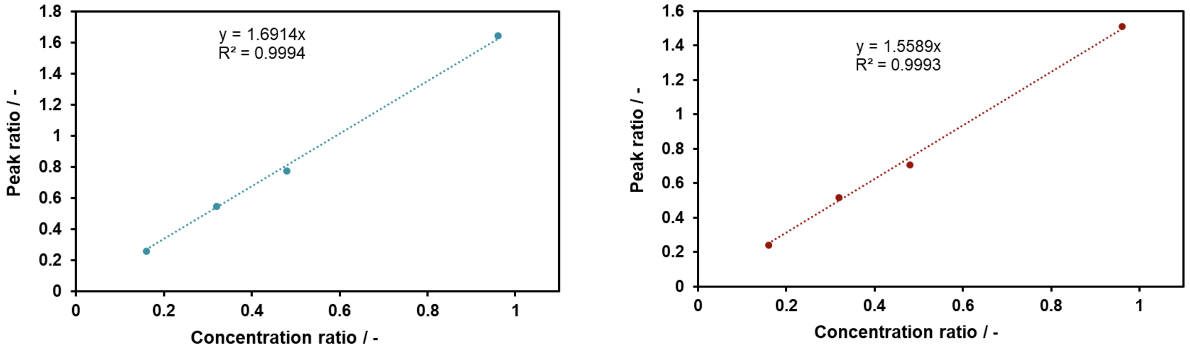

Figure S53 Calibration curve for (a) **1f** and (b) **2f** for GC analysis.

(a) (b)

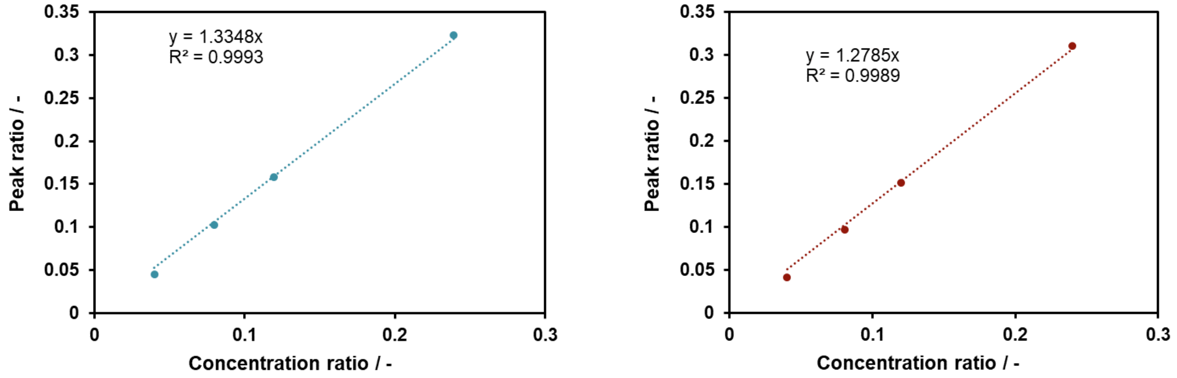

Figure S54 Calibration curve for (a) **1g** and (b) **2g** for GC analysis.

(a) (b)

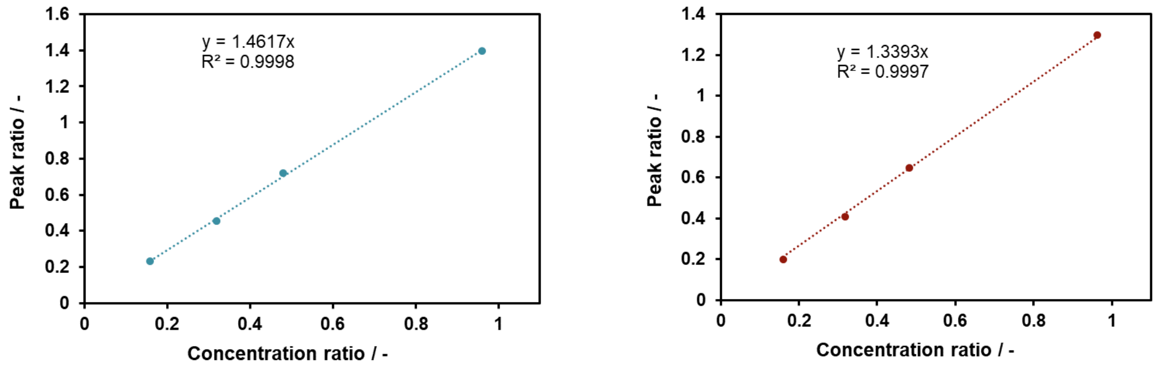

Figure S55 Calibration curve for (a) **1h** and (b) **2h** for GC analysis.

(a) **(b)**

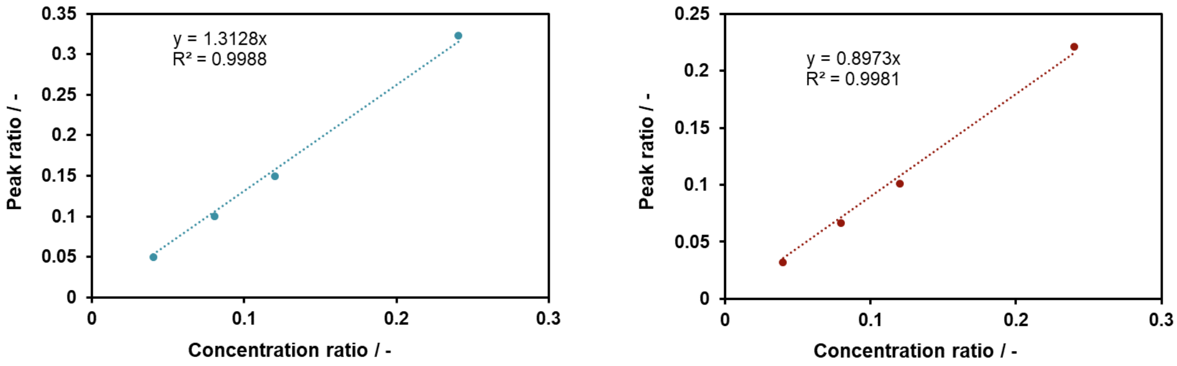

Figure S56 Calibration curve for (a) **1i** and (b) **2i** for GC analysis.

(a) (b)

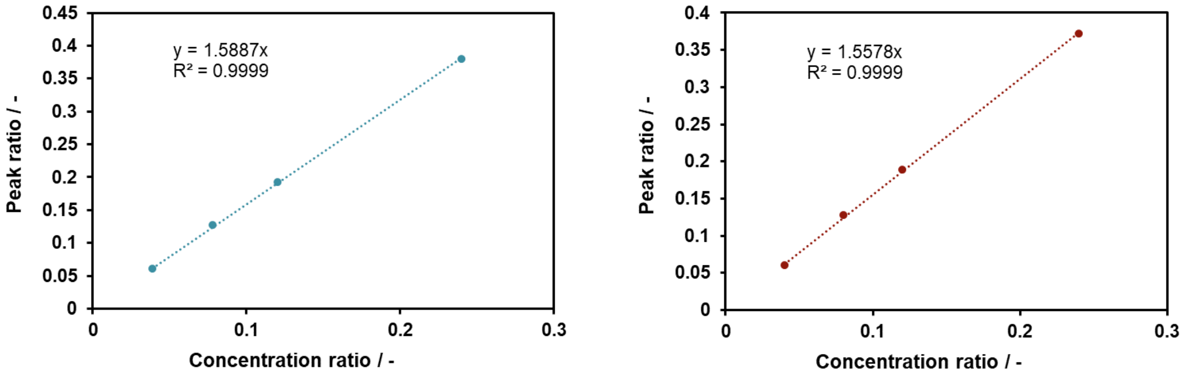

Figure S57 Calibration curve for (a) **1k** and (b) **2k** for GC analysis.

(a) (b)

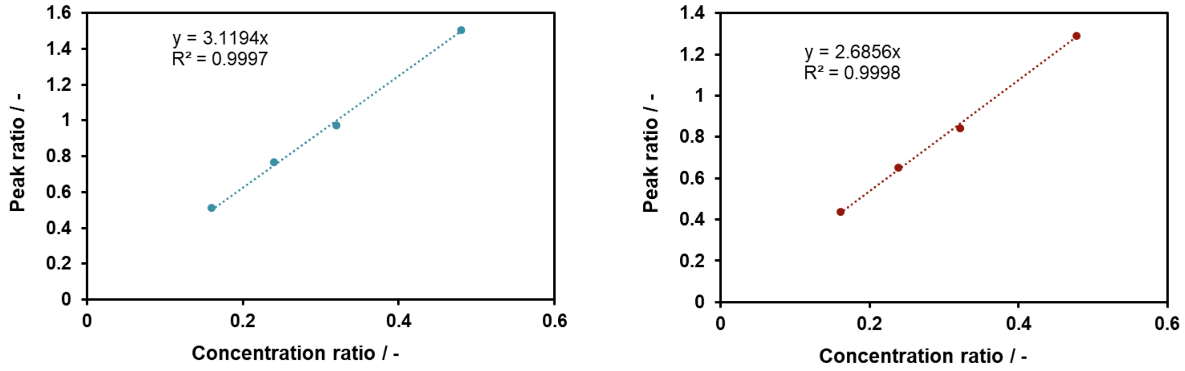

Figure S58 Calibration curve for (a) **1l** and (b) **2l** for GC analysis.

(a) (b)

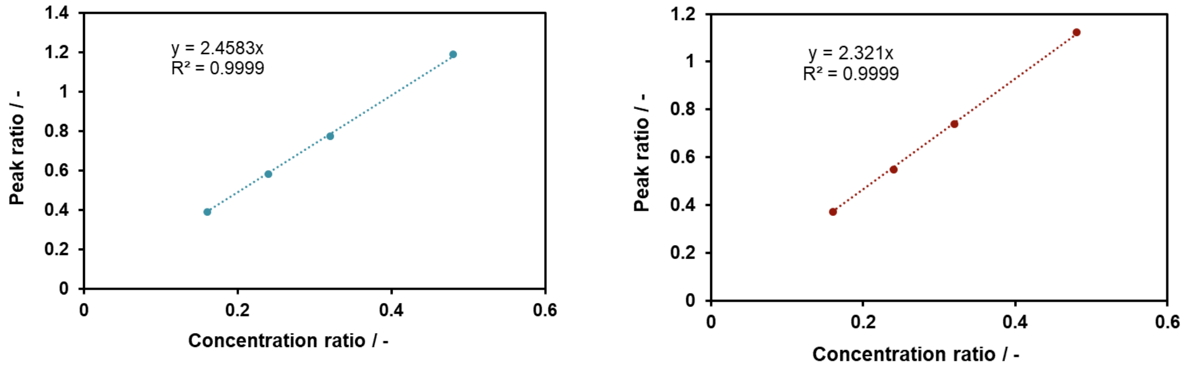

Figure S59 Calibration curve for (a) **1m** and (b) **2m** for GC analysis.

(a) (b)

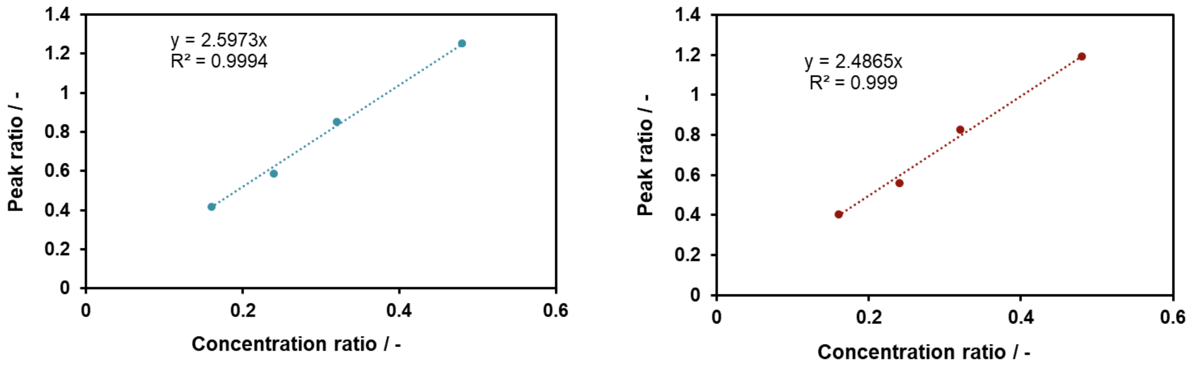

Figure S60 Calibration curve for (a) **1n** and (b) **2n** for GC analysis.

(a) (b)

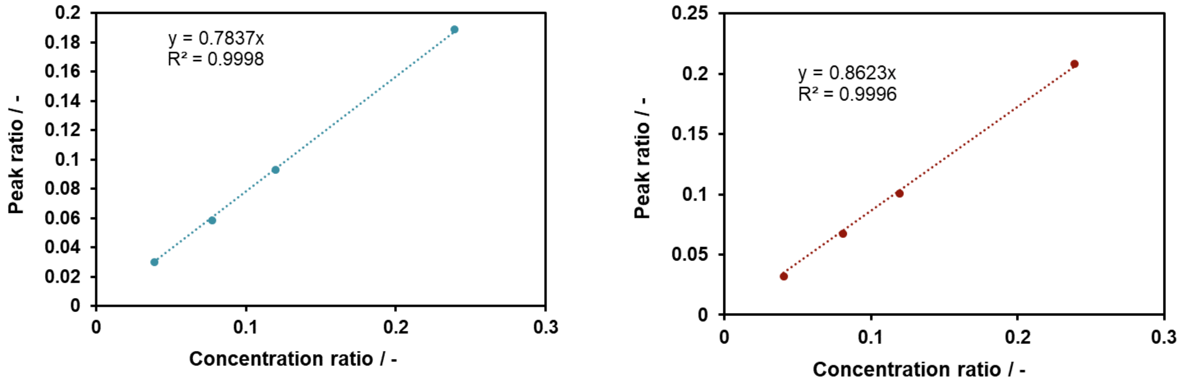

Figure S61 Calibration curve for (a) **1q** and (b) **2q** for GC analysis.

(a) (b)

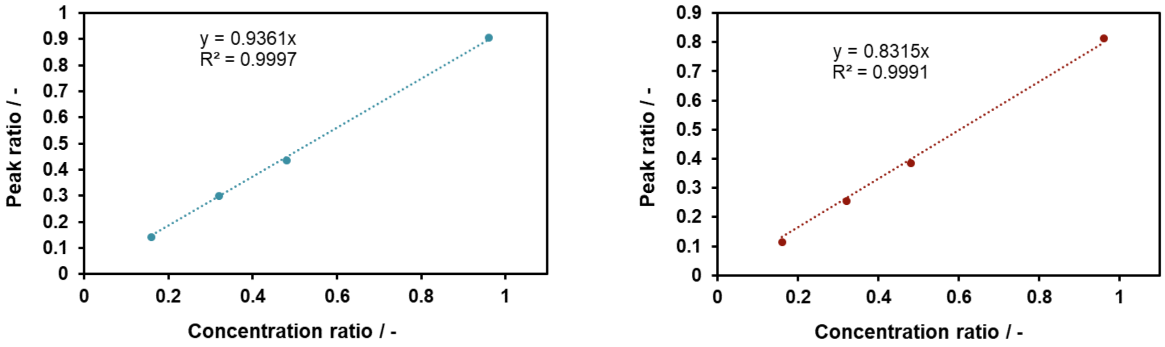

Figure S62 Calibration curve for (a) **1r** and (b) **2r** for GC analysis.

(a) (b)

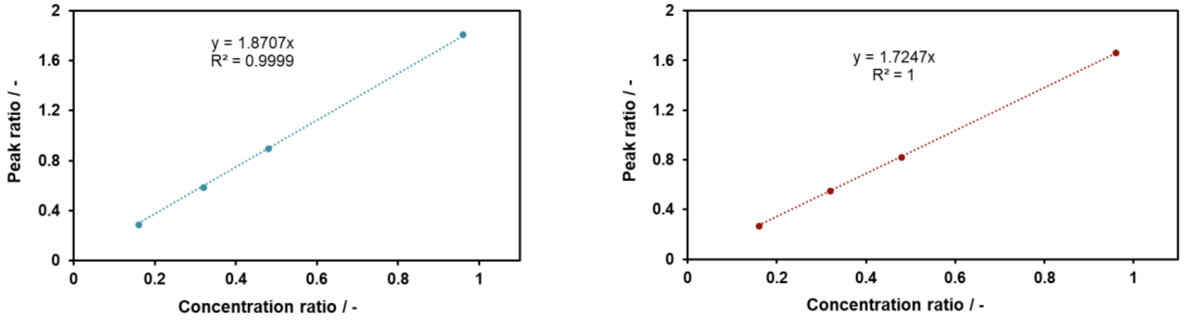

Figure S63 Calibration curve for (a) **2-vinylpyridine** and (b) **2c** for GC analysis.

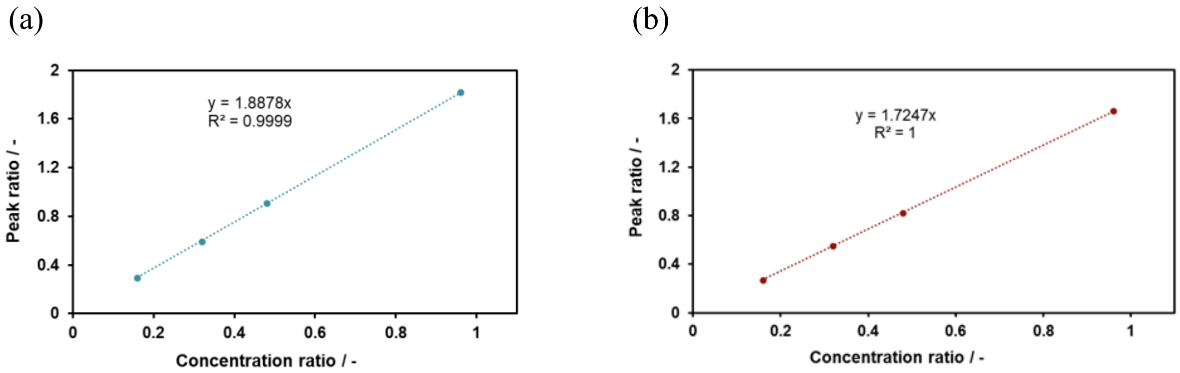

Figure S64 Calibration curve for (a) **2-ethynylpyridine** and (b) **2c** for GC analysis.

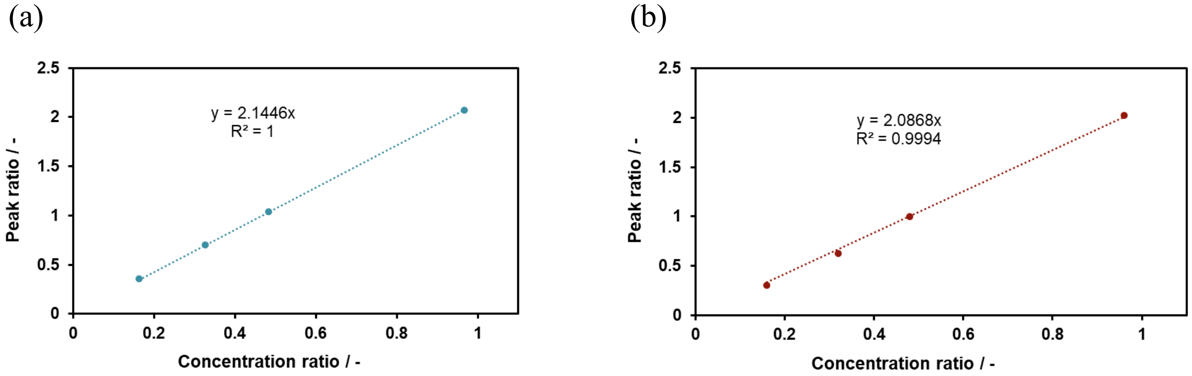

Figure S65 Calibration curve for (a) **Indole** and (b) **Indoline** for GC analysis.

16. High-performance liquid chromatograms

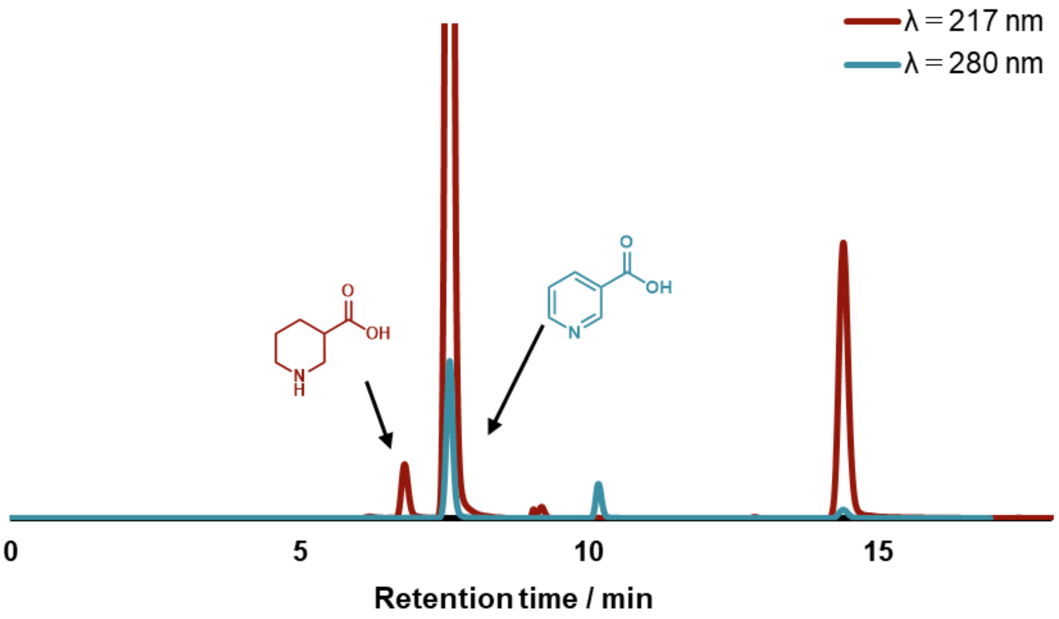

Figure S66 High performance liquid chromatogram of the reaction mixture obtained after electrocatalytic hydrogenation of **1j**.

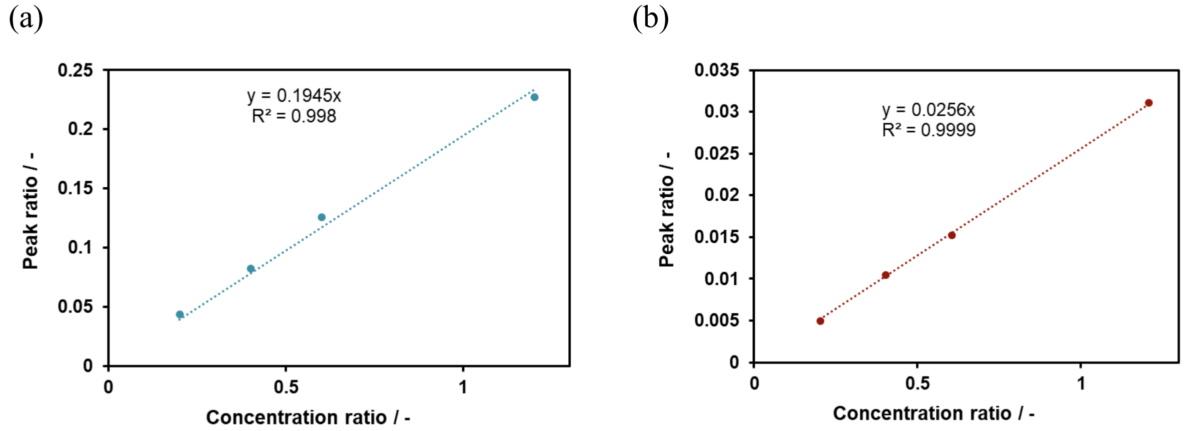

Figure S67 Calibration curve for (a) **1j** and (b) **2j** for HPLC analysis.

15. NMR spectral data

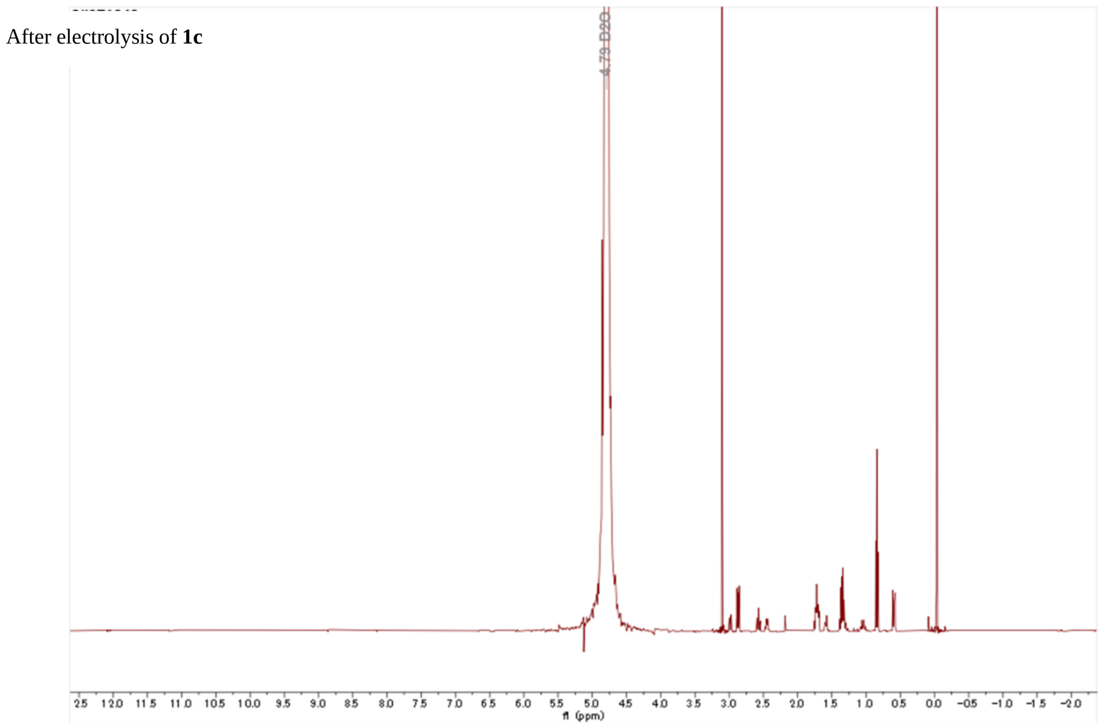

Figure S68-1 ^{1}H NMR spectrum (500 MHz, D_2O, 25 °C) of **1c**.

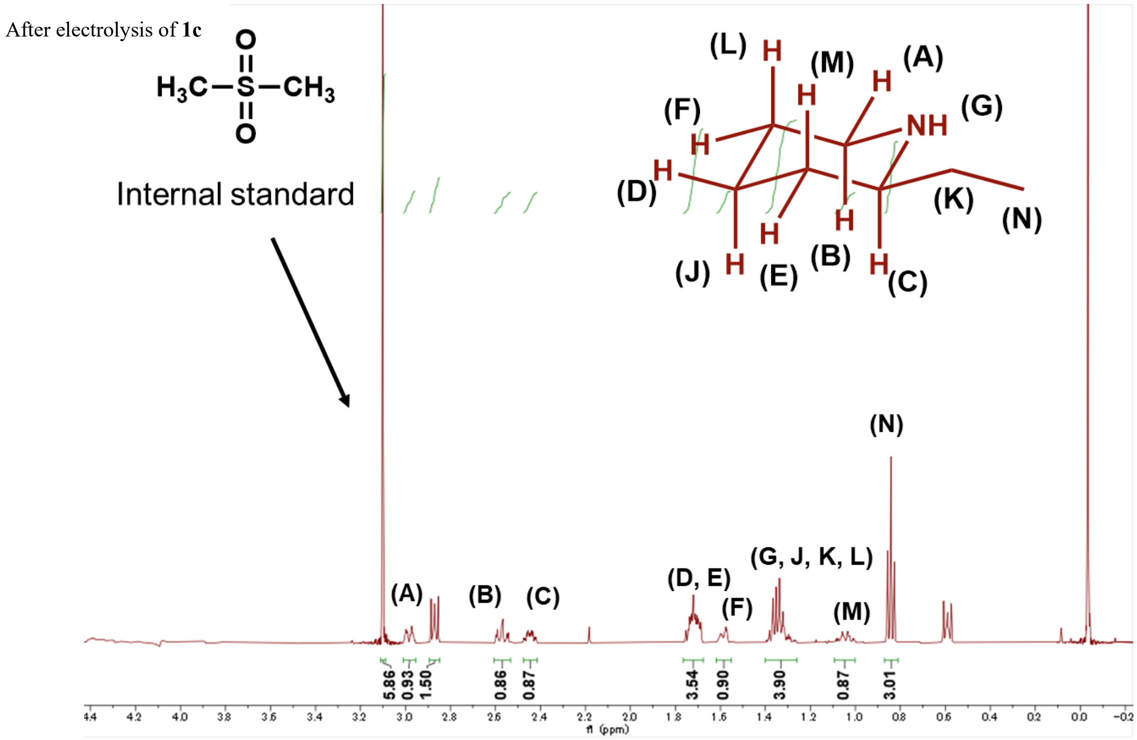

Figure S68-2 ^{1}H NMR spectrum (500 MHz, D_2O, 25 °C) of **1c**.[7]

After electrolysis of **1o**

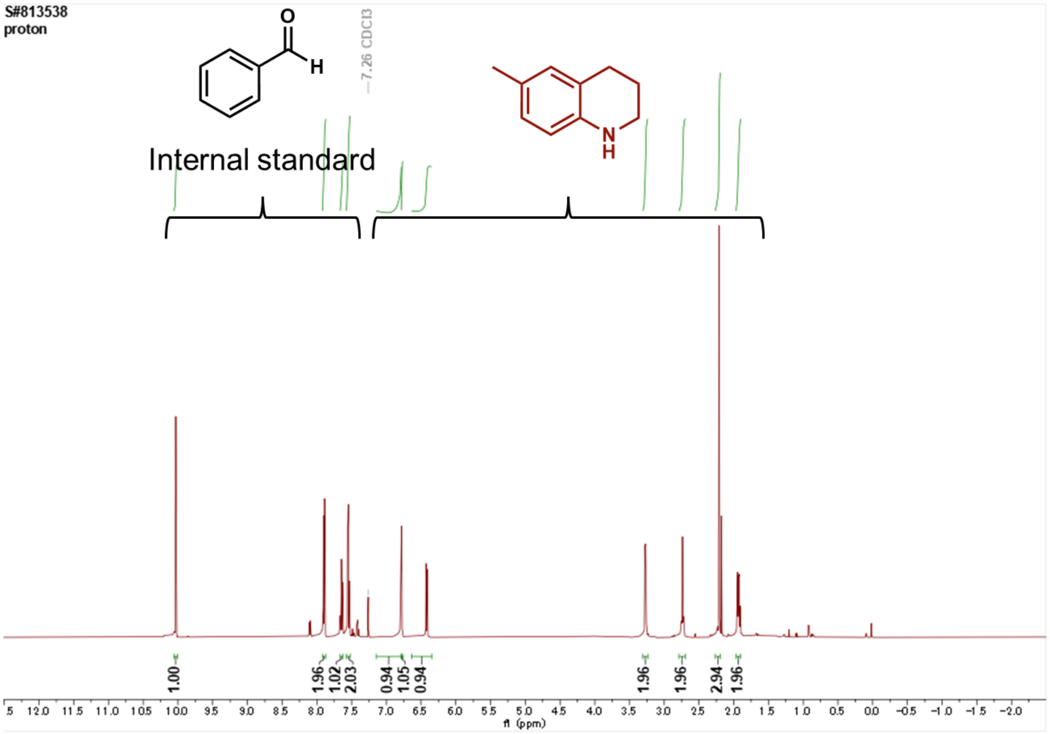

Figure S69 ^{1}H NMR spectrum (500 MHz, $CDCl_3$, 25 °C) of **1o.**[8–10]

After electrolysis of **1p**

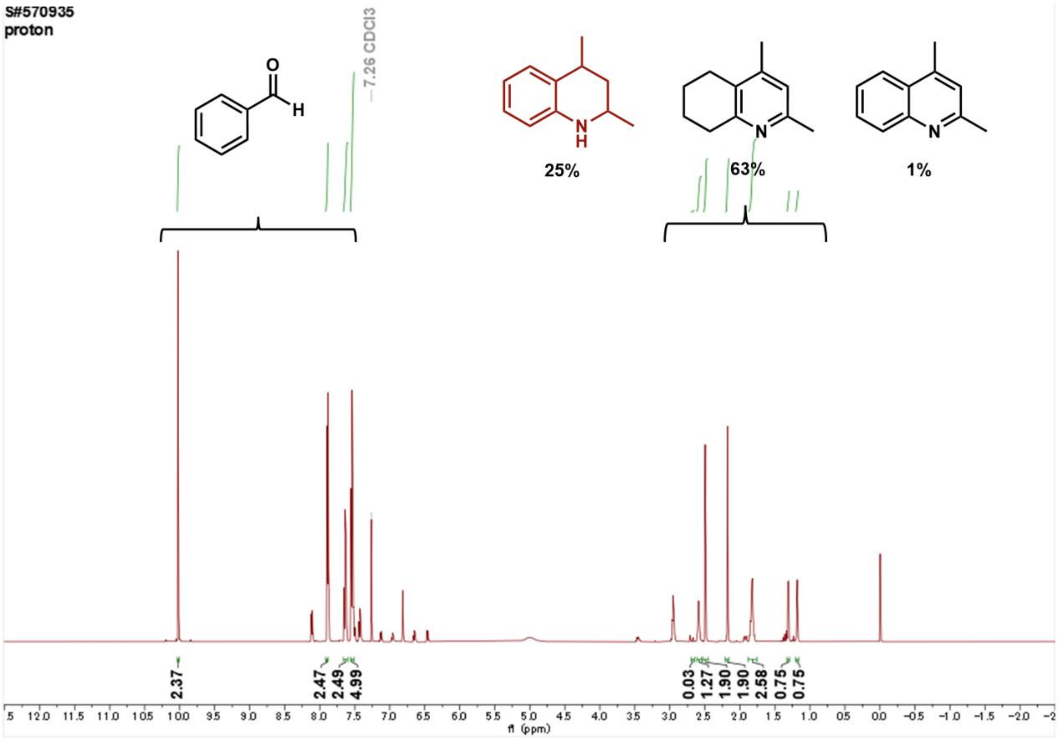

Figure S70 ^{1}H NMR spectrum (500 MHz, $CDCl_3$, 25 °C) of **1p.**[8,11,12]

16. Supplementary References

(1) Murugesan, K.; Chandrashekhar, V. G.; Kreyenschulte, C.; Beller, M.; Jagadeesh, R. V. A General Catalyst Based on Cobalt Core–Shell Nanoparticles for the Hydrogenation of N-heteroarenes Including Pyridines. *Angew. Chem. Int. Ed.* **2020**, *59* (40), 17408–17412.

(2) Qian, W.; Lin, L.; Qiao, Y.; Zhao, X.; Xu, Z.; Gong, H.; Li, D.; Chen, M.; Huang, R.; Hou, Z. Ru Subnanoparticles on N-Doped Carbon Layer Coated SBA-15 as Efficient Catalysts for Arene Hydrogenation. *Appl. Catal. A Gen.* **2019**, *585*, 117183.

(3) Martinez-Espinar, F.; Blondeau, P.; Nolis, P.; Chaudret, B.; Claver, C.; Castillón, S.; Godard, C. NHC-Stabilised Rh Nanoparticles: Surface Study and Application in the Catalytic Hydrogenation of Aromatic Substrates. *J. Catal.* **2017**, *354*, 113–127.

(4) Chen, F.; Li, W.; Sahoo, B.; Kreyenschulte, C.; Agostini, G.; Lund, H.; Junge, K.; Beller, M. Hydrogenation of Pyridines Using a Nitrogen-modified Titania-supported Cobalt Catalyst. *Angew. Chem. Weinheim Bergstr. Ger.* **2018**, *130* (44), 14696–14700.

(5) Ravel, B.; Newville, M. *ATHENA*, *ARTEMIS*, *HEPHAESTUS*: Data Analysis for X-Ray Absorption Spectroscopy Using*IFEFFIT*. *J. Synchrotron Radiat.* **2005**, *12* (4), 537–541.

(6) Domalski, E. S.; Hearing, E. D. Estimation of the Thermodynamic Properties of C-H-N-O *S*-Halogen Compounds at 298.15 K. *J. Phys. Chem. Ref. Data*, **1993**, *22*, 805–1159.

(7) "Integrated Spectral Data Base System of Organic Compounds" data were obtained from the National Institute of Advanced Industrial Science and Technology (Japan)

(8) Patel, P.; Nandi, S.; Menapara, T.; Biradar, A. V.; Nagarale, R. K.; Khan, N. H.; Kureshy, R. I. Glycoluril: A Heterogeneous Organocatalyst for Oxidation of Alcohols and Benzylic Sp3 Carbons. *Appl. Catal. A Gen.* **2018**, *565*, 127–134.

(9) Balayeva, N. O.; Mamiyev, Z.; Dillert, R.; Zheng, N.; Bahnemann, D. W. Rh/TiO_2-Photocatalyzed Acceptorless Dehydrogenation of *N*-Heterocycles upon Visible-Light Illumination. *ACS Catal.* **2020**, *10* (10), 5542–5553.

(10) Zhang, J.; An, Z.; Zhu, Y.; Shu, X.; Song, H.; Jiang, Y.; Wang, W.; Xiang, X.; Xu, L.; He, J. Ni^0/$Ni^{\delta+}$ Synergistic Catalysis on a Nanosized Ni Surface for Simultaneous Formation of C–C and C–N Bonds. *ACS Catal.* **2019**, *9* (12), 11438–11446.

(11) Han, S.; Chakrasali, P.; Park, J.; Oh, H.; Kim, S.; Kim, K.; Pandey, A. K.; Han, S. H.; Han, S. B.; Kim, I. S. Reductive C2-alkylation of Pyridine and Quinoline *N*-oxides Using Wittig Reagents. *Angew. Chem. Int. Ed.* **2018**, *57* (39), 12737–12740.

(12) Chen, F.; Surkus, A.-E.; He, L.; Pohl, M.-M.; Radnik, J.; Topf, C.; Junge, K.; Beller, M. Selective Catalytic Hydrogenation of Heteroarenes with *N*-Graphene-Modified Cobalt Nanoparticles (Co_3O_4–Co/NGr@α-Al_2O_3). *J. Am. Chem. Soc.* **2015**, *137* (36), 11718–11724.
